# *GmPGL1*, a Thiamine Thiazole Synthase, Is Required for the Biosynthesis of Thiamine in Soybean

**DOI:** 10.3389/fpls.2019.01546

**Published:** 2019-11-22

**Authors:** Xingxing Feng, Suxin Yang, Kuanqiang Tang, Yaohua Zhang, Jiantian Leng, Jingjing Ma, Quan Wang, Xianzhong Feng

**Affiliations:** ^1^Key Laboratory of Soybean Molecular Design Breeding, Northeast Institute of eography and Agroecology, The Innovative Academy of Seed Design, Chinese Academy of Sciences, Changchun, China; ^2^College of Advanced Agricultural Sciences, University of Chinese Academy of Sciences, Beijing, China

**Keywords:** soybean, GmPGL1, thiamine thiazole synthase, carbohydrate synthesis, amino acid synthesis

## Abstract

Thiamine is an essential cofactor in several enzymatic reactions for all living organisms. Animals cannot synthesize thiamine and depend on their diet. Enhancing the content of thiamine is one of the most important goals of plant breeding to solve the thiamine deficiency associated with the low-thiamin staple crops. In this study, a *Glycine* *max* *pale* *green* *leaf 1* (*Gmpgl1*) mutant was isolated from the EMS mutagenized population of soybean cultivar, Williams 82. Map-based cloning of the *GmPGL1* locus revealed a single nucleotide deletion at the 292th nucleotide residue of the first exon of *Glyma.10g251500* gene in *Gmpgl1* mutant plant, encoding a thiamine thiazole synthase. Total thiamine contents decreased in both seedlings and seeds of the *Gmpgl1* mutant. Exogenous application of thiazole restored the pale green leaf phenotype of the mutant. The deficiency of thiamine in *Gmpgl1* mutant led to reduced activities of the pyruvate dehydrogenase (PDH) and pyruvate decarboxylase (PDC), and decreased contents of six amino acids as compared to that in the wild type plants. These results revealed that *GmPGL1* played an essential role in thiamine thiazole biosynthesis.

## Introduction

Thiamine is a water-soluble vitamin essential to all organisms. Thiamine is widely available in green vegetables, beans, cereal embryos and nuts. However, humans and animals are incapable of synthesizing thiamine, and depend entirely on plant sources for this vitamin (Goyer, 2010). Thiamine deficiency leads to various chronic diseases in humans, such as edema, neurological disorders and beriberi (Rapala-Kozik, 2011). Thiamin esters are available as thiamin, thiamin monophosphate (TMP) and thiamine diphosphate (TPP) (Gangolf et al., 2010). TPP is the functional form of thiamine and takes part as a cofactor in plant central energy metabolizing enzymes (Mimura et al., 2016), which are pyruvate dehydrogenase (PDH) of glycolysis, α-ketoglutarate dehydrogenase (α-KGDH) of the tricarboxylic acid cycle, transketolase (TK) of the Calvin Benson cycle. It is also a cofactor in some enzymatic reactions, such as (i) in aerobic energy metabolism, (ii) carbohydrate catabolism, (iii) the pentose phosphate pathway and (iv) branched-chain amino acid biosynthesis (Linka and Weber, 2010; Bocobza et al., 2013; Khozaei et al., 2016). The major cereals, such as wheat, rice and corn, contain inadequate levels of this vitamin after processing and can lead to thiamine deficiencies. Vitamins has been selected as the target in the fight against micronutrient malnutrition in humans (Strobbe and Van Der Straeten, 2018).

The thiamine biosynthetic pathway in plants is similar to that in prokaryotes (Jurgenson et al., 2009). Thiamine is assembled from pyrimidine and thiazole moieties, which are synthesized independently (Goyer, 2010). The THIC (4-amino-5-hydroxymethyl-2-methylpyrimidine phosphate synthase) protein catalyzes synthesis of pyrimidine moiety (4-amino-2-methyl-5-hydro-xymethylpyrimidine monophosphate, HMP-P) of thiamine from 5-aminoimidazole ribonucleotide (AIR). THIC is localized to the stroma of chloroplasts (Kong et al., 2008). THI1 catalyzes the synthesis of thiazole moiety (4-methyl-5- (2-hydroxyethyl)-thiazole phosphate, HET-P) from glycine, NAD^+^ and sulfur. THI1 is the only enzyme involved in thiazole biosynthesis (Papini-Terzi et al., 2003; Yazdani et al., 2013). In plants, thiamine deficiency leads to development of chlorotic leaves (Ajjawi et al., 2007a; Rapala-Kozik et al., 2007).

The *THI1* gene was identified from *Arabidopsis thaliana*, which complemented bacterial defects in DNA repair (Machado et al., 1996). In *thi1* mutants, the rosette leaves are yellow and requires on exogenous thiamine application for its survival (Papini-Terzi et al., 2003). *THI1* gene is highly expressed in dividing tissues and regulates organ formation (Woodward et al., 2010). THI1 protein is typically modified at the N-terminus with signals for targeting to chloroplast and mitochondria (Chabregas et al., 2001). In *Arabidopsis thaliana*, *th1*, *th2*, *th3*, *py* and *tz* mutants requiring thiamine for survival have been isolated. In *th1*, *th2* and *th3*, leaves are white and they require thiamine supplementation to become normal; whereas, *py* and *tz* can be rescued with an exogenous supply of HMP and HET, respectively (Feenstra, 1964; Redei, 1965). The three-dimensional structure of the *Arabidopsis* THI1 protein was resolved by Godoi et al. (2006). The protein assembles as an octamer, and the protein complex is a potential intermediate of thiazole biosynthesis. The octamer is tightly packed as a two-layer ring torus structure from four dimers. Proteomics studies have indicated that THI1 contains two to three conserved cysteine residues and is a potential target of chloroplast thioredoxin (Lemaire et al., 2004). *THI1* encodes HET-P synthase, which is essential for thiamine biosynthesis. HET-P synthase has been detected in chloroplast and mitochondria in *Z. mays* and *Arabidopsis* (Belanger et al., 1995; Chabregas et al., 2003). HET-P synthase targeted to mitochondrial protect any DNA damage, while HET-P synthase targeted to chloroplasts is used in biosynthesis of thiamine (Ajjawi et al., 2007b). Vitamins can reinforce plant disease resistance and environmental stress tolerance, as well as act directly as exert antimicrobial activities (Hong et al., 2016; Huang et al., 2016). And it was recently reported that TPP also played a role in metabolic acclimation to the photoperiod (Rosado-Souza et al., 2019).

Here, we report the characterization of the *Glycine* *max* *pale* *green* *leaf 1* (*Gmpgl1*) mutant. The *GmPGL1* gene encodes a thiamine thiazole synthase (*THI1*). Pale green leaves of *Gmpgl1* mutant seedling are rescued by supplementation with thiazole. Activities of TPP-dependent enzymes pyruvate dehydrogenase and pyruvate decarboxylase decreased in the *Gmpgl1* mutant as compared to the wild type Williams 82. The metabolic network of amino acid synthesis and carbohydrate synthesis strongly affected in *Gmpgl1*. The presented results suggested that *GmPGL1* gene was involved in biosynthesis of thiamine in soybean.

## Materials and Methods

### Plant Materials and Growth Conditions

We mutagenized 10,000 dry seeds of Williams 82 with 0.6% ethyl methyl sulfonate (EMS) in April 2013. The mutagenized seeds were grown in the Chang-Chun experiment filed of Northeast Institute of Geography and Agroecology, CAS in May 2013 and 4,354 M_2_ lines were harvested in October 2013. Twenty progeny of each M_2_ line were sowed in the field for phenotyping during May to October 2014. The screened mutant plants were backcrossed for four generations to purify the genetic background from July 2014 to October 2016 in both greenhouse and filed as described before (Chen et al., 2016). One of pale green leaf mutant, named as *Glycine* *max* *pale* *green* *leaf 1* (*Gmpgl1*), was selected to mapping the mutation locus and function study.

Thiazole was exogenously applied as described by earlier (Hsieh et al., 2017) with minor modification. The seeds were germinated with H_2_O for two days in dark at 25°C, and then transferred into Hoagland nutrient solution, supplemented thiazole (Shanghai Macklin Biochemical Co. Ltd) with 15 mM for five days in a growth chamber under a 16 h/8 h light dark cycle at 30–25°C. Control plants were treated with H_2_O instead of thiazole. Three plants were investigated in each treatment.

### Mapping of *GmPGL1* Using Bulked-Segregant Analysis

F_2_ plants derived from a cross between the *Gmpgl1* mutant and a Chinese local cultivar, Hedou12, were used to map the *GmPGL1* gene (Song et al., 2015). DNA from 32 F_2_ individuals with the *Gmpgl1* mutant phenotype and 50 F_2_ individuals with wild-type phenotype were bulked into mutant and wild-type pools, respectively. These DNA samples were used to construct libraries that were subjected to whole-genome sequencing using the Illumina HiSeq™ 2500 platform. Single-nucleotide polymorphisms (SNPs) and small InDels were calculated between the *Gmpgl1* and Williams 82 bulked DNA samples by aligning the sequence reads of individual bulked DNA samples to the *Glycine max* Wm82.a2.v1 reference genome (https://phytozome.jgi.doe.gov/pz/portal.html). The candidate genomic regions were identified by QTL-seq method using bulked segregants from F_2_ population (Takagi et al., 2013). Sliding-window analysis with a 1 Mb window size and with an 100 kb increment for all chromosomes of soybean genome was conducted to develop the SNP index plots. The allele frequencies were calculated based on the formula SNP index = SNP Williams 82/(SNP Williams 82 + SNP Hedou12), and △(SNP-index) = (SNP-index of mutant-bulk)-(SNP-index of wild type-bulk). The genomic region with △(SNP-index) > 0.5 was selected as candidate area.

### RNA Isolation and Real-Time Quantitative Polymerase Chain Reaction Analyses

Total RNA was extracted from the soybean tissue samples using Trizol reagent (Tiangen) according to the manufacturer's instructions. The first strand of cDNA was performed using the PrimeScript™ RT reagent kit (Takara, RR014) following the manufacture's methods. Quantitative real-time polymerase chain reaction (qRT-PCR) was performed using a SYBR^®^ Premix Ex Taq™ Kit (Takara, RR420) on Stratagene MX3005P Real-Time PCR System (Agilent Technologies, Inc., Santa Clara, CA) according to the manufacturer's instructions. The qRT-PCR procedure was conducted as follows: 10 min at 95°C, followed by 40 cycles of 95°C for 10 s, 58°C for 20 s, and 72°C for 20 s. Three independent biological replications were performed for each sample to calculate the relative expression level using the 2^−ΔΔCt^ method after normalization to *Cons4* (ATP binding cassette transporter gene, *Glyma.12G020500*) (Libault et al., 2008). The primer pairs are listed in **Table S1**.

### Plasmid Construction and Soybean Transformation

To generate *GmPGL1* knock out plants, CRISPR/Cas system for targeted genome modification of crop plants was used (Shan et al., 2013). A pair of 23 bp 5′-ATTGAGCAGTCCGTGAGCCCCGG-3′, 5′-AACCCGGGGCTCACGGACTGCTC-3′ oligonucleotides specific to *GmPGL1* was annealed and cloned into the modified *VK005-04-soU6-2-GmUbi3* vector in the *BspQ*I endonuclease restriction site (Du et al., 2016). The knock out construct was introduced into *Agrobacterium tumefaciens EHA105*, which was then use to transform cotyledonary explants of the 'Dongnong 50' (Zhao et al, 2016; Dai et al, 2018).

### Database Searching and Phylogenetic Analysis

GmPGL1 homologues were identified by running the BLASTP program in the phytozome gbrowser (https://phytozome.jgi.doe.gov/pz/portal.html) for GmPGL1. GmPGL1 amino acid and its homologues were aligned with the ClustalW2 (version 2.0.8). Evolutionary relationships were deduced using the neighbor-joining algorithm (Saitou and Nei, 1987). Bootstrapping was performed using the PHYLIP program (version 3.6.7) with 1,000 replicates. The unrooted phylogenetic tree was constructed using the MEGA7 phylogenetic program (Kumar et al., 2016). Protein motifs of *GmPGL1*-like genes are profiled by MEME (version 5.0.5, Seattle, WA, USA).

### Analysis of *GmPGL1*-GFP Subcellular Localization

The full-length CDS of the *GmPGL1* gene is amplified using primers OL8155 and OL8156 that carry *Nde*I and *Sac*I endonuclease restriction sites at their 5'-ends, respectively. The *GmPGL1* CDS was cloned into the modified *pUC19*-*2x35S*-*GFP* vector carrying the *Nde*I and *Sac*I sites (Wang et al., 2015) and a recombinant plasmid carrying the 2×35S: GFP-*GmPGL1* fusion gene was obtained. The 2×35S: GFP-*GmPGL1* plasmid was transiently introduced into *Arabidopsis* (Col-0) mesophyll protoplasts (Gao et al., 2017). The GFP signals were observed using a Nikon confocal microscope C2 (Japan).

### Thiamin, Pigment, Amino Acids, Oxaloacetic Acid, Pyruvate, and α-Ketoglutarate Contents Determination

The thiamine was determined by HPLC as described earlier (Dong et al., 2015). The level of pigment contents of the first true leaves were harvested from Williams 82 and *Gmpgl1*, their 8-day-old seedlings were analyzed using a spectrophotometer as described earlier (Arnon, 1949). Contents of amino acids from 8-day-old leaves were determined using a previously described method (Schwarz et al., 2005; Gheshlaghi et al., 2008). The oxaloacetic acid, pyruvate and α-ketoglutarate from the leaves were extracted using a commercial chemical assay service from Jiangsu Comin Biotechnology Institute (Suzhou, China) according a previously described method (Petrarulo et al., 1990). Three biological replications were examined for both wild type and mutant plants for all above measurements.

### Pyruvate Dehydrogenase, α-Ketoglutarate Dehydrogenase, and Pyruvate Decarboxylase Activity Assay

PDH, α-KGDH and PDC of leaves were determined by a commercial chemical assay service from Jiangsu Comin Biotechnology Institute (Suzhou, China) according the previous described method (Park et al., 2000; Sterk et al., 2003; Kikuchi et al., 2014; Eram and Ma, 2016). The activity assays were repeated from three biological replications.

### Transmission Electron Microscopy

Transmission electron microscopy (TEM) was performed according to a previously described method (Kwon and Cho, 2008). Unifoliate leaves of the 8-day-old Williams 82 and *Gmpgl1* seedlings were observed under a transmission electron microscopy (Hitachi, H-7650) at an accelerating voltage of 100 kV. Ten samples were investigated for both wild type and mutant plants.

## Results

### Isolation and Phenotypic Characterization of the *Gmpgl1* Mutant

**Figure 1:**
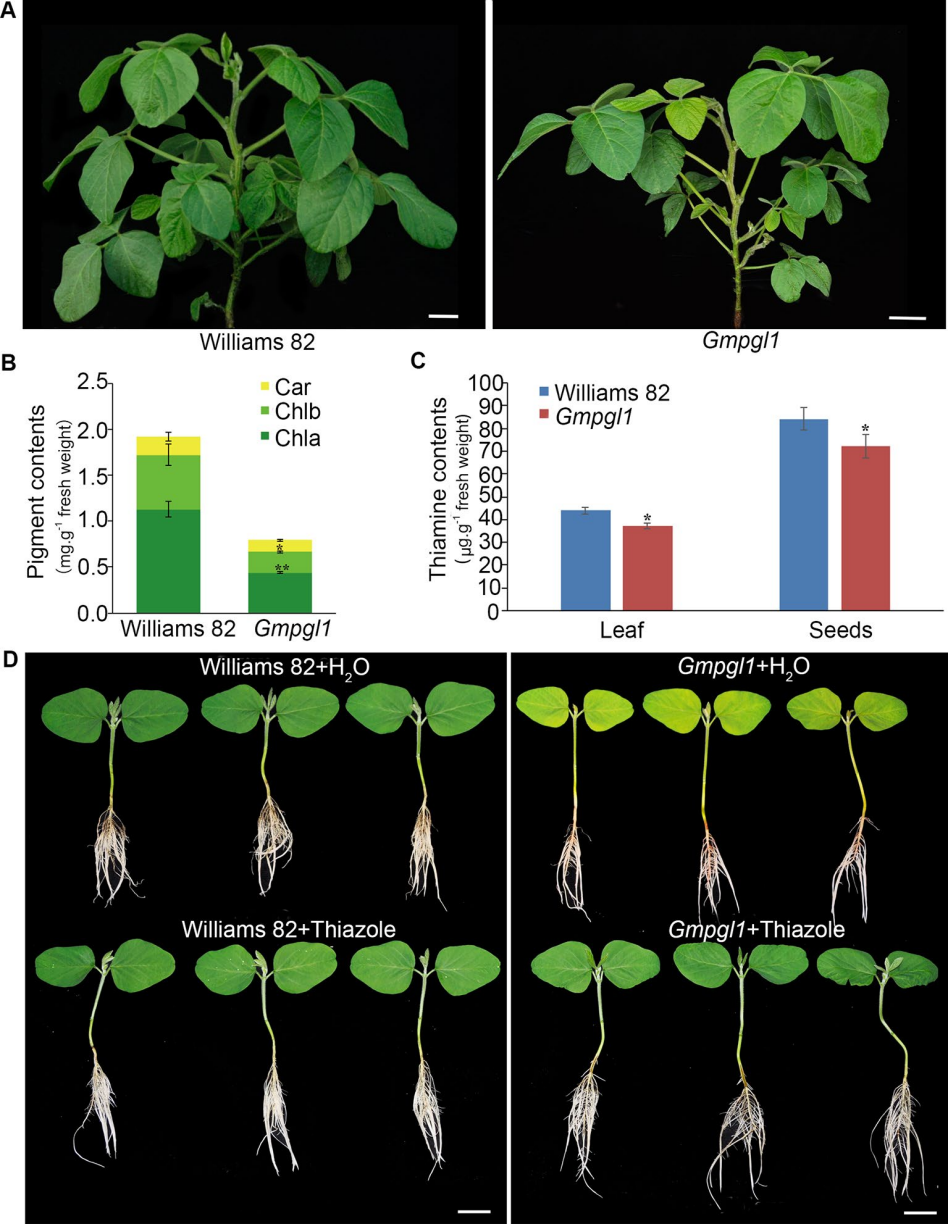
**(A)** The phenotypes of Williams 82 and *Gmpgl1* mutant plant. Scale bars, 4 cm. **(B)** Pigment contents in the unifoliate leaves of 8-day old Williams 82 and *Gmpgl1* seedlings. Chla, chlorophyll a; Chlb, chlorophyll b; Car, carotenoids. **(C)** Thiamine contents in unifoliate leaves of the 8-day-old seedlings, and mature seeds. Values are means from three biological replications. Asterisks indicate a stastically significant difference between the data of Williams 82 and Gmpgl1 determined by student's t-test (**P < 0.01, *P < 0.05) and the error bars represent standard deviations. **(D)** The 8-day old seedlings of Williams 82 and Gmpgl1 supplemented with water and 15 mM thiazole. Scale bars, 3 cm.

To investigate the genetic mechanisms affecting thiamine accumulation in soybean, *Glycine* *max* *pale* *green* *leaf1* (*Gmpgl1*) mutant was isolated from an M_2_ population induced by EMS. The pale green leaf phenotype of *Gmpgl1* mutant appeared yellower in the early development stage of first true leaves, as the leaf developed, the defective phenotype gradually disappeared, and phenotype appeared in the early development of ternately compound leaf (**Figure 1A**). The content of chlorophyll and carotenoid were determined in the Williams 82 and *Gmpgl1* mutant because the content of pigment is associated with changes in leaf color. The total content of chlorophyll, chlorophyll a, chlorophyll b and carotenoid of the *Gmpgl1* mutant was 0.67 ± 0.01, 0.44 ± 0.01 and 0.22 ± 0.01 mg.g^-1^ fresh weight, respectively. While these values of Williams 82 are 1.72 ± 0.12, 1.13 ± 0.08, and 0.59 ± 0.12 mg.g^-1^ fresh weight respectively. These indicated that the pigment contents of *Gmpgl1* mutant are much less than those of wild type (**Figure 1B**).

To determine if *Gmpgl1* mutant influences thiamine accumulation, we quantified the total thiamine content of leaves of 8-day-old seedlings and mature seeds in the Williams 82 and *Gmpgl1* mutant. The content of thiamine in unifoliate leaves of the 8-day-old *Gmpgl1* mutant seedlings was only 74.6% of the corresponding value in the Williams 82 unifoliate leaves, and the content of thiamine in mature seeds decreased by 9.12% compared with that in the Williams 82 (**Figure 1C**).

Feeding of *Gmpgl1* mutant and wild type seedlings with thiazole (15mM) restored the normal green unifoliate leaf phenotype in the *Gmpgl1* mutant (**Figure 1D**). The complementation of the pale green unifoliate phenotype of the *Gmpgl1* with thiazole confirmed that *Gmpgl1* is a thiazole-deficient mutant.

### Genetic Mapping of the *Gmpgl1* Mutation Locus from F_2_ Population

**Figure 2:**
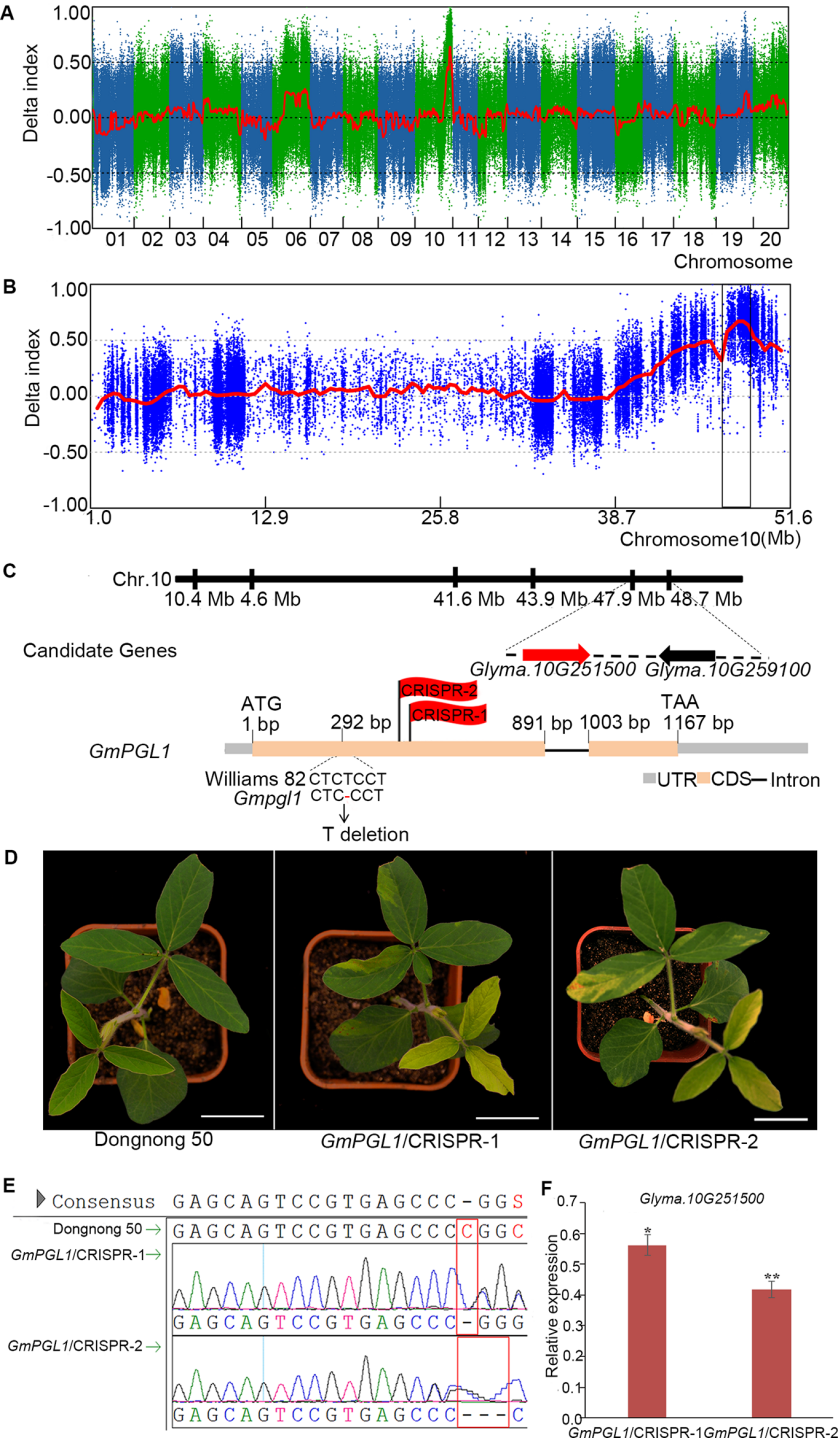
Bulked segregant analysis (BSA) mapping of *Gmpgl1*. **(A)** SNP index plot on all chromosomes of F_2_. **(B)** SNP index plots on chromosomes 10 for *Gmpgl1* mutant from F_2_ population. The interval between 46.90 - 48.90 Mb of chromosome 10 with a highest peak is the candidate region for *Gmpgl1*, in which the ΔSNP-index was greater than mean value (0.5). **(C)** The candidate gene *GmPGL1* (*Glyma.10G251500.1*) is shown in the red arrow, the others in the black arrows. ATG and TAA are the start and stop codons, respectively. One thymidine nucleotide is deleted at 292 bp in the first exon of *GmPGL1* gene. **(D)** Knockout of *GmPGL1* in wild-type background phenocopied the *Gmpgl1* mutant. Scale bars, 3 cm. **(E)** The sequences comparison of CRISPR plants and Dongnong 50 (WT) in the knock down regions. **(F)** The relative expression of *GmPGL1* gene in CRISPR plants compared with Dongnong 50. Asterisks indicate a statistically significant difference determined by student's t-test (**P < 0.01, *P < 0.05) and the error bars represent standard deviations.

The *Gmpgl1* mutant was crossed to Hedou12 to generate a segregation population for mapping the *GmPGL1* gene. The F_1_ plants were normal, but a 3:1 (WT: mutant = 108:32, χ^2^ test, *p* = 0.67) segregation ratio was observed in the F_2_ generation, indicating that the *Gmpgl1* mutation is single and recessive. Based on the phenotype of *Gmpgl1*, the DNA from 32 F_2_ individuals carrying the *GmPGL1* mutation in homozygous condition and 50 F_2_ individuals of wild-type phenotype were pooled into a *Gmpgl1* bulk and a Williams 82 bulk for further bulked segregant analysis (BSA) analysis, respectively. From this sequence comparison, 1,208,350 SNPs and InDels were identified that distinguish the mutant bulk and wild type-bulk genotypes after filtering from 1,434,460 SNPs and InDels. One major statistically significant peak with the highest △ (SNP-index) value was identified in the intervals of 46.90-48.90 Mb region of the Chromosome 10 and was considered as the *GmPGL1* region (**Figures 2A, B**). In this region, three SNPs and two InDels were identified from 3,250 SNPs and 814 InDels related to mutation locus (**Table 1**, **Table S2**). Among them, the C to T transversion of *Glyma.10G259100* at 48,503,156 bp resulted in Asp to Asn conversion in its protein of this gene, and a T-deletion at 47,970,082 bp of *Glyma.10G251500* caused its ORF frameshift; the rest three mutations were not in the coding region. The T-deletion of *Glyma.10G251500.1* was at 292 nucleotide position of its CDS, which resulted in a truncated protein with a premature stop codon at the 357th nucleotide position (**Figure 2C**).

**Table 1:** Three SNPs and two InDels in the candidate region between 46.90 Mb and 48.90 Mb on chromosome 10 in *Gmpgl1* mutant.

Gene	Postiton in Chromosome 10	Mutation	Type	Function Type	Function
*Glyma.10G242600*	47111556	C→T	intergenic	/	Encodes ATP sulfurylase
*Glyma.10G247200*	47561624	C→T	UTR5	/	DUF1677 family protein
*Glyma.10G251500*	47970081	CT→T	Exonic	frameshift	Thiamine thiazile synthase
*Glyma.10G259100*	48503156	C→T	exonic	nonsynonymous	Oxidoreductase, 2OG-Fe (II) oxygenae family protein
*Glyma.10G259300*	48523259	TA→T	integenic	/	RING-H2 finger protein

We compared the expression level of two candidate genes in the leaves of wild-type and *Gmpgl1* mutant. The relative expression of *Glyma.10G251500* is 2.57 ± 0.52 in *Gmpgl1* mutant, and 13.21 ± 2.23 in Williams 82. However, the expression of *Glyma.10G259100* is 0.19 ± 0.018 in *Gmpgl1* mutant and 0.22 ± 0.04 in Williams 82 (**Figure S1**). The expression of the former is much lower in *Gmpgl1* mutant compared with wild-type, while the latter is no significant difference. This indicated that *Glyma.10G251500* might be more likely linked to mutation phenotype. And the independent mutation screening of *Glyma.10G259100* mutation from our lab also proved its mutation could not bring the pale green yellow phenotype.

To confirm whether *Glyma.10G251500.1* was the *GmPGL1* gene, loss-of-function T1 heterozygosis transgenic lines were generated by inducing mutations in the *Glyma.10G251500.1* gene using a CRISPR/Cas9 system. The CRISPR/Cas9-induced mutations in *Glyma.10G251500.1* in two independent mutants caused development of the *Gmpgl1*-specific mutant phenotype (**Figures 2D, E**), the expression level of *Glyma.10G251500.1* decreased in *GmPGL1*/CRISPR-1 and *GmPGL1*/CRISPR-2 (**Figure 2F**), and supplying thiazole could reduce the CRISPR mutants' phenotype to wild type (**Figure S2**). Above results suggested that *Glyma.10G251500.1* is the *GmPGL1* gene.

### *GmPGL1* Encoded Thiamine Thiazole Synthase

**Figure 3:**
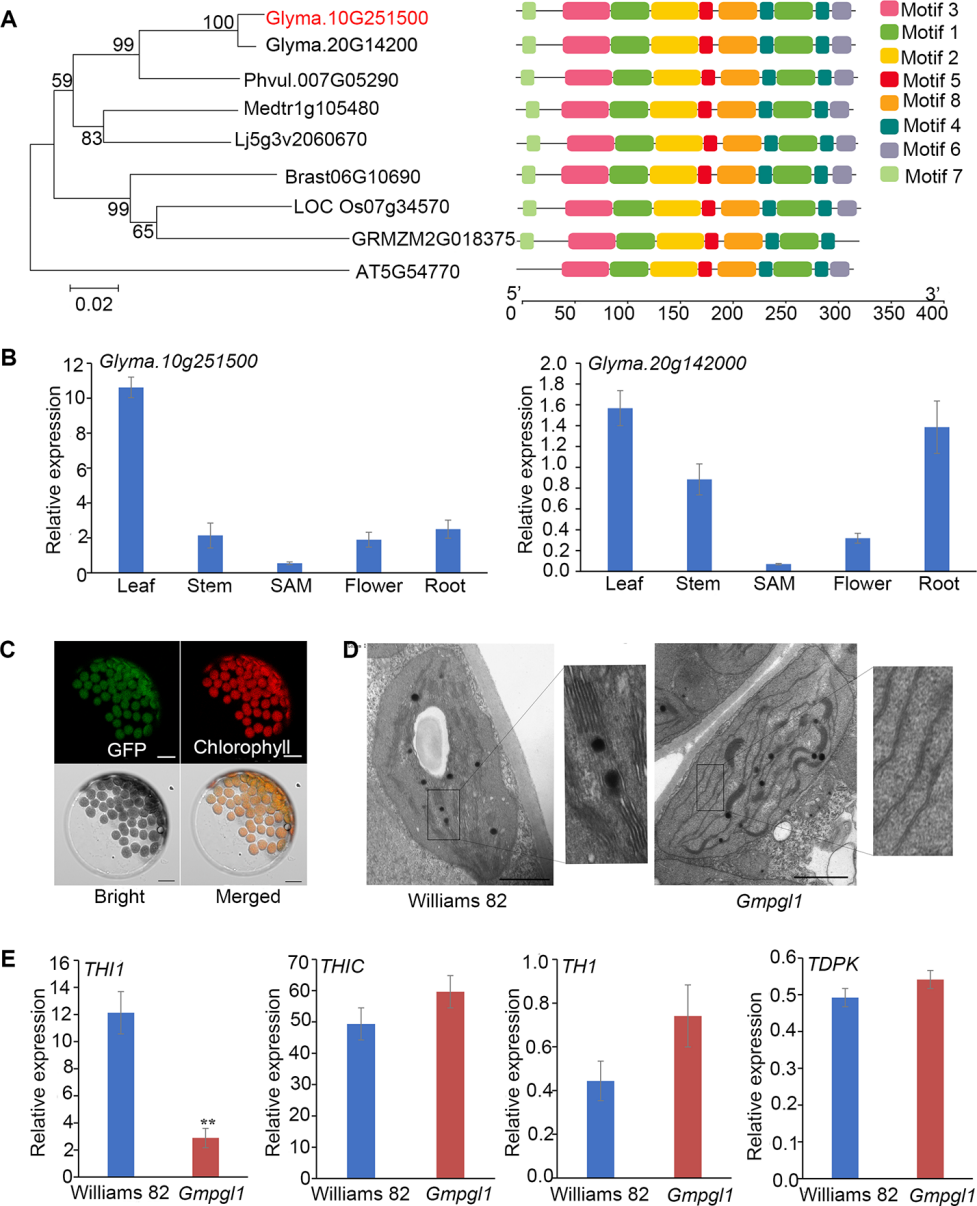
**(A)** Phylogenetic tree of GmPGL1 and its homologous proteins from *Phaseolus vulgaris, Arabidopsis thaliana, Medicago, Lotus corniculatus, Oryza.sativa L, Zea mays L* and *Brassica napus L*. The 8 conserved motifs are represented by colored boxes. The scale of protein length is given below the schematic diagram. **(B)** Expression levels of *GmPGL1* and *Glyma.20G142000.1* gene in different tissues of wild-type Williams 82 plant. **(C)** Transient expresison of GFP-GmPGL1 fusion proteins in Arabidopsis protoplasts. GFP, fluorescence signals of the fusion proteins; Chlorophyll, autofluorescence signals of chloroplasts; Bright, the protoplasts under the bright field; Merged, Merged images of the GFP-GmPGL1 fusion protein, chlorophyll, and bright field images. Scale bars, 10 μm. **(D)** Transmission electron micrographs of chloroplasts from the 8-day old leaves of Williams 82 and Gmpgl1 mutant. Scale bars, 10 μm. **(E)** The relative expression levels of the genes involved in thiamine biosynthesis between the Williams 82 and Gmpgl1 mutant unifoliate leaves. The reference gene is *Cons4* of soybean, values from three independent biological replicates. Asterisks indicate a stastically significant difference between the data of Williams 82 and Gmpgl1 determined by Student's t-test (**P < 0.01) and the error bars represent standard deviations.

Blast searches revealed that GmPGL1 was highly homologous to the *Arabidopsis* THI1 (AT5G54770) protein (69.5% identical amino acids), which encodes a HEP-P synthase. In soybean, the most homologous gene of *GmPGL1* is *Glyma.20G142000.1*, which exhibits 98.3% high-sequence identity with GmPGL1, and 67.5% with *Arabidopsis* THI1. Both of their proteins are 351 amino acid in length. Amino acid sequence comparison generated a phylogenetic tree with *Glycine max* THI1 family and its homologs from dicot plant species and monocotyledon plant species, *Phaseolus vulgaris*, *Arabidopsis thaliana*, *Medicago*, *Lotus corniculatus*, *Oryza.sativa L*, *Zea mays L*, and *Brassica napus L* (**Figure 3A**). It suggests that two THI1 homologues are evolutionarily conserved among plant kingdom, which share the common genomic structure in observed plant species.

We further examined the expression patterns of the *GmPGL1* and its homologue *Glyma.20G142000*. *GmPGL1* expressed higher than *Glyma.20G142000* in all tested tissues, and *GmPGL1* also especially highly expressed in leaves compared that in stem, root and flower (**Figure 3B**). This result suggests that *GmPGL1* might be dominant functional paralogue in soybean.

In the early development of leaves, the relative expression level of *GmPGL1* reduced in *Gmpgl1* mutant compared with Williams 82, however, the relative expression level in *Glyma.20G142000* increased in *Gmpgl1* mutant (**Figure S1**). It suggested the expression of *GmPGL1* reduction caused the lowing of thiamine contents in *Gmpgl1* mutant, the elevated *Glyma.20G142000* expression might compensate for the loss of function of the former.

### *GmPGL1* Localized in Chloroplasts and Influenced Chloroplast Development

It has been reported that THI1 protein had two distinct functions due to different locations in cells (Chabregas et al., 2003; Ajjawi et al., 2007b). Targeting to chloroplasts, THI1 protein is involved in thiamine biosynthesis; while in mitochondria, it is involved in protecting DNA damages in *Arabidopsis*. GmPGL1 is predicted to locate in the chloroplast with TargetP program (http://www.cbs.dtu.dk/services/TargetP/). Transient expression of the *GFP-GmPGL1* fusion gene revealed localization of the *GFP-GmPGL1* fusion protein with the auto-fluorescent signals of chlorophyll (**Figure 3C**). This result indicated that GmPGL1 was located in the chloroplasts.

In order to make sure whether *GmPGL1* influences chloroplast development, TEM analysis was performed. The chloroplasts of Williams 82 cells have abundant and well-ordered thylakoids and stacked membranes, whereas chloroplasts in the *Gmpgl1* mutant had fewer lamellae per chloroplast and fewer lamellae per granum (**Figure 3D**). Because of the complementation of the *Gmpgl1* mutant phenotype with exogenous application of thiazole, it was most likely due to thiamin deficiency but not a direct effect of *GmPGL1* that led to the reduction in chlorophyll content and abnormal chloroplast development in Gmpgl1 mutant.

To resolve the function of GmPGL1 in thiamine biosynthesis pathway, we examined the expression differences of key genes of the thiamine biosynthetic pathway between wild type and *Gmpgl1* mutant including *THI1* (*GmPGL1, Glyma.10g251500*), *THIC* (*Glyma.18g065700*), *TH1* (*Glyma.08g18200*) and *TDPK* (*Glyma.03g213500*). The relative expression levels of *THI1, THIC, TH1 and TPK1* comparisons with *Cons4* are 2.89 ± 0.71, 59.65 ± 10.38, 0.74 ± 0.14, 0.54 ± 0.03 in *Gmpgl1* mutant respectively, while these values of Williams 82 are 12.13 ± 1.56, 49.37 ± 6.89, 0.44 ± 0.09 and 0.49 ± 0.06 respectively. The expression of *THI1* (*GmPGL1*) was down-regulated in the *Gmpgl1* mutant as compared to Williams 82, while the other genes were slightly up-regulated (**Figure 3E**).

### Mutation in *GmPGL1* Led to Altered Primary Metabolism

**Figure 4:**
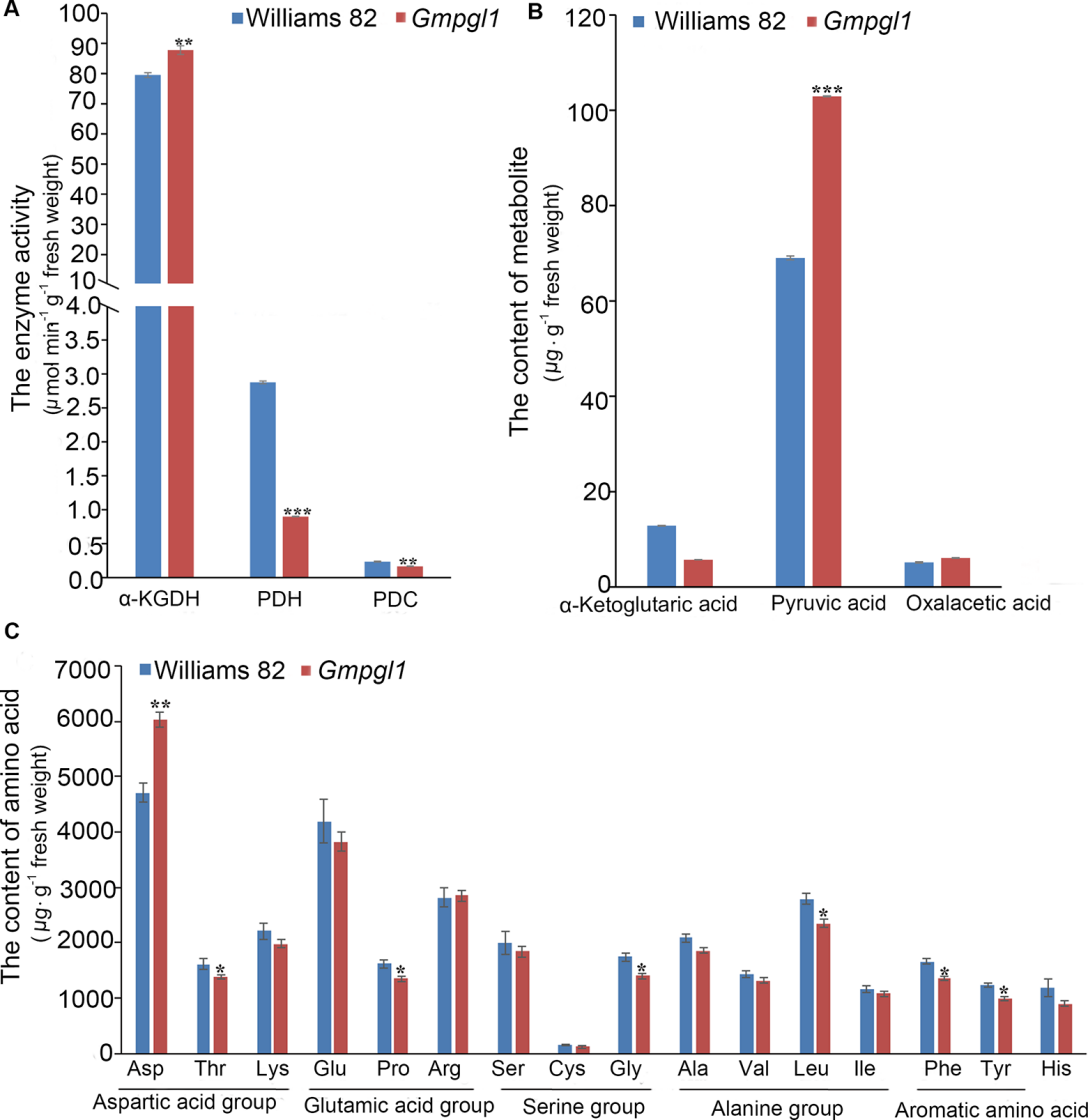
Some enzymes and metabolites changes between Williams 82 and *Gmpgl1* mutant in the unifoliate leaves of 8-day-old seedlings. **(A)** The enzyme activities of α-KGDH, PDH and PDC. **(B)** The contents of α-ketoglutaric, pyruvic acid and oxalacetic acid. **(C)** The contents of 16 amino acid. Values are means ± SD of three replicates, asterisks indicate the statistically significant differences between the Williams 82 and Gmpgl1 mutant. Student's t-test: ***P ρ < 0.001, **P ρ <0.01, *P ρ < 0.05.

Thiamine is a co-factor in several enzymes in key cellular metabolic pathways, such as, PDC, PDH and α-KGDH. PDC is the first enzyme for all homo-fermentative ethanol pathways, and PDH and α-KGDH are two central enzymes in energy metabolism. The activity of α-KGDH, PDH and PDC were 87.79 ± 1.45, 0.90 ± 0.004 and 0.17 ± 0.004 µmol.min^-1^.g^-1^ fresh weight respectively in *Gmpgl1* mutant, while 79.54 ± 0.81, 2.88 ± 0.02 and 0.23 ± 0.006 µmol.min^-1^.g^-1^ fresh weight in Williams 82 (**Figure 4A**). The activity of α-KGDH increased in the *Gmpgl1* mutant as compared to Williams 82. Whereas, the activities of PDH and PDC were significantly reduced in *Gmpgl1* mutant, as compared to that in Williams 82. The changes of above enzymes' activities in the *Gmpgl1* mutant were consistent with the trends of substrate accumulations, such as the reduction of α-ketoglutaric acid, and increment of pyruvic acid in mutant plant (**Figure 4B**).

To determine if the amino acids metabolic pathways in *Gmpgl1* are altered as compared to Williams 82, we conducted metabolic profiling for the amino acids (**Figure 4C**). The contents of Thr, Pro, Gly, Leu, Phe, and Tyr were decreased in the *Gmpgl1* mutant as compared to that in Williams 82, while only Asp was increased. The changes of amino acid related to the contents of their precursors, such as, α-ketoglutaric acid, increment of pyruvic acid and oxaloacetic acid (**Figure 4B**).

These results show that mutation of *GmPGL1* gene effects the thiamine related metabolism enzymes and some primary metabolisms and their precursors.

## Discussion

*THI1* encoded thiamine thiazole synthase is the essential for thiamine biosynthesis. *Arabidopsis tz* mutants are chlorotic and die early without thiazole or thiamine supplementation (Redei, 1962). In a *tz* mutant line, a point mutation in the *THI1* gene causes an amino acid substitution in a highly conserved residue (Papini-Terzi et al., 2003), suggesting that the *Arabidopsis THI1* gene is essential for thiamine biosynthesis. *OsDR8* encodes HET-P synthase and is involved in the synthesis of the thiazole moiety of thiamine in rice (Belanger et al., 1995). In *Arabidopsis th1*, *thi1* and *py* mutants fail to survive without thiamine supplementation (Redei, 1962; Feenstra, 1964; Li and Redei, 1969); and in maize, *blk1-R* mutant is rescued by exogenous thiamine supplementation (Woodward et al., 2010). The content of thiamine was decreased in leaves of the young seedlings and mature seeds of the *Gmpgl1* mutant. The phenotype of *Gmpgl1* can also rescued by thiazole supplementation in our work (**Figure 1D**). These results indicated that the lost function of THI1 reduced thiamine biosynthesis, and thiamin deficiency caused chloroplast abnormal development and leaf chlorosis.

In the *Gmpgl1* mutant, the T-deletion of *Glyma.10G251500.1* resulted in a truncated protein with a premature stop codon at the 357th nucleotide position. The expression of the *GmPGL1* gene was down-regulated in *Gmpgl1* mutant as compared to Williams 82, which might be due to nonsense-mediated mRNA decay (NMD) pathway (Isshiki et al., 2001). However, the relative expression level in *GmPGL1* paralogue, *Glyma.20G142000*, slightly increased in *Gmpgl1* mutant, which probably compensate for the loss of function of the *Gmpgl1* mutant. The further study of the function of *Glyma.20G142000* will help us to comprehensively understand the thiamine biosynthesis pathway in soybean, and the role of thiamine in both abiotic and biotic stresses, soybean development and metabolism.

TPP is the active cofactor form of thiamine required by various enzymes involved in carbohydrate and amino acid metabolism in plants. In Arabidopsis, thiamin level is regulated by a TPP-responsive riboswitch of the THIC pre-mRNA (Bocobza et al., 2007; Wachter et al., 2007). Increasing TMP level in leaves increased extractable PDH, α-KGDH, and TK activities without changing the protein levels of PDH and TK in riboswitch deﬁciency mutant (Bocobza et al., 2013). However, Hsieh et al. (2017) also reported that transcript levels of the genes encoding TPP-dependent enzymes, such as PDH, α-KGDH, and TK, are up-regulated in the TPP deﬁciency mutant. These controversial results might result from different measurement condition and organism. The photosynthetic and metabolic phenotypes of TPP riboswitch mutants are photoperiod dependent, appropriate TPP levels are required for acclimation to changes in photoperiod (Rosado-Souza et al., 2019). The thiamin-requiring enzymes' activities might regulate by different TPP availabilities: allosteric regulation may be the main control over under low TPP availability, high TPP availability becomes a major point of control of these enzymes during the dark photoperiod (Bocobza et al., 2013). We observed that activities of PDH, PDC decreased in *Gmpgl1* mutant, while that of α-KGDH increased (**Figure 4A**). Because the enzyme activity is measured by fresh weight per gram in the study, the reduction activities of PDH, PDC in *Gmpgl1* mutant might cause by either the protein level of enzyme or the enzyme activity per mole. The difference of alter the difference of altering trend among PDH, PDC and α-KGDH in *Gmpgl1* mutant might due to their distributions in plant cells and the binding ability of ThDP cofactor (Hanson et al., 2018; Joshi et al., 2019).

In summary, *GmPGL1* encodes thiamine thiazole synthase, which is targeted to chloroplasts and essential for thiamine biosynthesis. Identification of *GmPGL1* could improve our understanding of the molecular mechanism for thiamine biosynthesis in soybean.

## Data Availability Statement

All datasets for this study are included in the article/**Supplementary Material**.

## Author Contributions

SY and XZF designed the research. XFF performed the experiments. KT provided bioinformatics analyses. YZ, JL, JM and QW provided the technical assistance. XFF, XZF and SY analyzed the data and wrote the manuscript. All authors declared no conflicting interest on the contents of the manuscript.

## Conflict of Interest

The authors declare that the research was conducted in the absence of any commercial or financial relationships that could be construed as a potential conflict of interest.
