# Supplementary material for: GmPGL1, a Thiamine Thiazole Synthase, Is Required for the Biosynthesis of Thiamine in Soybean
Source: Front Plant Sci. 2019 Nov 22;10:1546. doi: 10.3389/fpls.2019.01546 (PMC6883718; doi:10.3389/fpls.2019.01546)
Supplement: Supplementary file 1 [file DataSheet_1.pdf]

Supplementary materials:

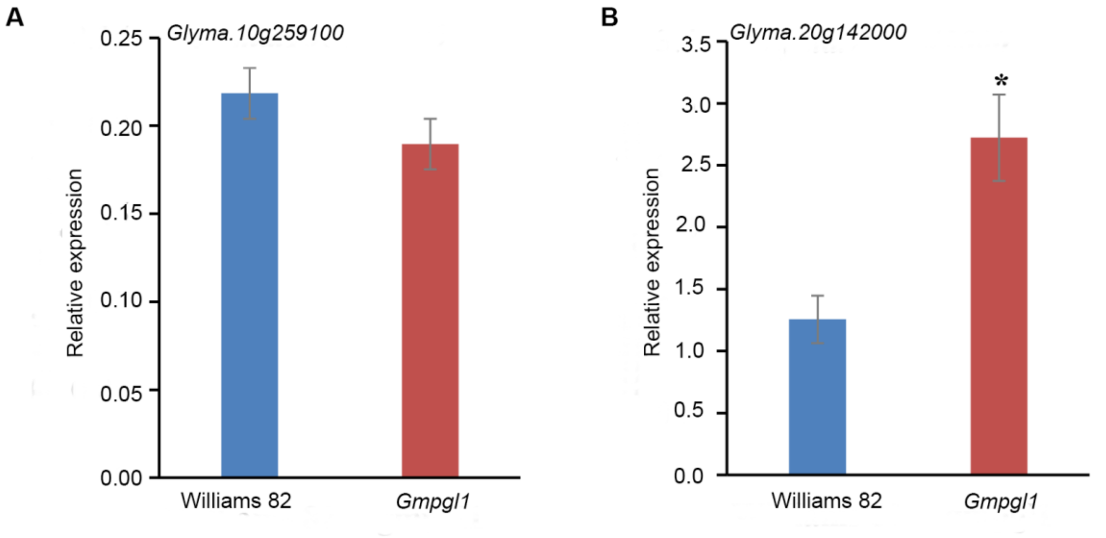

Figure S1 Expression differences of (A) *Glyma.10G259100* and (B) *Glyma.20G142000* between Williams 82 and *Gmpgl1* mutant. The reference gene is *Con4* gene, values from three independent biological replicates. Asterisks indicate a stastically significant difference between the data of Williams 82 and *Gmpgl1* determined by student's *t*-test ($^{*}P$<0.05) and the error bars represent standard deviations.

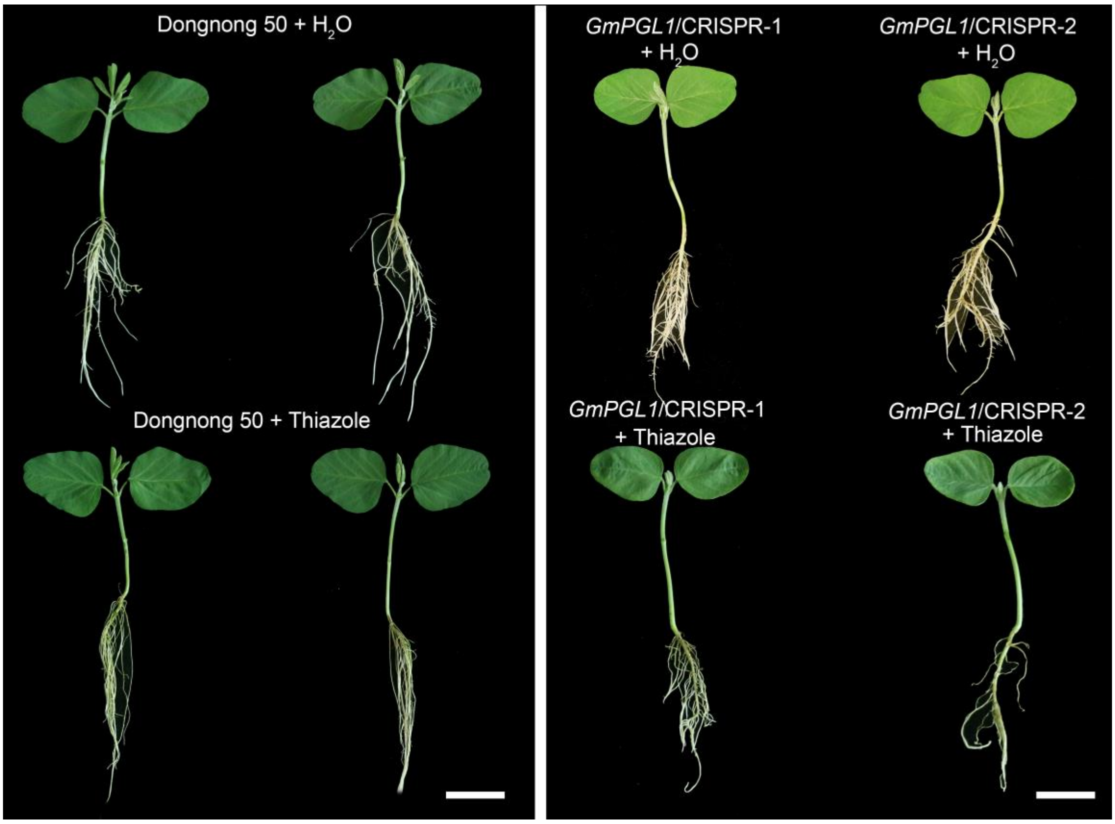

Figure S2 Complementation of the CRISPR plants phenotype with thiazole. The 8-day old seedlings of Dongnong 50, *GmPGL1*/CRISPR-1 and *GmPGL1*/CRISPR-2 supplemented with water and 15 mM thiazole. Scale bars, 3 cm.

TableS1 Primers used in this paper

Primer	Primer sequence 5'-3'
OL8155	CTGTACAAGCATATGATGGCTGCCATGGCAACCACA
OL8156	GATGAATTCGAGCTCTTAAGCATCAACAATTTCCTCAGTG
OL8161	AGTCCCAACAACTATTCTACTTACC
OL8162	CATAATTAAGTAGGGACCAAACAG
OL8321	CCGTGTCTACTGTTCCACTGTCA
OL8322	AACTGGTCTGCCACAAAATCCT
OL8323	GTTCCTCCGAAGCCCAATC
OL8324	AAGGTTCTCCTCGCCCAGT
OL8370	GCTCTGGGACCACGCTCA
OL8371	TGGTGCGAACATGGGAAGG
OL8372	GTCGCACCCACCACCAAGT
OL8373	AGGATTGGAGGTCGTATGGG
OL8374	GGTTTGATGTTGTTGGTCGTGC
OL8375	GTCTGAGTTGGTAGTTGGGGGA
OL8473	CCACTCCGCTCCCCTATCA
OL8474	GAAGAGAGGGTTGTGGTTGCC
OL8475	TCCACTCACCACTGAAACTGAAACA
OL8476	GAAGATGATGATTTGTTGAAGAGGG
OL8477	CCATAACATGGCTTCTTCCACC
OL8478	CGGAGAGTAGGGGTGGCAT
OL9308	CACCCACCACCAAGTTATTCTCA
OL9309	CAACCTTAGCCTCCATCACG

Table S2 SNPs in candidate region

TableS2 SNPs in candidate region

Chromosome	Start	End	Reference	Mutation	Variation type	Mutant bulk genotype	WT bulk genotype	Mutant bulk read depth	WT bulk read depth
Chr10	46902903	46902903	G	C	SNP	0|0	0|1	58|0	6|6
Chr10	46903557	46903557	A	T	SNP	0|0	0|1	58|1	2|13
Chr10	46906121	46906121	G	C	SNP	0|0	0|1	62|0	3|11
Chr10	46911587	46911587	C	T	SNP	0|0	0|1	38|1	8|8
Chr10	46912036	46912036	T	C	SNP	0|0	0|1	26|0	6|5
Chr10	46916578	46916578	T	C	SNP	0|0	0|1	38|0	6|16
Chr10	46920246	46920246	A	G	SNP	0|1	0|1	16|3	5|3
Chr10	46940169	46940169	G	C	SNP	0|0	0|1	38|1	2|19
Chr10	46944668	46944668	C	T	SNP	0|0	0|1	30|0	1|9
Chr10	46948970	46948970	T	G	SNP	0|0	0|1	44|1	8|8
Chr10	46952446	46952446	T	C	SNP	0|0	0|1	11|0	1|14
Chr10	46952667	46952667	G	T	SNP	0|0	0|1	23|0	4|4
Chr10	46953277	46953277	A	G	SNP	0|0	0|1	23|2	4|8
Chr10	46953606	46953606	A	G	SNP	0|0	0|1	20|0	5|3
Chr10	46955858	46955858	T	C	SNP	0|1	0|1	31|3	6|9
Chr10	46957168	46957168	A	G	SNP	0|0	0|1	27|1	4|7
Chr10	46960908	46960908	T	G	SNP	0|1	0|1	7|4	7|1
Chr10	46969352	46969352	A	G	SNP	0|0	0|1	36|0	4|11
Chr10	46969393	46969393	G	A	SNP	0|0	0|1	35|0	3|11
Chr10	46969430	46969430	T	G	SNP	0|0	0|1	37|0	2|7
Chr10	46970086	46970086	T	C	SNP	0|0	0|1	47|1	6|9
Chr10	46970134	46970134	G	T	SNP	0|0	0|1	60|2	7|9
Chr10	46970202	46970202	A	G	SNP	0|0	0|1	59|2	6|12
Chr10	46970581	46970581	G	A	SNP	0|0	0|1	42|2	4|13
Chr10	46970620	46970620	A	C	SNP	0|0	0|1	35|1	4|13
Chr10	46970707	46970707	T	G	SNP	0|0	0|1	11|0	2|6
Chr10	46971259	46971259	G	A	SNP	0|0	0|1	39|1	4|4
Chr10	46971360	46971360	G	A	SNP	0|1	0|1	41|4	12|6
Chr10	46971477	46971477	T	C	SNP	0|0	0|1	40|1	8|7
Chr10	46972041	46972041	T	G	SNP	0|0	0|1	58|1	7|22
Chr10	46972222	46972222	G	A	SNP	0|0	0|1	31|0	6|10
Chr10	46972309	46972309	T	C	SNP	0|0	0|1	26|0	3|9
Chr10	46974377	46974377	T	C	SNP	0|1	1|1	17|3	0|8
Chr10	46974470	46974470	A	C	SNP	0|1	0|1	16|3	4|4
Chr10	46974514	46974514	C	T	SNP	0|0	0|1	20|1	4|8
Chr10	46975782	46975782	G	T	SNP	0|0	0|1	28|1	5|10
Chr10	46976047	46976047	T	C	SNP	0|0	0|1	62|3	3|5

Chr10	46976793	46976793	T	A	SNP	0|0	0|1	31|0	2|8
Chr10	46977915	46977915	A	T	SNP	0|0	0|1	24|1	5|3
Chr10	46982896	46982896	G	A	SNP	0|0	0|1	33|1	1|12
Chr10	46983255	46983255	T	C	SNP	0|0	0|1	24|0	3|9
Chr10	46983295	46983295	C	T	SNP	0|0	0|1	33|0	4|13
Chr10	46991481	46991481	G	A	SNP	0|1	0|1	24|12	5|5
Chr10	46991488	46991488	C	T	SNP	0|1	0|1	27|12	5|5
Chr10	46991492	46991492	G	A	SNP	0|1	0|1	27|11	6|5
Chr10	46991517	46991517	T	A	SNP	0|1	0|1	24|12	8|4
Chr10	46991563	46991563	T	G	SNP	0|1	0|1	14|7	8|3
Chr10	46991567	46991567	T	G	SNP	0|1	0|1	14|6	8|3
Chr10	46991573	46991573	G	C	SNP	0|1	0|1	13|6	8|3
Chr10	46991574	46991574	G	A	SNP	0|1	0|1	13|6	8|3
Chr10	46992139	46992139	C	T	SNP	0|1	0|0	2|8	8|0
Chr10	46992527	46992527	A	G	SNP	0|0	0|1	18|1	4|5
Chr10	46992555	46992555	C	T	SNP	0|0	0|1	21|0	3|5
Chr10	46992637	46992637	G	A	SNP	0|0	0|1	17|1	3|7
Chr10	46993062	46993062	C	T	SNP	0|1	0|1	16|3	5|3
Chr10	46993066	46993066	A	G	SNP	0|1	0|1	17|5	6|3
Chr10	46993071	46993071	C	T	SNP	0|1	0|1	16|8	6|3
Chr10	46993080	46993080	A	G	SNP	0|1	0|1	17|8	6|4
Chr10	46993112	46993112	T	A	SNP	0|1	0|1	22|24	8|8
Chr10	46993115	46993115	C	G	SNP	0|1	0|1	22|24	8|8
Chr10	46993120	46993120	T	C	SNP	0|1	0|1	22|24	9|8
Chr10	46993124	46993124	T	C	SNP	0|1	0|1	27|25	9|8
Chr10	46993130	46993130	T	A	SNP	0|1	0|1	27|27	8|8
Chr10	46993132	46993132	T	G	SNP	0|1	0|1	27|27	8|8
Chr10	46993176	46993176	T	A	SNP	0|1	0|1	27|34	13|11
Chr10	46993340	46993340	G	C	SNP	0|1	0|1	62|7	12|6
Chr10	46993341	46993341	T	A	SNP	0|1	0|1	62|7	12|6
Chr10	46993344	46993344	G	A	SNP	0|1	0|1	63|7	12|6
Chr10	46996328	46996328	C	T	SNP	0|0	0|1	44|2	9|15
Chr10	46996512	46996512	C	T	SNP	0|1	0|1	37|3	10|9
Chr10	46996533	46996533	A	C	SNP	0|0	0|1	45|3	8|11
Chr10	46998332	46998332	T	G	SNP	0|0	0|1	19|0	6|3
Chr10	46999153	46999153	A	G	SNP	0|0	0|1	31|0	8|13
Chr10	46999442	46999442	G	A	SNP	0|0	0|1	53|1	5|12
Chr10	46999527	46999527	T	A	SNP	0|0	0|1	38|0	2|10
Chr10	46999906	46999906	G	A	SNP	0|0	0|1	29|1	5|3
Chr10	47000002	47000002	T	C	SNP	0|0	0|1	33|1	6|4
Chr10	47000062	47000062	A	G	SNP	0|0	0|1	38|1	6|7
Chr10	47000271	47000271	T	A	SNP	0|0	0|1	67|3	7|4

Chr10	47000400	47000400	G	T	SNP	0|0	0|1	51|4	7|3
Chr10	47000539	47000539	G	A	SNP	0|0	0|1	62|2	4|7
Chr10	47000565	47000565	T	C	SNP	0|0	0|1	56|2	4|7
Chr10	47000566	47000566	A	G	SNP	0|0	0|1	56|2	4|7
Chr10	47001280	47001280	G	A	SNP	0|0	0|1	15|0	2|7
Chr10	47001845	47001845	T	C	SNP	0|0	0|1	15|0	3|5
Chr10	47001890	47001890	A	T	SNP	0|0	0|1	21|0	4|5
Chr10	47003374	47003374	G	A	SNP	0|0	0|1	57|2	6|19
Chr10	47004735	47004735	A	G	SNP	0|0	0|1	50|3	7|13
Chr10	47005020	47005020	T	C	SNP	0|0	0|1	40|3	4|15
Chr10	47005705	47005705	G	A	SNP	0|1	0|1	38|3	6|6
Chr10	47005886	47005886	T	C	SNP	0|0	0|1	36|0	4|4
Chr10	47006032	47006032	G	A	SNP	0|0	0|1	48|0	7|4
Chr10	47006221	47006221	A	G	SNP	0|0	0|1	36|0	8|10
Chr10	47006267	47006267	A	G	SNP	0|0	0|1	31|0	10|10
Chr10	47006304	47006304	A	G	SNP	0|0	0|1	23|0	7|7
Chr10	47006335	47006335	C	T	SNP	0|0	0|1	15|0	5|5
Chr10	47007600	47007600	G	A	SNP	0|0	0|1	23|0	1|10
Chr10	47007625	47007625	G	A	SNP	0|0	1|1	25|0	0|9
Chr10	47007628	47007628	A	C	SNP	0|0	1|1	25|0	0|9
Chr10	47007638	47007638	C	A	SNP	0|0	1|1	28|0	0|9
Chr10	47007668	47007668	T	G	SNP	0|0	1|1	21|0	0|9
Chr10	47007962	47007962	C	G	SNP	0|0	0|1	47|0	2|12
Chr10	47008187	47008187	C	A	SNP	0|0	0|1	64|0	7|23
Chr10	47008268	47008268	A	T	SNP	0|0	0|1	41|1	8|21
Chr10	47008781	47008781	G	A	SNP	0|0	0|1	51|0	5|9
Chr10	47008919	47008919	T	G	SNP	0|0	0|1	69|3	6|14
Chr10	47008962	47008962	G	T	SNP	0|0	0|1	86|3	7|14
Chr10	47010363	47010363	G	A	SNP	0|0	0|1	86|1	12|8
Chr10	47010526	47010526	A	G	SNP	0|1	0|1	55|5	4|16
Chr10	47010661	47010661	G	A	SNP	0|1	0|1	72|8	4|7
Chr10	47012735	47012735	A	G	SNP	0|0	0|1	27|1	3|5
Chr10	47012740	47012740	T	C	SNP	0|0	0|1	24|1	3|6
Chr10	47012791	47012791	G	A	SNP	0|0	0|1	27|1	3|6
Chr10	47012818	47012818	C	T	SNP	0|0	0|1	29|1	4|8
Chr10	47012876	47012876	T	A	SNP	0|0	0|1	37|1	6|9
Chr10	47013224	47013224	G	T	SNP	0|0	0|1	49|0	8|10
Chr10	47013278	47013278	T	C	SNP	0|0	0|1	51|0	7|12
Chr10	47020407	47020407	C	T	SNP	0|0	0|1	38|0	3|5
Chr10	47033722	47033722	G	T	SNP	0|0	0|1	59|2	7|10
Chr10	47034550	47034550	G	A	SNP	0|1	0|1	37|3	3|12
Chr10	47036249	47036249	A	G	SNP	0|0	0|1	16|0	2|6

Chr10	47037254	47037254	T	C	SNP	0|0	0|1	19|1	4|5
Chr10	47037536	47037536	A	G	SNP	0|0	0|1	36|2	6|9
Chr10	47038720	47038720	A	G	SNP	0|0	0|1	56|2	6|9
Chr10	47039436	47039436	G	A	SNP	0|0	0|1	48|1	11|11
Chr10	47042136	47042136	C	T	SNP	0|0	0|1	72|3	5|15
Chr10	47045556	47045556	T	A	SNP	0|0	0|1	19|0	6|5
Chr10	47056691	47056691	G	T	SNP	0|0	0|1	18|0	2|6
Chr10	47058010	47058010	G	C	SNP	0|0	0|1	52|0	4|13
Chr10	47058298	47058298	G	A	SNP	0|0	0|1	31|0	1|10
Chr10	47059021	47059021	A	G	SNP	0|0	0|1	18|0	4|8
Chr10	47059268	47059268	A	G	SNP	0|0	0|1	27|1	2|6
Chr10	47059429	47059429	G	A	SNP	0|1	0|1	30|3	2|6
Chr10	47060014	47060014	A	C	SNP	0|0	0|1	32|0	8|16
Chr10	47060018	47060018	C	T	SNP	0|0	0|1	33|0	8|16
Chr10	47060080	47060080	A	T	SNP	0|0	0|1	20|0	4|13
Chr10	47061730	47061730	A	T	SNP	0|0	0|1	36|1	7|10
Chr10	47062012	47062012	A	G	SNP	0|0	0|1	43|1	9|9
Chr10	47063767	47063767	A	G	SNP	0|0	0|1	15|0	4|8
Chr10	47063921	47063921	C	A	SNP	0|0	0|1	30|0	3|26
Chr10	47063969	47063969	A	G	SNP	0|0	0|1	39|0	7|22
Chr10	47065375	47065375	A	G	SNP	0|0	0|1	62|0	7|11
Chr10	47065461	47065461	T	A	SNP	0|0	0|1	36|1	8|13
Chr10	47065948	47065948	T	C	SNP	0|0	0|1	30|0	9|8
Chr10	47066078	47066078	A	T	SNP	0|0	0|1	43|0	6|15
Chr10	47066678	47066678	A	C	SNP	0|0	0|1	33|0	5|12
Chr10	47068432	47068432	T	C	SNP	0|0	0|1	41|1	4|11
Chr10	47068472	47068472	A	T	SNP	0|0	0|1	27|1	5|8
Chr10	47068550	47068550	T	G	SNP	0|0	0|1	27|0	3|6
Chr10	47070107	47070107	C	G	SNP	0|0	0|1	45|0	3|14
Chr10	47070195	47070195	C	A	SNP	0|0	1|1	32|0	0|9
Chr10	47071798	47071798	T	C	SNP	0|0	0|1	31|0	5|6
Chr10	47072761	47072761	G	T	SNP	0|0	0|1	11|0	3|6
Chr10	47075026	47075026	A	T	SNP	0|0	0|1	28|0	1|9
Chr10	47075111	47075111	C	T	SNP	0|0	0|1	38|0	5|7
Chr10	47075532	47075532	T	C	SNP	0|0	0|1	46|0	12|9
Chr10	47075561	47075561	A	T	SNP	0|0	0|1	51|0	10|15
Chr10	47076133	47076133	C	T	SNP	0|0	1|1	11|0	0|10
Chr10	47076296	47076296	G	A	SNP	0|0	0|1	16|0	1|12
Chr10	47076326	47076326	T	A	SNP	0|0	1|1	18|0	0|13
Chr10	47076384	47076384	G	A	SNP	0|0	0|1	24|0	3|10
Chr10	47076480	47076480	T	A	SNP	0|0	0|1	41|0	6|9
Chr10	47076496	47076496	T	G	SNP	0|0	0|1	42|0	6|8

Chr10	47077248	47077248	A	C	SNP	0|0	0|1	23|0	5|9
Chr10	47078235	47078235	C	T	SNP	0|0	0|1	17|1	4|4
Chr10	47078417	47078417	T	C	SNP	0|0	0|1	28|0	4|7
Chr10	47078722	47078722	G	T	SNP	0|0	0|1	37|0	5|9
Chr10	47080440	47080440	T	C	SNP	0|0	0|1	28|2	5|7
Chr10	47081422	47081422	C	T	SNP	0|1	0|1	7|1	3|6
Chr10	47081806	47081806	A	T	SNP	0|0	0|1	64|0	7|15
Chr10	47081938	47081938	C	A	SNP	0|0	0|1	59|1	7|23
Chr10	47082314	47082314	A	C	SNP	0|0	0|1	59|1	2|12
Chr10	47082380	47082380	A	C	SNP	0|0	0|1	46|1	2|13
Chr10	47089584	47089584	G	C	SNP	0|0	0|1	48|0	14|22
Chr10	47089777	47089777	A	C	SNP	0|1	0|1	42|6	10|11
Chr10	47090643	47090643	C	T	SNP	0|0	0|1	22|2	4|14
Chr10	47092673	47092673	A	G	SNP	0|0	0|1	26|0	6|6
Chr10	47092719	47092719	A	G	SNP	0|0	0|1	23|0	4|5
Chr10	47092726	47092726	C	T	SNP	0|0	0|1	25|0	3|5
Chr10	47095041	47095041	G	C	SNP	0|0	0|1	58|1	12|9
Chr10	47095055	47095055	G	A	SNP	0|0	0|1	63|1	9|12
Chr10	47099455	47099455	T	C	SNP	0|0	0|1	92|1	11|16
Chr10	47103794	47103794	G	C	SNP	0|0	0|1	10|0	5|8
Chr10	47111556	47111556	C	T	SNP	1|1	0|1	0|22	13|4
Chr10	47113027	47113027	T	A	SNP	0|1	0|0	8|4	8|0
Chr10	47113092	47113092	A	G	SNP	0|1	0|1	12|6	11|2
Chr10	47154884	47154884	T	G	SNP	0|0	0|1	28|2	8|12
Chr10	47155419	47155419	T	C	SNP	0|0	0|1	50|0	2|6
Chr10	47155671	47155671	G	T	SNP	0|0	0|1	53|0	11|13
Chr10	47155738	47155738	G	A	SNP	0|1	0|1	46|28	18|13
Chr10	47155762	47155762	G	A	SNP	0|1	0|1	51|28	14|14
Chr10	47155773	47155773	C	T	SNP	0|1	0|1	43|29	13|13
Chr10	47155793	47155793	C	T	SNP	0|1	0|1	47|27	14|19
Chr10	47155807	47155807	G	C	SNP	0|1	0|1	43|28	13|20
Chr10	47155814	47155814	T	G	SNP	0|1	0|1	43|31	13|22
Chr10	47155821	47155821	C	T	SNP	0|1	0|1	43|33	12|18
Chr10	47155842	47155842	C	T	SNP	0|1	0|1	41|35	15|19
Chr10	47155856	47155856	C	T	SNP	0|1	0|1	41|38	16|28
Chr10	47155861	47155861	G	C	SNP	0|1	0|1	38|34	16|28
Chr10	47155916	47155916	G	T	SNP	0|1	0|1	40|45	19|28
Chr10	47155922	47155922	A	G	SNP	0|1	0|1	41|49	20|26
Chr10	47155943	47155943	G	C	SNP	0|1	0|1	43|56	19|24
Chr10	47165745	47165745	A	G	SNP	0|0	1|1	21|0	0|9
Chr10	47166711	47166711	A	G	SNP	0|0	0|1	13|0	1|7
Chr10	47166789	47166789	C	T	SNP	0|0	0|1	17|0	4|12

Chr10	47166790	47166790	C	T	SNP	0|0	0|1	18|0	4|12
Chr10	47166847	47166847	C	G	SNP	0|0	0|1	21|0	6|12
Chr10	47166867	47166867	G	A	SNP	0|0	0|1	21|0	5|12
Chr10	47167066	47167066	C	T	SNP	0|0	0|1	48|0	5|4
Chr10	47167089	47167089	A	G	SNP	0|0	0|1	37|0	6|3
Chr10	47167229	47167229	G	A	SNP	0|0	0|1	36|0	3|8
Chr10	47167232	47167232	G	C	SNP	0|0	0|1	40|0	3|8
Chr10	47167403	47167403	T	C	SNP	0|0	0|1	38|1	5|9
Chr10	47167531	47167531	G	T	SNP	0|0	0|1	41|2	4|9
Chr10	47167626	47167626	G	C	SNP	0|0	0|1	28|0	2|11
Chr10	47169012	47169012	G	A	SNP	0|0	0|1	67|0	13|8
Chr10	47169073	47169073	T	A	SNP	0|0	0|1	63|0	7|7
Chr10	47169758	47169758	T	C	SNP	0|0	0|1	30|0	4|12
Chr10	47171383	47171383	T	A	SNP	0|0	0|1	47|1	4|10
Chr10	47171805	47171805	A	C	SNP	0|0	0|1	45|0	1|12
Chr10	47171956	47171956	T	C	SNP	0|0	0|1	48|0	3|10
Chr10	47172517	47172517	G	A	SNP	0|0	0|1	49|0	1|13
Chr10	47172602	47172602	T	A	SNP	0|0	0|1	51|0	2|9
Chr10	47173896	47173896	C	G	SNP	0|0	0|1	22|0	2|10
Chr10	47174782	47174782	A	G	SNP	0|0	0|1	57|3	6|21
Chr10	47177175	47177175	A	G	SNP	0|0	0|1	63|0	6|14
Chr10	47177587	47177587	T	G	SNP	0|0	0|1	59|2	7|11
Chr10	47177869	47177869	T	C	SNP	0|0	0|1	57|1	16|10
Chr10	47178190	47178190	A	G	SNP	0|0	0|1	46|0	7|12
Chr10	47178304	47178304	A	G	SNP	0|0	0|1	44|0	15|7
Chr10	47178732	47178732	G	A	SNP	0|0	0|1	65|4	10|7
Chr10	47179599	47179599	C	T	SNP	0|0	0|1	42|0	5|9
Chr10	47180003	47180003	T	C	SNP	0|0	0|1	44|1	6|7
Chr10	47180310	47180310	A	G	SNP	0|0	0|1	49|0	2|10
Chr10	47180670	47180670	G	A	SNP	0|0	0|1	54|0	2|6
Chr10	47181007	47181007	C	T	SNP	0|0	0|1	24|0	4|11
Chr10	47181915	47181915	C	T	SNP	0|0	0|1	10|0	1|7
Chr10	47181967	47181967	A	G	SNP	0|0	0|1	22|0	4|6
Chr10	47182019	47182019	T	C	SNP	0|0	0|1	17|0	4|7
Chr10	47182739	47182739	T	G	SNP	0|0	0|1	34|0	4|17
Chr10	47183297	47183297	A	G	SNP	0|0	1|1	11|0	0|8
Chr10	47185076	47185076	A	C	SNP	0|0	0|1	43|0	4|10
Chr10	47185420	47185420	A	G	SNP	0|0	0|1	29|0	2|14
Chr10	47186241	47186241	G	C	SNP	0|0	0|1	35|0	3|8
Chr10	47186595	47186595	T	A	SNP	0|0	0|1	53|0	9|22
Chr10	47186597	47186597	T	C	SNP	0|0	0|1	53|0	9|22
Chr10	47187383	47187383	T	C	SNP	0|0	0|1	48|0	4|11

Chr10	47187440	47187440	G	A	SNP	0|0	0|1	36|0	5|4
Chr10	47188283	47188283	T	C	SNP	0|0	0|1	9|0	5|3
Chr10	47188311	47188311	A	C	SNP	0|0	0|1	14|0	5|3
Chr10	47188313	47188313	G	T	SNP	0|0	0|1	14|0	5|3
Chr10	47188868	47188868	C	T	SNP	0|0	0|1	27|1	6|8
Chr10	47188880	47188880	G	A	SNP	0|0	0|1	27|1	5|7
Chr10	47189059	47189059	A	G	SNP	0|0	0|1	33|2	4|14
Chr10	47189106	47189106	C	T	SNP	0|0	0|1	39|1	4|9
Chr10	47189334	47189334	A	G	SNP	0|0	0|1	42|0	3|13
Chr10	47190236	47190236	A	G	SNP	0|0	0|1	41|0	4|5
Chr10	47190541	47190541	A	G	SNP	0|0	0|1	28|2	9|10
Chr10	47190822	47190822	T	C	SNP	0|0	0|1	33|0	4|5
Chr10	47191085	47191085	A	G	SNP	0|0	0|1	47|0	5|7
Chr10	47191124	47191124	G	A	SNP	0|0	0|1	47|0	7|11
Chr10	47191205	47191205	A	G	SNP	0|0	0|1	46|0	8|17
Chr10	47192803	47192803	C	T	SNP	0|0	0|1	18|1	2|6
Chr10	47194294	47194294	A	C	SNP	0|0	0|1	20|0	3|13
Chr10	47195629	47195629	C	A	SNP	0|0	0|1	70|2	4|9
Chr10	47197258	47197258	G	A	SNP	0|0	0|1	65|0	12|12
Chr10	47197588	47197588	T	C	SNP	0|0	0|1	83|1	17|14
Chr10	47197678	47197678	A	C	SNP	0|0	0|1	66|0	8|5
Chr10	47198535	47198535	C	T	SNP	0|0	0|1	54|1	3|13
Chr10	47198760	47198760	A	T	SNP	0|0	0|1	25|0	1|7
Chr10	47198767	47198767	C	G	SNP	0|0	0|1	31|0	1|7
Chr10	47199132	47199132	G	T	SNP	0|0	0|1	64|0	8|10
Chr10	47199417	47199417	C	T	SNP	0|0	0|1	58|0	7|6
Chr10	47200247	47200247	A	T	SNP	0|0	0|1	75|0	10|11
Chr10	47200521	47200521	T	C	SNP	0|0	0|1	46|3	7|18
Chr10	47203477	47203477	C	T	SNP	0|0	0|1	27|0	4|16
Chr10	47207165	47207165	G	A	SNP	0|0	0|1	42|1	5|3
Chr10	47207558	47207558	G	C	SNP	0|0	0|1	55|0	6|11
Chr10	47207847	47207847	C	A	SNP	0|0	0|1	50|0	5|6
Chr10	47208082	47208082	C	T	SNP	0|0	0|1	27|0	3|7
Chr10	47208764	47208764	C	T	SNP	0|0	0|1	39|0	6|11
Chr10	47208927	47208927	A	T	SNP	0|0	0|1	54|4	8|14
Chr10	47209014	47209014	A	G	SNP	0|1	0|1	35|6	7|11
Chr10	47209828	47209828	A	G	SNP	0|0	0|1	37|0	7|13
Chr10	47209861	47209861	C	T	SNP	0|0	0|1	34|0	5|12
Chr10	47209915	47209915	A	G	SNP	0|0	0|1	34|0	4|10
Chr10	47209936	47209936	C	T	SNP	0|0	0|1	26|0	3|14
Chr10	47209957	47209957	A	G	SNP	0|0	0|1	22|0	2|15
Chr10	47210052	47210052	C	A	SNP	0|0	0|1	44|2	10|15

Chr10	47210069	47210069	G	A	SNP	0|0	0|1	46|2	9|15
Chr10	47210562	47210562	T	C	SNP	0|0	0|1	70|0	6|13
Chr10	47210635	47210635	T	C	SNP	0|0	0|1	56|0	6|12
Chr10	47210893	47210893	A	G	SNP	0|0	0|1	51|1	8|16
Chr10	47211259	47211259	A	T	SNP	0|0	0|1	68|0	3|12
Chr10	47211333	47211333	C	T	SNP	0|0	0|1	68|0	8|16
Chr10	47211398	47211398	C	T	SNP	0|0	0|1	52|0	10|13
Chr10	47211423	47211423	A	G	SNP	0|0	0|1	47|0	9|12
Chr10	47211433	47211433	A	C	SNP	0|0	0|1	48|0	6|10
Chr10	47211508	47211508	G	C	SNP	0|0	0|1	53|2	2|7
Chr10	47212007	47212007	T	C	SNP	0|0	0|1	17|0	8|6
Chr10	47212119	47212119	A	C	SNP	0|0	0|1	23|0	3|6
Chr10	47212152	47212152	T	C	SNP	0|0	0|1	20|0	2|7
Chr10	47212504	47212504	A	G	SNP	0|0	0|1	20|0	2|6
Chr10	47212885	47212885	A	G	SNP	0|0	0|1	25|0	2|7
Chr10	47212925	47212925	C	T	SNP	0|0	0|1	21|0	4|9
Chr10	47213658	47213658	A	T	SNP	0|0	0|1	62|0	1|9
Chr10	47213794	47213794	C	T	SNP	0|0	1|1	61|0	0|10
Chr10	47214896	47214896	C	T	SNP	0|0	0|1	46|0	8|10
Chr10	47215982	47215982	G	C	SNP	0|0	0|1	54|1	12|12
Chr10	47219786	47219786	T	G	SNP	0|0	0|1	51|0	13|12
Chr10	47221188	47221188	C	A	SNP	0|0	0|1	27|0	6|8
Chr10	47221297	47221297	C	T	SNP	0|0	0|1	21|0	8|12
Chr10	47222377	47222377	A	C	SNP	0|0	0|1	50|0	9|8
Chr10	47222493	47222493	T	A	SNP	0|0	0|1	45|0	5|6
Chr10	47223116	47223116	G	A	SNP	0|0	0|1	53|0	4|10
Chr10	47223793	47223793	G	A	SNP	0|0	0|1	26|0	4|7
Chr10	47223945	47223945	G	A	SNP	0|0	0|1	26|0	3|11
Chr10	47225871	47225871	A	G	SNP	0|0	0|1	68|0	6|16
Chr10	47226706	47226706	G	T	SNP	0|0	0|1	50|0	7|8
Chr10	47227094	47227094	T	C	SNP	0|0	0|1	62|0	2|17
Chr10	47230664	47230664	A	T	SNP	0|0	0|1	35|1	5|13
Chr10	47230848	47230848	G	T	SNP	0|0	0|1	35|1	7|6
Chr10	47230876	47230876	C	A	SNP	0|0	0|1	35|1	9|6
Chr10	47231760	47231760	A	T	SNP	0|0	1|1	14|0	0|12
Chr10	47231785	47231785	A	G	SNP	0|0	1|1	15|0	0|12
Chr10	47232092	47232092	C	A	SNP	0|0	1|1	31|1	0|10
Chr10	47232119	47232119	C	T	SNP	0|0	1|1	28|1	0|9
Chr10	47232790	47232790	C	A	SNP	0|0	0|1	32|0	8|7
Chr10	47235331	47235331	G	T	SNP	0|0	0|1	49|0	9|10
Chr10	47235379	47235379	A	G	SNP	0|0	0|1	50|0	6|12
Chr10	47235598	47235598	T	A	SNP	0|0	0|1	41|1	2|17

Chr10	47240612	47240612	C	G	SNP	0|0	0|1	31|0	3|9
Chr10	47240636	47240636	T	G	SNP	0|0	0|1	30|0	3|10
Chr10	47240686	47240686	A	G	SNP	0|0	0|1	25|0	3|7
Chr10	47241883	47241883	C	A	SNP	0|0	0|1	23|0	6|5
Chr10	47242495	47242495	A	G	SNP	0|0	0|1	15|0	4|6
Chr10	47242539	47242539	A	T	SNP	0|0	0|1	15|0	7|9
Chr10	47242624	47242624	G	A	SNP	0|0	0|1	23|0	9|11
Chr10	47242659	47242659	T	C	SNP	0|0	0|1	31|0	6|5
Chr10	47242676	47242676	A	G	SNP	0|0	0|1	34|0	4|5
Chr10	47242720	47242720	A	T	SNP	0|0	0|1	45|0	3|6
Chr10	47242835	47242835	A	G	SNP	0|0	0|1	40|0	8|8
Chr10	47242916	47242916	A	T	SNP	0|0	0|1	34|0	9|6
Chr10	47243179	47243179	A	G	SNP	0|0	0|1	31|0	1|7
Chr10	47243500	47243500	C	A	SNP	0|0	0|1	57|1	3|12
Chr10	47243547	47243547	A	C	SNP	0|0	0|1	68|0	3|13
Chr10	47243566	47243566	C	T	SNP	0|0	0|1	71|0	4|14
Chr10	47243694	47243694	C	T	SNP	0|0	0|1	57|1	3|16
Chr10	47243706	47243706	T	G	SNP	0|0	0|1	50|1	3|13
Chr10	47243933	47243933	C	T	SNP	0|0	0|1	45|0	6|20
Chr10	47244091	47244091	T	C	SNP	0|0	0|1	51|0	15|17
Chr10	47244190	47244190	C	G	SNP	0|0	0|1	51|1	10|18
Chr10	47244373	47244373	T	A	SNP	0|0	0|1	37|0	7|21
Chr10	47244491	47244491	T	C	SNP	0|0	0|1	54|1	4|13
Chr10	47245181	47245181	C	T	SNP	0|0	0|1	46|0	3|7
Chr10	47245510	47245510	A	G	SNP	0|0	0|1	40|0	2|7
Chr10	47245539	47245539	A	T	SNP	0|0	0|1	46|0	2|8
Chr10	47246036	47246036	C	A	SNP	0|0	0|1	26|0	4|5
Chr10	47246380	47246380	T	A	SNP	0|0	0|1	8|0	3|5
Chr10	47247475	47247475	C	T	SNP	0|0	0|1	10|0	3|7
Chr10	47247873	47247873	T	A	SNP	0|0	0|1	47|0	9|19
Chr10	47247970	47247970	A	G	SNP	0|0	0|1	69|0	13|13
Chr10	47248193	47248193	T	A	SNP	0|0	0|1	45|0	9|14
Chr10	47248726	47248726	T	G	SNP	0|0	0|1	51|0	9|9
Chr10	47248793	47248793	T	C	SNP	0|0	0|1	35|0	3|9
Chr10	47248800	47248800	T	C	SNP	0|0	0|1	35|0	3|8
Chr10	47249495	47249495	G	A	SNP	0|0	0|1	37|2	8|21
Chr10	47249522	47249522	A	G	SNP	0|0	0|1	41|2	7|19
Chr10	47249626	47249626	C	G	SNP	0|0	0|1	40|0	4|19
Chr10	47249769	47249769	T	G	SNP	0|0	0|1	49|1	7|11
Chr10	47249857	47249857	A	T	SNP	0|0	0|1	59|2	5|23
Chr10	47249925	47249925	G	C	SNP	0|0	0|1	79|2	5|19
Chr10	47249969	47249969	A	G	SNP	0|0	0|1	66|2	7|16

Chr10	47250145	47250145	T	A	SNP	0|0	0|1	63|0	12|14
Chr10	47250390	47250390	A	G	SNP	0|0	0|1	45|0	4|10
Chr10	47250407	47250407	C	A	SNP	0|0	0|1	43|0	5|9
Chr10	47250725	47250725	T	C	SNP	0|0	0|1	42|0	3|11
Chr10	47250794	47250794	A	C	SNP	0|0	0|1	37|0	3|12
Chr10	47250823	47250823	G	A	SNP	0|0	0|1	27|0	2|10
Chr10	47250826	47250826	T	C	SNP	0|0	0|1	27|0	2|9
Chr10	47251089	47251089	A	G	SNP	0|1	0|1	10|1	2|6
Chr10	47251872	47251872	T	A	SNP	0|0	0|1	24|0	1|7
Chr10	47252097	47252097	G	A	SNP	0|0	0|1	33|0	3|8
Chr10	47252142	47252142	A	G	SNP	0|0	0|1	38|0	3|11
Chr10	47252291	47252291	C	T	SNP	0|0	0|1	41|0	4|9
Chr10	47252593	47252593	C	A	SNP	0|0	0|1	62|0	8|11
Chr10	47252643	47252643	T	C	SNP	0|0	0|1	49|0	8|12
Chr10	47252706	47252706	A	G	SNP	0|0	0|1	62|0	10|11
Chr10	47252791	47252791	A	T	SNP	0|0	0|1	61|0	9|14
Chr10	47253104	47253104	T	A	SNP	0|0	0|1	47|1	5|16
Chr10	47253158	47253158	C	T	SNP	0|0	0|1	51|0	6|17
Chr10	47253218	47253218	T	C	SNP	0|0	0|1	48|1	6|15
Chr10	47253671	47253671	C	G	SNP	0|0	0|1	36|1	3|13
Chr10	47253790	47253790	A	T	SNP	0|0	0|1	52|0	5|8
Chr10	47253810	47253810	C	G	SNP	0|0	0|1	53|0	6|8
Chr10	47253892	47253892	C	T	SNP	0|0	0|1	51|0	10|9
Chr10	47253913	47253913	C	T	SNP	0|0	0|1	46|0	10|9
Chr10	47254108	47254108	G	A	SNP	0|0	0|1	50|0	7|9
Chr10	47258556	47258556	C	T	SNP	0|0	0|1	37|0	12|10
Chr10	47260460	47260460	T	A	SNP	0|0	0|1	30|2	3|6
Chr10	47267451	47267451	A	T	SNP	0|0	0|1	15|2	1|8
Chr10	47271380	47271380	T	C	SNP	0|0	0|1	33|0	4|8
Chr10	47279181	47279181	T	C	SNP	0|0	0|1	38|2	6|15
Chr10	47312528	47312528	A	G	SNP	0|0	0|1	44|0	9|5
Chr10	47333510	47333510	G	A	SNP	0|0	0|1	22|0	8|5
Chr10	47333975	47333975	G	T	SNP	0|0	0|1	23|0	1|7
Chr10	47334144	47334144	G	C	SNP	0|0	0|1	29|0	5|14
Chr10	47334147	47334147	C	G	SNP	0|0	0|1	30|0	5|15
Chr10	47334405	47334405	T	G	SNP	0|0	0|1	24|1	6|10
Chr10	47334480	47334480	C	A	SNP	0|0	0|1	41|1	2|17
Chr10	47335325	47335325	T	C	SNP	0|0	0|1	36|0	7|9
Chr10	47335389	47335389	C	T	SNP	0|0	0|1	54|0	9|8
Chr10	47335925	47335925	T	C	SNP	0|0	1|1	38|0	0|8
Chr10	47338067	47338067	C	T	SNP	0|0	0|1	21|0	5|7
Chr10	47339871	47339871	T	G	SNP	0|0	0|1	26|2	6|7

Chr10	47341328	47341328	G	A	SNP	0|0	0|1	33|2	3|8
Chr10	47341392	47341392	T	C	SNP	0|0	0|1	38|1	2|8
Chr10	47342112	47342112	C	T	SNP	0|1	0|1	25|4	3|6
Chr10	47342256	47342256	T	C	SNP	0|0	0|1	50|0	8|14
Chr10	47342292	47342292	G	A	SNP	0|0	0|1	42|0	6|10
Chr10	47342329	47342329	A	G	SNP	0|0	0|1	53|1	6|8
Chr10	47342357	47342357	G	A	SNP	0|0	0|1	53|1	4|8
Chr10	47342453	47342453	C	G	SNP	0|0	0|1	57|1	1|16
Chr10	47342454	47342454	C	T	SNP	0|0	0|1	55|1	1|16
Chr10	47342549	47342549	A	T	SNP	0|0	1|1	22|0	0|9
Chr10	47342617	47342617	T	G	SNP	0|0	0|1	29|1	2|10
Chr10	47342719	47342719	T	A	SNP	0|0	0|1	45|1	2|7
Chr10	47342806	47342806	A	G	SNP	0|0	0|1	47|0	3|10
Chr10	47342807	47342807	G	A	SNP	0|0	0|1	47|0	3|10
Chr10	47342814	47342814	A	G	SNP	0|0	0|1	45|0	4|10
Chr10	47342827	47342827	A	G	SNP	0|0	0|1	46|0	4|12
Chr10	47342841	47342841	T	C	SNP	0|0	0|1	42|0	4|12
Chr10	47342844	47342844	G	A	SNP	0|0	0|1	43|0	4|14
Chr10	47342890	47342890	C	T	SNP	0|0	0|1	44|0	6|12
Chr10	47342892	47342892	T	C	SNP	0|0	0|1	44|0	6|12
Chr10	47342933	47342933	A	G	SNP	0|0	0|1	48|0	7|14
Chr10	47342982	47342982	T	G	SNP	0|0	0|1	51|0	7|15
Chr10	47342994	47342994	C	T	SNP	0|0	0|1	53|0	6|17
Chr10	47342997	47342997	G	A	SNP	0|0	0|1	56|0	6|16
Chr10	47343007	47343007	T	C	SNP	0|0	0|1	56|0	6|15
Chr10	47343038	47343038	T	C	SNP	0|0	0|1	60|0	5|18
Chr10	47343056	47343056	A	C	SNP	0|0	0|1	55|0	3|19
Chr10	47343060	47343060	C	T	SNP	0|0	0|1	57|0	2|19
Chr10	47343064	47343064	A	C	SNP	0|0	0|1	59|0	2|19
Chr10	47343108	47343108	C	T	SNP	0|0	0|1	56|1	3|23
Chr10	47343116	47343116	G	C	SNP	0|0	0|1	52|1	3|23
Chr10	47343117	47343117	T	C	SNP	0|0	0|1	52|1	3|24
Chr10	47343140	47343140	G	A	SNP	0|0	0|1	49|2	6|18
Chr10	47343161	47343161	T	G	SNP	0|0	0|1	41|2	5|17
Chr10	47343216	47343216	G	A	SNP	0|0	0|1	42|3	10|15
Chr10	47343279	47343279	C	T	SNP	0|0	0|1	40|2	8|15
Chr10	47343310	47343310	A	C	SNP	0|0	0|1	39|1	12|17
Chr10	47343312	47343312	G	C	SNP	0|0	0|1	40|1	12|17
Chr10	47343360	47343360	A	G	SNP	0|0	0|1	30|1	8|9
Chr10	47343371	47343371	T	C	SNP	0|0	0|1	26|0	8|9
Chr10	47343373	47343373	A	G	SNP	0|0	0|1	24|0	8|9
Chr10	47343406	47343406	A	G	SNP	0|0	0|1	22|0	8|5

Chr10	47343898	47343898	T	G	SNP	0|0	0|1	25|1	4|6
Chr10	47343914	47343914	C	T	SNP	0|0	0|1	23|1	4|6
Chr10	47343927	47343927	C	T	SNP	0|0	0|1	25|1	4|6
Chr10	47343943	47343943	A	G	SNP	0|0	0|1	27|1	4|6
Chr10	47343967	47343967	A	T	SNP	0|0	0|1	26|0	4|5
Chr10	47343992	47343992	C	A	SNP	0|0	0|1	32|0	5|5
Chr10	47344018	47344018	G	A	SNP	0|0	0|1	29|0	2|6
Chr10	47344075	47344075	G	A	SNP	0|0	0|1	29|0	7|9
Chr10	47344174	47344174	T	C	SNP	0|0	0|1	35|0	13|9
Chr10	47344203	47344203	T	C	SNP	0|0	0|1	43|0	11|8
Chr10	47344233	47344233	A	G	SNP	0|0	0|1	43|0	8|7
Chr10	47344265	47344265	G	C	SNP	0|0	0|1	35|0	5|5
Chr10	47344287	47344287	T	C	SNP	0|0	0|1	34|0	6|4
Chr10	47344317	47344317	G	A	SNP	0|0	0|1	27|1	4|5
Chr10	47344326	47344326	G	A	SNP	0|0	0|1	26|0	3|5
Chr10	47344343	47344343	A	G	SNP	0|0	0|1	26|0	3|7
Chr10	47344422	47344422	A	G	SNP	0|0	0|1	27|0	6|6
Chr10	47344440	47344440	G	C	SNP	0|0	0|1	31|0	4|6
Chr10	47344457	47344457	T	C	SNP	0|0	0|1	29|0	4|6
Chr10	47344461	47344461	A	G	SNP	0|0	0|1	28|0	4|6
Chr10	47344469	47344469	C	T	SNP	0|0	0|1	31|0	4|6
Chr10	47344480	47344480	T	C	SNP	0|0	0|1	31|0	4|6
Chr10	47344490	47344490	C	G	SNP	0|0	0|1	28|0	5|4
Chr10	47344499	47344499	A	G	SNP	0|0	0|1	29|0	7|4
Chr10	47344905	47344905	C	T	SNP	0|0	0|1	30|0	6|6
Chr10	47344981	47344981	A	C	SNP	0|0	0|1	26|0	4|13
Chr10	47345058	47345058	T	C	SNP	0|0	0|1	22|0	3|10
Chr10	47345506	47345506	G	A	SNP	0|0	0|1	53|1	4|10
Chr10	47345620	47345620	G	A	SNP	0|0	0|1	46|0	12|14
Chr10	47345669	47345669	C	T	SNP	0|0	0|1	50|0	11|14
Chr10	47345684	47345684	C	G	SNP	0|0	0|1	53|0	11|14
Chr10	47345862	47345862	C	T	SNP	0|0	0|1	52|0	7|9
Chr10	47347126	47347126	G	A	SNP	0|0	0|1	45|0	4|10
Chr10	47347196	47347196	A	T	SNP	0|0	1|1	46|1	1|16
Chr10	47348624	47348624	T	C	SNP	0|0	0|1	33|1	7|14
Chr10	47349096	47349096	T	C	SNP	0|0	0|1	36|1	7|7
Chr10	47349207	47349207	A	T	SNP	0|0	0|1	25|0	7|4
Chr10	47349932	47349932	C	A	SNP	0|0	0|1	30|0	3|11
Chr10	47350822	47350822	C	T	SNP	0|0	0|1	31|0	7|11
Chr10	47350996	47350996	C	T	SNP	0|0	0|1	24|0	1|9
Chr10	47351019	47351019	A	C	SNP	0|0	0|1	21|0	4|10
Chr10	47351024	47351024	A	G	SNP	0|0	0|1	24|0	4|10

Chr10	47351081	47351081	T	C	SNP	0|0	0|1	24|1	7|13
Chr10	47351154	47351154	T	C	SNP	0|0	0|1	44|1	7|14
Chr10	47351272	47351272	G	T	SNP	0|1	0|1	45|5	3|16
Chr10	47351329	47351329	G	C	SNP	0|1	0|1	46|5	2|15
Chr10	47351334	47351334	C	T	SNP	0|1	0|1	46|5	3|16
Chr10	47351395	47351395	G	A	SNP	0|0	0|1	54|0	2|14
Chr10	47351435	47351435	G	A	SNP	0|0	0|1	53|0	3|14
Chr10	47351437	47351437	T	C	SNP	0|0	0|1	54|0	3|13
Chr10	47351439	47351439	G	A	SNP	0|0	0|1	55|0	3|13
Chr10	47351502	47351502	A	G	SNP	0|0	0|1	50|1	2|12
Chr10	47351594	47351594	G	A	SNP	0|1	0|1	46|5	3|12
Chr10	47351602	47351602	T	C	SNP	0|1	0|1	46|5	3|14
Chr10	47351721	47351721	A	G	SNP	0|0	0|1	37|1	7|6
Chr10	47351728	47351728	C	G	SNP	0|0	0|1	36|1	7|7
Chr10	47351763	47351763	T	C	SNP	0|0	0|1	34|1	5|6
Chr10	47351774	47351774	T	C	SNP	0|0	0|1	31|1	4|6
Chr10	47351785	47351785	C	T	SNP	0|0	0|1	28|1	4|5
Chr10	47351799	47351799	C	A	SNP	0|0	0|1	26|2	4|6
Chr10	47351837	47351837	C	T	SNP	0|1	0|1	20|2	3|7
Chr10	47351872	47351872	T	A	SNP	0|1	0|1	21|2	4|6
Chr10	47351972	47351972	C	T	SNP	0|0	0|1	19|0	6|3
Chr10	47352232	47352232	C	T	SNP	0|0	0|1	23|1	6|3
Chr10	47352389	47352389	A	G	SNP	0|0	0|1	29|1	4|5
Chr10	47352507	47352507	C	A	SNP	0|0	0|1	45|1	5|10
Chr10	47353504	47353504	A	T	SNP	0|0	0|1	34|0	3|13
Chr10	47353677	47353677	T	A	SNP	0|0	0|1	74|0	6|17
Chr10	47354119	47354119	C	T	SNP	0|0	0|1	60|0	9|11
Chr10	47354143	47354143	A	G	SNP	0|0	0|1	60|1	7|10
Chr10	47354287	47354287	A	G	SNP	0|0	0|1	44|0	2|6
Chr10	47355647	47355647	C	A	SNP	0|0	0|1	24|0	6|4
Chr10	47355719	47355719	A	G	SNP	0|0	0|1	23|0	5|8
Chr10	47356081	47356081	C	T	SNP	0|0	0|1	49|1	2|7
Chr10	47356128	47356128	C	A	SNP	0|0	0|1	47|1	2|10
Chr10	47356551	47356551	C	A	SNP	0|0	0|1	34|1	5|17
Chr10	47356555	47356555	C	G	SNP	0|0	0|1	34|1	5|17
Chr10	47356669	47356669	T	C	SNP	0|0	0|1	59|1	4|13
Chr10	47357129	47357129	C	T	SNP	0|0	0|1	48|2	11|10
Chr10	47357312	47357312	C	A	SNP	0|0	0|1	56|1	5|12
Chr10	47358163	47358163	G	A	SNP	0|0	0|1	34|0	8|11
Chr10	47359140	47359140	T	A	SNP	0|0	0|1	24|0	3|11
Chr10	47360060	47360060	A	T	SNP	0|0	0|1	36|2	8|21
Chr10	47360172	47360172	T	G	SNP	0|0	0|1	55|0	7|17

Chr10	47362630	47362630	G	A	SNP	0|0	0|1	80|3	8|13
Chr10	47362777	47362777	C	T	SNP	0|0	0|1	96|4	9|24
Chr10	47363386	47363386	A	G	SNP	0|0	0|1	86|1	4|13
Chr10	47363610	47363610	T	C	SNP	0|0	0|1	58|1	5|8
Chr10	47363778	47363778	T	C	SNP	0|0	0|1	68|1	3|7
Chr10	47363958	47363958	A	G	SNP	0|0	0|1	73|1	7|8
Chr10	47364064	47364064	G	C	SNP	0|0	0|1	48|0	5|8
Chr10	47364080	47364080	G	A	SNP	0|0	0|1	51|0	3|6
Chr10	47364143	47364143	G	T	SNP	0|0	0|1	47|0	6|9
Chr10	47365339	47365339	T	G	SNP	0|0	0|1	22|0	1|7
Chr10	47366245	47366245	G	A	SNP	0|0	0|1	69|0	4|17
Chr10	47366444	47366444	T	C	SNP	0|0	0|1	45|0	6|9
Chr10	47367136	47367136	T	C	SNP	0|0	0|1	52|0	4|6
Chr10	47368826	47368826	C	T	SNP	0|0	0|1	71|0	4|13
Chr10	47373536	47373536	A	T	SNP	0|0	0|1	31|0	3|7
Chr10	47373980	47373980	G	C	SNP	0|0	0|1	51|1	1|10
Chr10	47375429	47375429	G	T	SNP	0|0	0|1	43|2	4|13
Chr10	47376377	47376377	C	A	SNP	0|0	0|1	36|0	8|6
Chr10	47377463	47377463	T	A	SNP	0|0	0|1	26|0	5|8
Chr10	47379396	47379396	A	G	SNP	0|0	0|1	49|0	4|19
Chr10	47384285	47384285	G	A	SNP	0|0	0|1	42|0	3|12
Chr10	47384837	47384837	A	G	SNP	0|0	0|1	56|1	6|17
Chr10	47385874	47385874	G	T	SNP	0|0	0|1	53|0	4|10
Chr10	47386782	47386782	A	T	SNP	0|0	0|1	42|1	8|12
Chr10	47387061	47387061	T	A	SNP	0|0	0|1	24|0	4|4
Chr10	47404257	47404257	T	G	SNP	0|0	0|1	45|0	5|16
Chr10	47411579	47411579	G	A	SNP	0|0	0|1	31|0	4|8
Chr10	47443976	47443976	G	A	SNP	0|1	0|1	59|8	15|12
Chr10	47443995	47443995	T	C	SNP	0|1	0|1	54|8	15|14
Chr10	47444519	47444519	A	G	SNP	0|0	0|1	51|0	12|11
Chr10	47453790	47453790	A	C	SNP	0|0	0|1	21|0	7|7
Chr10	47463036	47463036	C	G	SNP	0|0	0|1	33|0	5|5
Chr10	47463442	47463442	C	A	SNP	0|0	0|1	40|0	6|4
Chr10	47464273	47464273	G	C	SNP	0|0	0|1	22|0	4|4
Chr10	47464531	47464531	A	T	SNP	0|1	0|1	12|1	2|12
Chr10	47464533	47464533	A	C	SNP	0|1	0|1	12|1	2|12
Chr10	47464803	47464803	C	G	SNP	0|0	0|1	37|1	5|11
Chr10	47464856	47464856	G	A	SNP	0|0	0|1	31|0	4|8
Chr10	47464880	47464880	T	C	SNP	0|0	0|1	40|0	4|8
Chr10	47465022	47465022	G	C	SNP	0|0	0|1	23|0	5|9
Chr10	47465071	47465071	A	G	SNP	0|0	0|1	22|0	3|11
Chr10	47465087	47465087	A	G	SNP	0|0	0|1	21|0	1|11

Chr10	47465157	47465157	C	T	SNP	0|0	0|1	17|0	1|15
Chr10	47465168	47465168	G	A	SNP	0|0	0|1	18|0	1|12
Chr10	47465379	47465379	G	A	SNP	0|0	0|1	14|0	1|7
Chr10	47465725	47465725	C	T	SNP	0|0	0|1	46|1	3|8
Chr10	47465748	47465748	A	G	SNP	0|0	0|1	45|1	4|10
Chr10	47465885	47465885	T	A	SNP	0|0	0|1	51|0	9|10
Chr10	47465911	47465911	T	C	SNP	0|0	0|1	51|0	8|11
Chr10	47465928	47465928	T	C	SNP	0|0	0|1	50|0	6|13
Chr10	47466588	47466588	T	C	SNP	0|1	0|1	18|3	2|8
Chr10	47468214	47468214	G	T	SNP	0|0	0|1	53|0	9|12
Chr10	47469484	47469484	C	T	SNP	0|0	0|1	48|0	6|8
Chr10	47469679	47469679	C	T	SNP	0|0	0|1	43|0	14|6
Chr10	47469722	47469722	G	A	SNP	0|0	0|1	45|0	14|12
Chr10	47470185	47470185	G	A	SNP	0|0	0|1	46|0	5|10
Chr10	47470586	47470586	T	C	SNP	0|0	0|1	63|1	6|12
Chr10	47470695	47470695	C	T	SNP	0|0	0|1	70|3	10|20
Chr10	47470898	47470898	A	G	SNP	0|0	0|1	70|1	8|13
Chr10	47471240	47471240	C	A	SNP	0|0	0|1	50|0	2|9
Chr10	47471401	47471401	A	G	SNP	0|0	0|1	42|0	4|14
Chr10	47471847	47471847	T	C	SNP	0|0	0|1	30|0	2|9
Chr10	47472016	47472016	C	T	SNP	0|1	0|1	27|2	2|7
Chr10	47472035	47472035	A	G	SNP	0|1	0|1	24|2	2|6
Chr10	47473223	47473223	C	T	SNP	0|0	0|1	62|1	6|10
Chr10	47473305	47473305	C	T	SNP	0|0	0|1	61|4	8|6
Chr10	47475139	47475139	T	C	SNP	0|0	0|1	44|0	3|6
Chr10	47475833	47475833	A	G	SNP	0|0	0|1	20|0	6|16
Chr10	47476021	47476021	G	C	SNP	0|0	0|1	43|0	9|9
Chr10	47477460	47477460	C	T	SNP	0|0	0|1	14|0	3|8
Chr10	47482989	47482989	G	A	SNP	0|0	0|1	62|0	4|12
Chr10	47483007	47483007	G	A	SNP	0|0	0|1	62|0	4|15
Chr10	47484949	47484949	G	C	SNP	0|0	0|1	34|1	3|13
Chr10	47485080	47485080	G	A	SNP	0|0	0|1	61|0	2|14
Chr10	47487026	47487026	A	C	SNP	0|0	0|1	42|0	8|13
Chr10	47487341	47487341	C	T	SNP	0|0	0|1	48|0	4|9
Chr10	47487418	47487418	C	G	SNP	0|0	0|1	39|0	2|7
Chr10	47487589	47487589	T	A	SNP	0|0	0|1	50|0	8|9
Chr10	47487777	47487777	G	C	SNP	0|0	0|1	60|1	11|10
Chr10	47488249	47488249	C	G	SNP	0|0	0|1	46|0	6|15
Chr10	47489116	47489116	A	G	SNP	0|0	0|1	35|0	7|4
Chr10	47489721	47489721	G	A	SNP	0|0	0|1	45|0	3|7
Chr10	47490270	47490270	C	T	SNP	0|0	0|1	25|0	3|10
Chr10	47491127	47491127	T	C	SNP	0|0	0|1	47|0	6|5

Chr10	47491393	47491393	G	A	SNP	0|0	0|1	71|0	6|16
Chr10	47491540	47491540	A	T	SNP	0|0	0|1	55|0	5|17
Chr10	47491751	47491751	A	T	SNP	0|0	0|1	55|0	7|8
Chr10	47491806	47491806	A	T	SNP	0|0	0|1	36|0	7|5
Chr10	47492207	47492207	G	T	SNP	0|0	0|1	22|0	3|7
Chr10	47492251	47492251	T	C	SNP	0|0	0|1	22|0	4|8
Chr10	47492328	47492328	T	C	SNP	0|0	0|1	45|0	5|6
Chr10	47492384	47492384	A	G	SNP	0|0	0|1	51|0	9|10
Chr10	47492409	47492409	G	A	SNP	0|0	0|1	41|0	11|7
Chr10	47492595	47492595	G	A	SNP	0|0	0|1	54|2	5|6
Chr10	47492664	47492664	A	G	SNP	0|0	0|1	56|1	7|4
Chr10	47492668	47492668	C	T	SNP	0|0	0|1	57|1	7|4
Chr10	47492674	47492674	G	T	SNP	0|0	0|1	49|1	7|4
Chr10	47492678	47492678	T	A	SNP	0|0	0|1	49|1	7|3
Chr10	47492687	47492687	C	T	SNP	0|0	0|1	49|1	7|3
Chr10	47492692	47492692	C	T	SNP	0|0	0|1	49|0	7|3
Chr10	47492693	47492693	G	A	SNP	0|0	0|1	47|0	7|3
Chr10	47493043	47493043	T	C	SNP	0|0	0|1	71|0	5|3
Chr10	47493076	47493076	T	G	SNP	0|0	0|1	64|0	4|4
Chr10	47493081	47493081	C	T	SNP	0|0	0|1	67|0	5|4
Chr10	47493084	47493084	C	T	SNP	0|0	0|1	64|0	7|4
Chr10	47493087	47493087	T	G	SNP	0|0	0|1	67|0	7|4
Chr10	47493097	47493097	A	G	SNP	0|0	0|1	62|0	9|4
Chr10	47493098	47493098	T	C	SNP	0|0	0|1	60|0	9|4
Chr10	47493107	47493107	A	G	SNP	0|0	0|1	62|0	9|4
Chr10	47493108	47493108	C	T	SNP	0|0	0|1	65|0	9|4
Chr10	47493126	47493126	T	C	SNP	0|0	0|1	65|0	9|5
Chr10	47493175	47493175	A	G	SNP	0|0	0|1	51|0	6|9
Chr10	47493176	47493176	T	C	SNP	0|0	0|1	51|0	6|9
Chr10	47493202	47493202	G	T	SNP	0|0	0|1	52|0	10|5
Chr10	47493223	47493223	A	G	SNP	0|0	0|1	56|0	10|6
Chr10	47493224	47493224	C	T	SNP	1|1	0|1	0|57	6|10
Chr10	47493226	47493226	G	A	SNP	0|0	0|1	55|0	9|6
Chr10	47493232	47493232	C	G	SNP	0|0	0|1	52|0	8|6
Chr10	47493247	47493247	G	A	SNP	0|0	0|1	54|0	5|6
Chr10	47493248	47493248	C	T	SNP	0|0	0|1	54|0	4|6
Chr10	47493256	47493256	A	G	SNP	0|0	0|1	56|0	5|6
Chr10	47493272	47493272	G	C	SNP	0|0	0|1	53|0	5|5
Chr10	47493282	47493282	T	C	SNP	0|0	0|1	52|0	5|5
Chr10	47493319	47493319	A	G	SNP	0|0	0|1	56|0	6|4
Chr10	47493336	47493336	A	G	SNP	0|0	0|1	60|0	8|4
Chr10	47493337	47493337	T	A	SNP	0|0	0|1	60|0	8|5

Chr10	47493371	47493371	T	C	SNP	0|0	0|1	61|0	6|7
Chr10	47493419	47493419	A	G	SNP	0|0	0|1	72|0	7|14
Chr10	47493427	47493427	C	T	SNP	0|0	0|1	70|0	8|14
Chr10	47493431	47493431	T	C	SNP	0|0	0|1	69|0	8|14
Chr10	47493443	47493443	T	G	SNP	0|0	0|1	73|0	9|13
Chr10	47493476	47493476	A	C	SNP	0|0	0|1	66|0	9|10
Chr10	47493484	47493484	G	A	SNP	0|0	0|1	62|0	6|10
Chr10	47493497	47493497	T	C	SNP	0|0	0|1	60|0	6|8
Chr10	47493499	47493499	G	A	SNP	0|0	0|1	60|0	6|8
Chr10	47493501	47493501	T	C	SNP	0|0	0|1	57|0	6|8
Chr10	47493510	47493510	G	T	SNP	0|0	0|1	55|0	6|8
Chr10	47493512	47493512	G	A	SNP	0|0	0|1	56|0	6|8
Chr10	47493534	47493534	A	T	SNP	0|0	0|1	56|0	6|3
Chr10	47493536	47493536	A	G	SNP	0|0	0|1	57|0	6|3
Chr10	47493654	47493654	C	T	SNP	0|0	0|1	46|0	3|9
Chr10	47493677	47493677	T	C	SNP	0|0	0|1	45|0	3|9
Chr10	47493682	47493682	A	G	SNP	0|0	0|1	42|0	3|9
Chr10	47493684	47493684	C	T	SNP	0|0	0|1	42|0	3|9
Chr10	47493693	47493693	T	C	SNP	0|0	0|1	37|0	3|9
Chr10	47493698	47493698	G	A	SNP	0|0	0|1	36|0	3|9
Chr10	47493706	47493706	T	C	SNP	0|0	0|1	32|0	3|9
Chr10	47493718	47493718	T	C	SNP	0|0	0|1	33|0	3|8
Chr10	47493725	47493725	C	T	SNP	0|0	0|1	29|0	3|10
Chr10	47494592	47494592	C	G	SNP	0|0	0|1	26|0	5|3
Chr10	47494600	47494600	T	C	SNP	0|0	0|1	30|0	5|3
Chr10	47496219	47496219	G	C	SNP	0|0	0|1	56|2	4|10
Chr10	47499368	47499368	G	C	SNP	0|0	0|1	9|0	6|3
Chr10	47501189	47501189	T	G	SNP	0|0	0|1	29|0	1|8
Chr10	47501741	47501741	A	T	SNP	0|0	0|1	36|0	7|9
Chr10	47505696	47505696	C	A	SNP	0|0	0|1	29|0	5|14
Chr10	47506002	47506002	A	G	SNP	0|0	0|1	40|0	5|11
Chr10	47506017	47506017	G	T	SNP	0|0	0|1	42|0	6|13
Chr10	47506149	47506149	C	A	SNP	0|0	1|1	29|0	1|15
Chr10	47506199	47506199	T	C	SNP	0|0	1|1	19|0	0|14
Chr10	47506255	47506255	A	G	SNP	0|0	1|1	14|0	0|9
Chr10	47506331	47506331	T	C	SNP	0|0	0|1	15|0	2|7
Chr10	47506653	47506653	T	C	SNP	0|0	0|1	24|0	3|5
Chr10	47506658	47506658	G	A	SNP	0|0	0|1	26|0	3|6
Chr10	47509418	47509418	C	T	SNP	0|0	1|1	30|0	0|13
Chr10	47509716	47509716	G	A	SNP	0|0	0|1	38|1	4|4
Chr10	47561624	47561624	C	T	SNP	1|1	0|1	0|56	15|8
Chr10	47628665	47628665	T	C	SNP	0|0	0|1	54|1	6|11

Chr10	47629115	47629115	C	T	SNP	0|0	0|1	26|0	9|4
Chr10	47662787	47662787	C	G	SNP	0|1	0|0	54|27	21|0
Chr10	47662793	47662793	A	G	SNP	0|1	0|0	57|28	21|0
Chr10	47662810	47662810	G	A	SNP	0|1	0|0	54|32	21|1
Chr10	47662887	47662887	C	T	SNP	0|1	0|1	54|27	19|2
Chr10	47666092	47666092	G	T	SNP	0|0	0|1	20|0	3|7
Chr10	47667220	47667220	C	A	SNP	0|0	0|1	39|0	3|18
Chr10	47667288	47667288	G	C	SNP	0|0	0|1	43|0	2|13
Chr10	47667463	47667463	T	G	SNP	0|1	0|1	24|3	1|12
Chr10	47667932	47667932	T	G	SNP	0|0	0|1	23|1	3|17
Chr10	47668746	47668746	T	C	SNP	0|0	0|1	38|0	2|8
Chr10	47668939	47668939	T	G	SNP	0|0	0|1	33|0	5|4
Chr10	47668955	47668955	G	A	SNP	0|0	0|1	36|0	7|4
Chr10	47669092	47669092	A	C	SNP	0|0	0|1	36|1	5|9
Chr10	47669141	47669141	C	A	SNP	0|0	0|1	33|0	2|7
Chr10	47669850	47669850	A	G	SNP	0|0	0|1	45|1	2|7
Chr10	47669985	47669985	T	C	SNP	0|0	0|1	45|1	3|5
Chr10	47670072	47670072	A	T	SNP	0|0	0|1	35|0	7|7
Chr10	47670292	47670292	T	C	SNP	0|0	0|1	50|0	6|16
Chr10	47670533	47670533	C	T	SNP	0|0	0|1	33|0	4|5
Chr10	47670545	47670545	C	T	SNP	0|0	0|1	30|0	4|6
Chr10	47670588	47670588	G	C	SNP	0|0	0|1	23|0	4|4
Chr10	47670692	47670692	G	T	SNP	0|0	0|1	15|0	4|6
Chr10	47670695	47670695	C	T	SNP	0|0	0|1	15|0	4|5
Chr10	47671049	47671049	C	T	SNP	0|0	1|1	17|0	0|11
Chr10	47671263	47671263	A	G	SNP	0|0	1|1	20|1	0|10
Chr10	47671407	47671407	T	C	SNP	0|0	0|1	14|0	4|6
Chr10	47673136	47673136	C	T	SNP	0|0	0|1	29|0	3|5
Chr10	47674939	47674939	A	G	SNP	0|0	0|1	55|0	7|19
Chr10	47679456	47679456	T	C	SNP	0|1	0|1	25|2	3|13
Chr10	47679457	47679457	C	T	SNP	0|1	0|1	26|2	3|13
Chr10	47679791	47679791	T	C	SNP	0|0	0|1	33|3	2|11
Chr10	47687666	47687666	C	A	SNP	0|0	0|1	39|0	2|11
Chr10	47688663	47688663	T	A	SNP	0|0	0|1	63|0	6|12
Chr10	47690352	47690352	G	A	SNP	0|0	0|1	37|0	5|10
Chr10	47690714	47690714	T	C	SNP	0|1	0|1	31|5	4|9
Chr10	47693033	47693033	C	A	SNP	0|0	0|1	22|0	6|8
Chr10	47693927	47693927	T	A	SNP	0|0	0|1	39|2	3|11
Chr10	47694287	47694287	T	C	SNP	0|0	0|1	49|1	6|13
Chr10	47694306	47694306	A	T	SNP	0|0	0|1	46|1	5|12
Chr10	47694399	47694399	A	C	SNP	0|0	0|1	37|1	6|12
Chr10	47694947	47694947	C	T	SNP	0|0	0|1	67|0	8|6

Chr10	47695430	47695430	T	C	SNP	0|0	0|1	68|1	10|17
Chr10	47695977	47695977	G	A	SNP	0|0	0|1	50|0	6|17
Chr10	47699267	47699267	A	G	SNP	0|0	0|1	33|1	5|4
Chr10	47701180	47701180	A	T	SNP	0|0	0|1	8|0	5|3
Chr10	47701442	47701442	A	G	SNP	0|0	0|1	27|1	2|8
Chr10	47701538	47701538	A	T	SNP	0|0	0|1	42|0	1|12
Chr10	47706556	47706556	G	A	SNP	0|1	0|1	22|4	7|6
Chr10	47716007	47716007	G	A	SNP	0|0	0|1	34|1	1|8
Chr10	47716533	47716533	T	A	SNP	0|0	0|1	37|0	6|10
Chr10	47716772	47716772	G	A	SNP	0|0	0|1	60|0	4|12
Chr10	47720849	47720849	C	A	SNP	0|0	0|1	39|0	3|12
Chr10	47726985	47726985	C	T	SNP	0|0	0|1	44|0	4|14
Chr10	47733135	47733135	A	G	SNP	0|1	0|1	20|2	4|5
Chr10	47733309	47733309	T	A	SNP	0|1	0|1	19|2	3|9
Chr10	47733668	47733668	T	A	SNP	0|0	0|1	17|0	1|7
Chr10	47735359	47735359	T	A	SNP	0|0	0|1	12|0	3|5
Chr10	47735360	47735360	T	A	SNP	0|0	0|1	12|0	3|6
Chr10	47735456	47735456	A	C	SNP	0|0	0|1	21|0	5|7
Chr10	47735589	47735589	G	C	SNP	0|0	0|1	20|0	2|9
Chr10	47735595	47735595	G	A	SNP	0|0	0|1	21|0	1|9
Chr10	47735631	47735631	A	G	SNP	0|0	0|1	23|0	1|9
Chr10	47735792	47735792	G	A	SNP	0|0	0|1	24|0	2|11
Chr10	47735793	47735793	G	A	SNP	0|0	0|1	24|0	2|11
Chr10	47736134	47736134	T	C	SNP	0|0	0|1	23|0	4|5
Chr10	47736135	47736135	T	G	SNP	0|0	0|1	23|0	4|5
Chr10	47736147	47736147	T	C	SNP	0|0	0|1	25|0	4|5
Chr10	47736413	47736413	C	T	SNP	0|0	0|1	14|0	3|6
Chr10	47736490	47736490	G	A	SNP	0|0	0|1	19|0	2|8
Chr10	47736507	47736507	G	C	SNP	0|0	0|1	22|0	2|7
Chr10	47736511	47736511	T	C	SNP	0|0	0|1	22|0	2|7
Chr10	47736513	47736513	G	A	SNP	0|0	0|1	22|0	2|7
Chr10	47736589	47736589	A	G	SNP	0|0	0|1	15|0	2|9
Chr10	47736603	47736603	A	G	SNP	0|0	0|1	13|0	2|8
Chr10	47738196	47738196	A	G	SNP	0|0	0|1	34|0	6|8
Chr10	47740299	47740299	T	A	SNP	0|0	0|1	25|0	3|7
Chr10	47740768	47740768	C	G	SNP	0|0	1|1	15|0	0|10
Chr10	47741129	47741129	T	C	SNP	0|0	0|1	36|1	2|6
Chr10	47741169	47741169	A	G	SNP	0|0	0|1	44|0	2|9
Chr10	47743184	47743184	C	T	SNP	0|0	0|1	41|2	6|9
Chr10	47744576	47744576	A	G	SNP	0|0	0|1	27|0	5|18
Chr10	47744777	47744777	A	T	SNP	0|0	0|1	33|0	4|17
Chr10	47746660	47746660	C	T	SNP	0|0	0|1	55|0	5|5

Chr10	47747874	47747874	A	C	SNP	0|0	0|1	66|0	8|11
Chr10	47748412	47748412	G	C	SNP	0|0	0|1	70|0	8|14
Chr10	47748441	47748441	T	C	SNP	0|0	0|1	73|0	7|14
Chr10	47748642	47748642	T	A	SNP	0|0	0|1	57|0	9|11
Chr10	47748764	47748764	G	C	SNP	0|0	0|1	53|0	3|11
Chr10	47748768	47748768	G	A	SNP	0|0	0|1	52|0	3|14
Chr10	47748864	47748864	A	G	SNP	0|0	0|1	56|0	6|12
Chr10	47749014	47749014	G	C	SNP	0|0	0|1	79|0	13|21
Chr10	47749260	47749260	T	C	SNP	0|0	0|1	68|0	7|18
Chr10	47749581	47749581	G	A	SNP	0|0	0|1	60|0	6|10
Chr10	47749583	47749583	T	A	SNP	0|0	0|1	62|0	6|9
Chr10	47749935	47749935	T	G	SNP	0|0	0|1	72|0	3|10
Chr10	47750431	47750431	T	C	SNP	0|0	0|1	51|0	10|13
Chr10	47750696	47750696	G	A	SNP	0|0	0|1	45|0	6|11
Chr10	47752102	47752102	A	G	SNP	0|0	0|1	41|1	4|8
Chr10	47752635	47752635	C	A	SNP	0|0	0|1	65|0	4|13
Chr10	47754214	47754214	A	G	SNP	0|0	0|1	34|0	2|11
Chr10	47754234	47754234	G	T	SNP	0|0	0|1	28|0	3|10
Chr10	47754286	47754286	C	A	SNP	0|0	0|1	45|0	5|8
Chr10	47754815	47754815	T	G	SNP	0|0	0|1	27|0	4|6
Chr10	47755199	47755199	T	C	SNP	0|0	0|1	10|0	2|6
Chr10	47755857	47755857	A	T	SNP	0|0	0|1	18|0	4|4
Chr10	47756066	47756066	A	T	SNP	0|0	0|1	11|0	3|10
Chr10	47761315	47761315	C	T	SNP	0|0	0|1	54|0	4|15
Chr10	47764398	47764398	G	C	SNP	0|0	0|1	32|0	4|6
Chr10	47764565	47764565	A	G	SNP	0|0	0|1	30|0	4|11
Chr10	47765147	47765147	G	A	SNP	0|0	0|1	91|1	11|17
Chr10	47768770	47768770	T	C	SNP	0|0	0|1	38|0	3|10
Chr10	47769038	47769038	T	C	SNP	0|0	0|1	11|0	3|5
Chr10	47769502	47769502	C	T	SNP	0|0	0|1	36|2	1|8
Chr10	47769696	47769696	G	A	SNP	0|0	0|1	15|1	2|10
Chr10	47772740	47772740	T	A	SNP	0|0	0|1	12|0	1|9
Chr10	47773240	47773240	C	A	SNP	0|0	0|1	16|0	3|5
Chr10	47773347	47773347	T	C	SNP	0|0	0|1	26|0	6|9
Chr10	47773735	47773735	G	A	SNP	0|0	0|1	43|0	6|17
Chr10	47773825	47773825	A	C	SNP	0|0	0|1	42|0	7|18
Chr10	47774069	47774069	C	A	SNP	0|0	0|1	37|0	2|8
Chr10	47774626	47774626	T	A	SNP	0|0	0|1	71|0	3|19
Chr10	47775410	47775410	T	G	SNP	0|0	0|1	38|0	7|12
Chr10	47776017	47776017	G	A	SNP	0|0	0|1	63|0	14|23
Chr10	47776239	47776239	C	A	SNP	0|0	0|1	64|1	4|26
Chr10	47776257	47776257	C	G	SNP	0|0	0|1	67|1	5|26

Chr10	47776512	47776512	A	C	SNP	0|0	0|1	51|1	6|23
Chr10	47776557	47776557	C	A	SNP	0|0	0|1	45|0	5|22
Chr10	47777765	47777765	C	A	SNP	0|0	0|1	50|0	1|14
Chr10	47777931	47777931	G	T	SNP	0|0	0|1	54|0	6|5
Chr10	47777991	47777991	C	T	SNP	0|0	0|1	45|0	4|10
Chr10	47778559	47778559	T	G	SNP	0|0	0|1	22|0	2|14
Chr10	47778642	47778642	T	C	SNP	0|0	0|1	18|0	2|7
Chr10	47779235	47779235	A	T	SNP	0|0	0|1	23|0	2|8
Chr10	47779347	47779347	T	A	SNP	0|0	0|1	28|0	4|6
Chr10	47779369	47779369	T	A	SNP	0|0	0|1	30|0	4|7
Chr10	47779730	47779730	G	A	SNP	0|0	0|1	65|8	5|14
Chr10	47779915	47779915	C	T	SNP	0|0	0|1	58|0	4|14
Chr10	47780021	47780021	G	T	SNP	0|0	0|1	50|0	2|8
Chr10	47780252	47780252	T	C	SNP	0|0	0|1	54|0	4|7
Chr10	47780302	47780302	G	A	SNP	0|0	0|1	56|0	5|11
Chr10	47780381	47780381	G	C	SNP	0|0	0|1	66|0	8|19
Chr10	47780389	47780389	T	A	SNP	0|0	0|1	62|0	7|20
Chr10	47780587	47780587	T	C	SNP	0|0	0|1	92|0	7|19
Chr10	47780620	47780620	G	A	SNP	0|0	0|1	82|0	6|16
Chr10	47781656	47781656	A	G	SNP	0|0	0|1	39|0	4|13
Chr10	47781893	47781893	T	A	SNP	0|0	0|1	41|0	5|4
Chr10	47781945	47781945	G	C	SNP	0|0	0|1	32|0	5|6
Chr10	47781964	47781964	G	A	SNP	0|0	0|1	24|0	5|4
Chr10	47781965	47781965	C	T	SNP	0|0	0|1	24|0	5|5
Chr10	47781971	47781971	A	C	SNP	0|0	0|1	25|0	5|5
Chr10	47781978	47781978	G	A	SNP	0|0	0|1	21|0	3|5
Chr10	47781983	47781983	C	T	SNP	0|0	0|1	21|0	3|5
Chr10	47781994	47781994	C	T	SNP	0|0	0|1	24|0	3|5
Chr10	47782187	47782187	G	A	SNP	0|0	0|1	19|0	4|7
Chr10	47782199	47782199	T	C	SNP	0|0	0|1	21|0	4|6
Chr10	47783978	47783978	G	T	SNP	0|0	0|1	55|0	7|12
Chr10	47784005	47784005	C	T	SNP	0|0	0|1	49|0	5|16
Chr10	47784035	47784035	G	A	SNP	0|0	0|1	45|0	6|14
Chr10	47784041	47784041	T	C	SNP	0|0	0|1	46|0	6|15
Chr10	47784083	47784083	G	C	SNP	0|0	0|1	35|1	4|18
Chr10	47784152	47784152	G	A	SNP	0|0	0|1	41|0	2|14
Chr10	47784173	47784173	G	A	SNP	0|0	0|1	40|0	1|12
Chr10	47784251	47784251	A	C	SNP	0|0	0|1	41|0	1|12
Chr10	47784356	47784356	G	A	SNP	0|0	0|1	41|0	2|16
Chr10	47784461	47784461	A	T	SNP	0|0	0|1	51|0	2|9
Chr10	47784980	47784980	G	T	SNP	0|0	0|1	47|0	8|22
Chr10	47785058	47785058	T	G	SNP	0|0	0|1	42|0	4|11

Chr10	47785780	47785780	A	G	SNP	0|0	0|1	13|0	12|10
Chr10	47786295	47786295	C	T	SNP	0|0	0|1	19|0	1|8
Chr10	47786321	47786321	G	C	SNP	0|0	0|1	24|0	2|9
Chr10	47787323	47787323	T	A	SNP	0|0	0|1	43|0	2|15
Chr10	47787390	47787390	C	A	SNP	0|0	0|1	50|0	2|16
Chr10	47787394	47787394	T	G	SNP	0|0	0|1	49|0	2|14
Chr10	47787803	47787803	G	A	SNP	0|0	0|1	33|0	5|15
Chr10	47788430	47788430	A	T	SNP	0|0	0|1	50|0	2|9
Chr10	47788657	47788657	A	G	SNP	0|0	0|1	40|0	3|17
Chr10	47789208	47789208	G	T	SNP	0|0	0|1	46|0	2|14
Chr10	47789866	47789866	G	C	SNP	0|0	0|1	32|0	8|9
Chr10	47792562	47792562	A	C	SNP	0|0	0|1	60|0	1|9
Chr10	47793126	47793126	T	C	SNP	0|0	0|1	53|0	2|17
Chr10	47793449	47793449	T	A	SNP	0|0	0|1	66|0	2|9
Chr10	47793759	47793759	T	A	SNP	0|0	0|1	62|2	7|12
Chr10	47794146	47794146	T	A	SNP	0|0	0|1	18|0	4|9
Chr10	47794797	47794797	G	A	SNP	0|0	0|1	54|1	4|20
Chr10	47796128	47796128	T	C	SNP	0|0	0|1	42|0	3|13
Chr10	47796286	47796286	G	A	SNP	0|0	0|1	35|0	8|11
Chr10	47796615	47796615	T	C	SNP	0|0	0|1	21|0	2|6
Chr10	47796627	47796627	G	A	SNP	0|0	1|1	23|0	0|12
Chr10	47797732	47797732	G	T	SNP	0|0	0|1	64|0	6|16
Chr10	47799129	47799129	A	G	SNP	0|0	0|1	53|0	10|20
Chr10	47800187	47800187	C	G	SNP	0|0	0|1	48|0	4|14
Chr10	47800191	47800191	C	T	SNP	0|0	0|1	48|0	3|15
Chr10	47801332	47801332	G	A	SNP	0|0	0|1	35|0	6|12
Chr10	47801334	47801334	C	T	SNP	0|0	0|1	35|0	6|12
Chr10	47801385	47801385	C	T	SNP	0|0	0|1	29|0	4|9
Chr10	47801947	47801947	G	A	SNP	0|0	0|1	57|0	7|19
Chr10	47802686	47802686	A	G	SNP	0|0	0|1	40|0	1|8
Chr10	47803593	47803593	T	A	SNP	0|0	0|1	29|1	2|8
Chr10	47803712	47803712	G	A	SNP	0|0	0|1	42|0	7|15
Chr10	47804075	47804075	A	G	SNP	0|0	0|1	43|0	6|19
Chr10	47806039	47806039	A	G	SNP	0|0	1|1	20|0	2|15
Chr10	47806108	47806108	G	A	SNP	0|0	0|1	25|0	3|14
Chr10	47806458	47806458	T	C	SNP	0|0	0|1	42|0	3|8
Chr10	47806468	47806468	T	C	SNP	0|0	0|1	46|0	4|7
Chr10	47806604	47806604	G	A	SNP	0|0	0|1	37|0	4|16
Chr10	47807361	47807361	T	C	SNP	0|0	0|1	34|0	11|5
Chr10	47808226	47808226	G	C	SNP	0|0	0|1	29|0	2|7
Chr10	47808549	47808549	T	C	SNP	0|0	0|1	30|0	4|8
Chr10	47808560	47808560	C	A	SNP	0|0	0|1	28|0	3|7

Chr10	47808580	47808580	G	A	SNP	0|0	0|1	29|0	3|5
Chr10	47809881	47809881	C	T	SNP	0|0	0|1	50|0	6|12
Chr10	47809933	47809933	A	G	SNP	0|0	0|1	53|0	3|12
Chr10	47810950	47810950	T	C	SNP	0|0	0|1	42|1	6|12
Chr10	47811613	47811613	T	A	SNP	0|0	0|1	26|1	3|12
Chr10	47811646	47811646	A	G	SNP	0|0	0|1	26|0	2|9
Chr10	47813969	47813969	T	C	SNP	0|0	0|1	8|0	1|11
Chr10	47816262	47816262	T	G	SNP	0|0	0|1	35|0	6|12
Chr10	47816876	47816876	A	T	SNP	0|0	0|1	64|0	3|14
Chr10	47817161	47817161	G	A	SNP	0|0	0|1	41|2	7|10
Chr10	47817388	47817388	A	C	SNP	0|0	0|1	61|1	2|6
Chr10	47818215	47818215	G	A	SNP	0|0	0|1	43|0	8|10
Chr10	47818262	47818262	T	C	SNP	0|0	0|1	36|0	8|10
Chr10	47818380	47818380	C	T	SNP	0|1	0|1	13|1	3|11
Chr10	47818429	47818429	C	T	SNP	0|0	0|1	15|1	5|13
Chr10	47818719	47818719	A	C	SNP	0|0	0|1	36|0	2|8
Chr10	47818893	47818893	C	A	SNP	0|0	0|1	46|0	2|16
Chr10	47819071	47819071	C	A	SNP	0|0	0|1	60|1	5|24
Chr10	47819090	47819090	T	C	SNP	0|0	0|1	53|1	4|21
Chr10	47819340	47819340	T	G	SNP	0|0	0|1	43|1	4|4
Chr10	47819713	47819713	A	C	SNP	0|0	0|1	26|0	5|6
Chr10	47821142	47821142	A	C	SNP	0|0	0|1	28|0	5|8
Chr10	47822269	47822269	C	T	SNP	0|0	0|1	23|0	2|6
Chr10	47822289	47822289	A	T	SNP	0|0	0|1	20|0	2|9
Chr10	47823575	47823575	A	G	SNP	0|0	0|1	19|0	1|7
Chr10	47823709	47823709	T	G	SNP	0|0	0|1	30|0	5|8
Chr10	47823911	47823911	T	C	SNP	0|0	0|1	29|0	8|10
Chr10	47824046	47824046	C	T	SNP	1|1	0|1	0|41	8|11
Chr10	47824386	47824386	A	G	SNP	0|0	0|1	48|0	1|15
Chr10	47824510	47824510	T	C	SNP	0|0	0|1	42|0	2|12
Chr10	47826214	47826214	T	C	SNP	0|0	0|1	24|0	2|9
Chr10	47828402	47828402	G	C	SNP	0|0	0|1	29|0	3|11
Chr10	47828801	47828801	C	T	SNP	0|0	0|1	52|1	4|9
Chr10	47828904	47828904	T	A	SNP	0|0	0|1	49|1	7|10
Chr10	47830191	47830191	C	T	SNP	0|0	0|1	41|0	6|10
Chr10	47833366	47833366	A	G	SNP	0|0	0|1	48|1	5|10
Chr10	47835262	47835262	C	T	SNP	0|0	0|1	15|0	4|9
Chr10	47838725	47838725	A	G	SNP	0|0	0|1	44|0	4|13
Chr10	47839827	47839827	C	G	SNP	0|0	0|1	24|0	6|8
Chr10	47841559	47841559	C	T	SNP	0|0	0|1	22|0	4|11
Chr10	47841606	47841606	G	T	SNP	0|0	0|1	29|0	6|12
Chr10	47841781	47841781	T	A	SNP	0|0	0|1	32|0	1|7

Chr10	47841782	47841782	C	T	SNP	0|0	0|1	32|0	1|7
Chr10	47841803	47841803	G	A	SNP	0|0	0|1	22|0	1|7
Chr10	47841857	47841857	A	G	SNP	0|0	0|1	27|0	2|8
Chr10	47842010	47842010	C	G	SNP	0|0	0|1	42|0	4|11
Chr10	47842229	47842229	G	A	SNP	0|0	0|1	42|0	3|5
Chr10	47844235	47844235	T	A	SNP	0|0	0|1	18|0	8|10
Chr10	47844240	47844240	T	C	SNP	0|0	0|1	18|0	8|10
Chr10	47844318	47844318	A	G	SNP	0|0	0|1	27|0	8|10
Chr10	47844622	47844622	T	G	SNP	0|0	0|1	51|0	9|8
Chr10	47844639	47844639	A	C	SNP	0|0	0|1	45|0	8|7
Chr10	47844687	47844687	T	A	SNP	0|0	0|1	43|0	6|8
Chr10	47844751	47844751	G	A	SNP	0|0	0|1	42|0	3|9
Chr10	47845493	47845493	C	G	SNP	0|0	0|1	37|0	8|10
Chr10	47846241	47846241	T	A	SNP	0|0	0|1	38|0	6|10
Chr10	47848108	47848108	G	A	SNP	0|0	0|1	52|0	3|21
Chr10	47848609	47848609	A	G	SNP	0|0	0|1	14|0	4|5
Chr10	47848881	47848881	T	C	SNP	0|0	0|1	31|0	5|5
Chr10	47848975	47848975	C	T	SNP	0|0	0|1	32|1	4|9
Chr10	47848979	47848979	C	G	SNP	0|0	0|1	36|1	4|8
Chr10	47849033	47849033	C	T	SNP	0|0	0|1	39|1	1|7
Chr10	47849102	47849102	A	G	SNP	0|0	0|1	43|1	2|10
Chr10	47849117	47849117	A	G	SNP	0|0	0|1	41|1	3|10
Chr10	47850572	47850572	T	C	SNP	0|0	0|1	63|0	2|15
Chr10	47850725	47850725	C	T	SNP	0|0	0|1	32|0	5|11
Chr10	47851277	47851277	A	G	SNP	0|0	0|1	52|0	11|16
Chr10	47851585	47851585	T	C	SNP	0|0	0|1	46|0	6|20
Chr10	47851902	47851902	G	A	SNP	0|0	0|1	44|0	3|21
Chr10	47852029	47852029	T	A	SNP	0|0	0|1	37|0	7|16
Chr10	47852154	47852154	A	G	SNP	0|0	0|1	36|0	4|20
Chr10	47852598	47852598	T	C	SNP	0|0	0|1	39|0	6|11
Chr10	47852638	47852638	C	G	SNP	0|0	0|1	50|0	7|11
Chr10	47852648	47852648	G	A	SNP	0|0	0|1	52|0	8|11
Chr10	47852720	47852720	C	T	SNP	0|0	0|1	50|0	9|12
Chr10	47853101	47853101	T	C	SNP	0|0	0|1	67|1	8|9
Chr10	47853629	47853629	C	A	SNP	0|0	0|1	44|0	5|14
Chr10	47853648	47853648	C	A	SNP	0|0	0|1	47|0	6|12
Chr10	47853791	47853791	A	T	SNP	0|0	0|1	70|0	4|10
Chr10	47854529	47854529	C	T	SNP	0|0	0|1	32|0	4|12
Chr10	47854812	47854812	C	T	SNP	0|0	0|1	37|0	1|10
Chr10	47854907	47854907	T	C	SNP	0|0	0|1	43|0	3|14
Chr10	47854941	47854941	A	G	SNP	0|0	0|1	45|0	4|13
Chr10	47855011	47855011	G	A	SNP	0|0	0|1	61|0	1|8

Chr10	47855400	47855400	A	G	SNP	0|0	0|1	67|1	5|15
Chr10	47855484	47855484	G	A	SNP	0|0	0|1	61|2	6|8
Chr10	47855515	47855515	A	G	SNP	0|0	0|1	56|2	5|8
Chr10	47855548	47855548	A	G	SNP	0|0	0|1	45|1	10|7
Chr10	47855571	47855571	C	T	SNP	0|0	0|1	48|1	12|7
Chr10	47855582	47855582	G	T	SNP	0|0	0|1	48|1	12|7
Chr10	47855589	47855589	T	C	SNP	0|0	0|1	48|1	9|9
Chr10	47855614	47855614	A	G	SNP	0|0	0|1	40|0	8|10
Chr10	47855647	47855647	C	G	SNP	0|0	0|1	45|1	9|9
Chr10	47855792	47855792	G	A	SNP	0|0	0|1	52|0	9|12
Chr10	47855827	47855827	G	A	SNP	0|0	0|1	53|0	8|9
Chr10	47855873	47855873	C	T	SNP	0|0	0|1	52|0	8|12
Chr10	47855951	47855951	C	G	SNP	0|0	0|1	43|0	3|10
Chr10	47856069	47856069	A	C	SNP	0|0	0|1	34|0	2|8
Chr10	47856092	47856092	A	G	SNP	0|0	0|1	33|0	2|7
Chr10	47856355	47856355	A	T	SNP	0|0	0|1	24|0	2|8
Chr10	47856584	47856584	A	C	SNP	0|0	0|1	34|0	2|20
Chr10	47856724	47856724	A	T	SNP	0|0	0|1	51|0	6|23
Chr10	47857581	47857581	A	G	SNP	0|0	0|1	29|0	3|8
Chr10	47857766	47857766	G	A	SNP	0|0	0|1	50|0	4|8
Chr10	47857815	47857815	T	C	SNP	0|0	0|1	56|0	6|7
Chr10	47857863	47857863	C	G	SNP	0|0	0|1	54|0	6|8
Chr10	47858188	47858188	C	T	SNP	0|0	0|1	50|0	9|10
Chr10	47858853	47858853	T	C	SNP	0|0	0|1	65|0	11|17
Chr10	47858866	47858866	G	T	SNP	0|0	0|1	69|0	11|19
Chr10	47858939	47858939	T	C	SNP	0|0	0|1	85|0	9|20
Chr10	47860692	47860692	A	G	SNP	0|0	0|1	19|0	4|7
Chr10	47860734	47860734	T	G	SNP	0|0	0|1	16|0	2|6
Chr10	47860871	47860871	A	C	SNP	0|0	0|1	27|0	6|10
Chr10	47861061	47861061	G	A	SNP	0|0	0|1	39|0	3|7
Chr10	47861068	47861068	A	G	SNP	0|0	0|1	40|0	3|7
Chr10	47861209	47861209	G	C	SNP	0|0	0|1	55|0	4|8
Chr10	47861233	47861233	C	T	SNP	0|0	0|1	44|0	5|9
Chr10	47861856	47861856	A	G	SNP	0|0	0|1	65|0	10|17
Chr10	47861941	47861941	G	A	SNP	0|0	0|1	55|0	5|10
Chr10	47861944	47861944	T	A	SNP	0|0	0|1	54|0	5|10
Chr10	47862251	47862251	A	C	SNP	0|0	0|1	47|0	4|10
Chr10	47862313	47862313	A	G	SNP	0|0	0|1	50|0	4|12
Chr10	47862341	47862341	G	A	SNP	0|0	0|1	50|0	5|14
Chr10	47862461	47862461	C	G	SNP	0|0	0|1	44|0	9|18
Chr10	47862962	47862962	G	T	SNP	0|0	0|1	39|0	4|18
Chr10	47863055	47863055	G	A	SNP	0|0	0|1	38|0	4|13

Chr10	47864435	47864435	C	T	SNP	0|0	0|1	38|0	7|10
Chr10	47864552	47864552	T	A	SNP	0|0	0|1	37|0	2|12
Chr10	47864658	47864658	C	T	SNP	0|0	0|1	45|0	5|19
Chr10	47864797	47864797	C	T	SNP	0|0	0|1	44|0	3|8
Chr10	47865145	47865145	C	T	SNP	0|0	0|1	41|0	3|13
Chr10	47865248	47865248	C	T	SNP	0|0	0|1	44|0	3|9
Chr10	47865891	47865891	A	C	SNP	0|0	0|1	43|0	4|15
Chr10	47866060	47866060	A	G	SNP	0|0	0|1	63|0	3|9
Chr10	47866279	47866279	T	C	SNP	0|0	0|1	42|0	5|8
Chr10	47866368	47866368	G	A	SNP	0|0	0|1	27|1	4|15
Chr10	47867329	47867329	A	T	SNP	0|0	0|1	31|0	2|6
Chr10	47868127	47868127	G	A	SNP	0|0	0|1	41|0	5|8
Chr10	47868513	47868513	T	G	SNP	0|0	0|1	30|0	2|6
Chr10	47869618	47869618	A	G	SNP	0|0	0|1	57|0	1|12
Chr10	47869872	47869872	C	T	SNP	0|0	0|1	39|0	2|12
Chr10	47871389	47871389	A	G	SNP	0|0	0|1	38|0	2|14
Chr10	47871903	47871903	C	T	SNP	0|0	0|1	44|0	4|6
Chr10	47871997	47871997	T	C	SNP	0|0	0|1	46|0	4|9
Chr10	47872310	47872310	G	A	SNP	0|0	0|1	44|0	7|17
Chr10	47872447	47872447	A	G	SNP	0|0	0|1	57|0	5|20
Chr10	47872597	47872597	C	T	SNP	0|0	0|1	63|0	6|23
Chr10	47873002	47873002	A	G	SNP	0|0	0|1	54|0	6|10
Chr10	47873105	47873105	G	T	SNP	0|0	0|1	55|1	2|17
Chr10	47873231	47873231	T	C	SNP	0|0	0|1	65|1	4|14
Chr10	47873270	47873270	T	C	SNP	0|0	0|1	69|0	3|13
Chr10	47873272	47873272	T	C	SNP	0|0	0|1	69|0	3|13
Chr10	47874335	47874335	T	G	SNP	0|0	0|1	20|0	3|8
Chr10	47876087	47876087	C	T	SNP	0|0	0|1	57|0	6|14
Chr10	47877454	47877454	C	T	SNP	0|0	0|1	26|0	5|9
Chr10	47878949	47878949	G	T	SNP	0|0	1|1	30|0	0|9
Chr10	47881732	47881732	A	C	SNP	0|0	0|1	54|0	4|11
Chr10	47881813	47881813	T	G	SNP	0|0	0|1	34|0	3|12
Chr10	47884238	47884238	A	T	SNP	0|0	0|1	11|0	3|5
Chr10	47885081	47885081	A	G	SNP	0|0	0|1	10|0	6|6
Chr10	47885473	47885473	C	T	SNP	0|0	0|1	8|0	3|5
Chr10	47885789	47885789	T	A	SNP	0|0	0|1	20|0	3|5
Chr10	47886118	47886118	G	A	SNP	0|0	0|1	34|0	4|5
Chr10	47886249	47886249	G	T	SNP	0|0	0|1	49|0	5|8
Chr10	47888620	47888620	G	A	SNP	0|0	0|1	25|0	4|6
Chr10	47890256	47890256	G	A	SNP	0|0	1|1	42|0	0|9
Chr10	47890374	47890374	T	C	SNP	0|0	0|1	49|1	1|10
Chr10	47892507	47892507	G	T	SNP	0|0	0|1	29|0	3|9

Chr10	47894676	47894676	T	A	SNP	0|0	0|1	51|0	6|14
Chr10	47894680	47894680	C	A	SNP	0|0	0|1	52|0	6|14
Chr10	47896398	47896398	C	A	SNP	0|0	0|1	30|1	4|7
Chr10	47897262	47897262	A	C	SNP	0|0	0|1	33|0	3|5
Chr10	47897351	47897351	T	C	SNP	0|0	0|1	38|0	4|4
Chr10	47902453	47902453	G	A	SNP	0|0	0|1	63|0	5|18
Chr10	47909564	47909564	T	C	SNP	0|0	0|1	71|0	5|17
Chr10	47911215	47911215	T	C	SNP	0|0	0|1	14|0	1|8
Chr10	47912561	47912561	G	T	SNP	0|0	0|1	26|0	1|12
Chr10	47912619	47912619	G	A	SNP	0|0	0|1	18|0	1|10
Chr10	47913718	47913718	A	T	SNP	0|0	0|1	37|0	3|14
Chr10	47914256	47914256	A	T	SNP	0|0	0|1	51|0	2|18
Chr10	47914452	47914452	T	A	SNP	0|0	0|1	53|1	3|15
Chr10	47914515	47914515	C	T	SNP	0|0	1|1	57|1	0|17
Chr10	47914528	47914528	C	T	SNP	0|0	1|1	59|1	0|18
Chr10	47914672	47914672	A	C	SNP	0|0	0|1	59|3	4|11
Chr10	47914809	47914809	G	T	SNP	0|0	0|1	62|0	5|18
Chr10	47914887	47914887	C	A	SNP	0|0	0|1	60|0	5|19
Chr10	47914897	47914897	T	G	SNP	0|0	0|1	59|0	5|18
Chr10	47914986	47914986	C	A	SNP	0|0	0|1	52|0	8|15
Chr10	47914999	47914999	G	T	SNP	0|0	0|1	50|0	8|13
Chr10	47915108	47915108	T	C	SNP	0|0	0|1	77|0	5|9
Chr10	47915280	47915280	G	A	SNP	0|0	0|1	49|0	7|12
Chr10	47915404	47915404	T	G	SNP	0|0	0|1	75|0	9|19
Chr10	47915591	47915591	C	T	SNP	0|0	0|1	44|0	12|15
Chr10	47915748	47915748	C	A	SNP	0|0	0|1	29|0	3|15
Chr10	47916538	47916538	G	C	SNP	0|0	0|1	33|0	5|12
Chr10	47918346	47918346	G	T	SNP	0|0	0|1	41|0	6|15
Chr10	47918357	47918357	C	A	SNP	0|0	0|1	37|0	6|18
Chr10	47919875	47919875	T	C	SNP	0|0	0|1	48|1	4|9
Chr10	47919995	47919995	A	G	SNP	0|0	0|1	51|0	5|13
Chr10	47920059	47920059	G	A	SNP	0|0	0|1	63|0	5|13
Chr10	47920291	47920291	C	T	SNP	0|0	0|1	44|0	5|13
Chr10	47920602	47920602	A	G	SNP	0|0	0|1	19|0	2|6
Chr10	47922672	47922672	G	A	SNP	0|0	0|1	65|0	8|18
Chr10	47922971	47922971	C	T	SNP	0|0	0|1	36|0	9|6
Chr10	47924243	47924243	G	A	SNP	0|0	0|1	76|0	6|21
Chr10	47924842	47924842	T	C	SNP	0|0	0|1	61|1	5|5
Chr10	47924986	47924986	A	C	SNP	0|0	0|1	44|0	5|8
Chr10	47925184	47925184	A	T	SNP	0|0	0|1	63|0	15|7
Chr10	47925782	47925782	C	A	SNP	0|0	0|1	66|1	5|10
Chr10	47925845	47925845	C	T	SNP	0|0	0|1	66|0	4|15

Chr10	47926476	47926476	G	A	SNP	0|0	0|1	49|0	6|11
Chr10	47927394	47927394	A	T	SNP	0|0	0|1	49|0	4|10
Chr10	47927405	47927405	C	T	SNP	0|0	0|1	48|0	4|9
Chr10	47928200	47928200	T	C	SNP	0|0	0|1	23|0	5|8
Chr10	47928285	47928285	A	G	SNP	0|0	0|1	38|0	3|5
Chr10	47929384	47929384	G	C	SNP	0|0	0|1	53|1	4|10
Chr10	47929839	47929839	A	G	SNP	0|0	0|1	53|0	9|11
Chr10	47930036	47930036	C	T	SNP	0|0	0|1	62|1	7|18
Chr10	47931409	47931409	G	A	SNP	0|0	0|1	55|0	3|17
Chr10	47931708	47931708	C	G	SNP	0|0	0|1	63|1	8|15
Chr10	47931991	47931991	T	C	SNP	0|0	0|1	62|0	9|11
Chr10	47932566	47932566	T	A	SNP	0|0	0|1	29|0	1|11
Chr10	47932630	47932630	G	T	SNP	0|0	0|1	41|0	2|12
Chr10	47932728	47932728	C	T	SNP	0|0	0|1	60|0	1|9
Chr10	47933238	47933238	T	C	SNP	0|0	0|1	27|0	1|11
Chr10	47933310	47933310	G	A	SNP	0|0	0|1	28|0	1|7
Chr10	47934575	47934575	T	A	SNP	0|0	0|1	24|0	8|6
Chr10	47935195	47935195	C	T	SNP	0|0	0|1	25|0	5|5
Chr10	47936705	47936705	G	T	SNP	0|0	0|1	43|0	7|17
Chr10	47939686	47939686	T	A	SNP	0|0	0|1	30|0	4|10
Chr10	47939703	47939703	C	A	SNP	0|0	0|1	32|0	4|13
Chr10	47940932	47940932	A	G	SNP	0|0	0|1	53|0	4|18
Chr10	47942368	47942368	G	A	SNP	0|0	0|1	56|0	5|15
Chr10	47942570	47942570	T	C	SNP	0|0	0|1	58|1	5|14
Chr10	47942933	47942933	T	C	SNP	0|0	0|1	47|0	6|13
Chr10	47943082	47943082	G	C	SNP	0|0	0|1	35|0	5|18
Chr10	47944350	47944350	T	C	SNP	0|0	0|1	59|2	6|23
Chr10	47949363	47949363	C	T	SNP	0|0	1|1	18|0	0|9
Chr10	47949553	47949553	T	C	SNP	0|0	0|1	41|0	2|10
Chr10	47950173	47950173	T	A	SNP	0|0	0|1	49|0	2|11
Chr10	47950306	47950306	A	G	SNP	0|0	0|1	44|0	3|12
Chr10	47950432	47950432	C	T	SNP	0|0	0|1	32|0	1|7
Chr10	47950860	47950860	T	A	SNP	0|0	0|1	27|2	3|7
Chr10	47954816	47954816	C	A	SNP	0|0	0|1	53|0	4|14
Chr10	47954954	47954954	G	A	SNP	0|0	0|1	57|0	1|16
Chr10	47955088	47955088	T	G	SNP	0|0	0|1	41|0	7|8
Chr10	47955089	47955089	T	C	SNP	0|0	0|1	41|0	8|8
Chr10	47955090	47955090	T	C	SNP	0|0	0|1	42|0	8|8
Chr10	47957907	47957907	A	T	SNP	0|0	0|1	63|0	6|8
Chr10	47958116	47958116	T	C	SNP	0|0	0|1	60|0	7|14
Chr10	47958731	47958731	G	A	SNP	0|0	0|1	39|0	3|9
Chr10	47958771	47958771	C	T	SNP	0|0	0|1	32|0	7|13

Chr10	47960928	47960928	G	T	SNP	0|0	0|1	36|1	8|7
Chr10	47961242	47961242	T	A	SNP	0|0	0|1	48|2	4|17
Chr10	47961404	47961404	A	G	SNP	0|0	0|1	49|0	4|7
Chr10	47961621	47961621	A	T	SNP	0|0	0|1	21|0	10|6
Chr10	47961962	47961962	A	G	SNP	0|0	0|1	37|2	4|5
Chr10	47967393	47967393	C	T	SNP	0|0	0|1	25|0	4|16
Chr10	47967423	47967423	G	A	SNP	0|0	0|1	27|0	5|18
Chr10	47967458	47967458	G	T	SNP	0|0	0|1	22|0	4|17
Chr10	47967470	47967470	T	A	SNP	0|0	0|1	24|0	4|17
Chr10	47967496	47967496	C	G	SNP	0|0	0|1	24|0	5|13
Chr10	47967534	47967534	A	C	SNP	0|0	0|1	18|0	3|7
Chr10	47967882	47967882	T	C	SNP	0|0	0|1	19|0	3|5
Chr10	47967900	47967900	C	T	SNP	0|0	0|1	17|0	5|5
Chr10	47967909	47967909	T	C	SNP	0|0	0|1	17|0	5|5
Chr10	47970006	47970006	G	A	SNP	0|0	0|1	75|0	5|16
Chr10	47970966	47970966	T	G	SNP	0|0	0|1	34|0	3|12
Chr10	47971099	47971099	T	C	SNP	0|0	0|1	50|0	8|4
Chr10	47973530	47973530	G	C	SNP	0|0	0|1	59|0	3|17
Chr10	47973605	47973605	T	C	SNP	0|0	0|1	60|0	5|13
Chr10	47973723	47973723	G	A	SNP	0|0	0|1	53|0	4|9
Chr10	47973731	47973731	G	C	SNP	0|0	0|1	55|0	4|9
Chr10	47973977	47973977	A	G	SNP	0|0	0|1	71|0	4|9
Chr10	47974244	47974244	T	G	SNP	0|0	0|1	55|1	7|11
Chr10	47974294	47974294	G	A	SNP	0|0	0|1	46|1	6|13
Chr10	47974422	47974422	G	A	SNP	0|0	0|1	33|0	3|16
Chr10	47974442	47974442	C	T	SNP	0|0	0|1	33|0	3|15
Chr10	47974554	47974554	C	A	SNP	0|0	1|1	54|0	1|24
Chr10	47974701	47974701	A	T	SNP	0|0	0|1	40|0	4|10
Chr10	47974727	47974727	A	G	SNP	0|0	0|1	38|0	5|11
Chr10	47974882	47974882	A	T	SNP	0|0	0|1	61|0	3|13
Chr10	47974928	47974928	G	A	SNP	0|0	0|1	56|0	2|13
Chr10	47975056	47975056	G	A	SNP	0|0	0|1	42|0	6|15
Chr10	47975105	47975105	T	C	SNP	0|0	0|1	48|0	5|12
Chr10	47975185	47975185	T	G	SNP	0|0	0|1	49|1	4|17
Chr10	47975462	47975462	G	C	SNP	0|0	0|1	57|0	1|17
Chr10	47975872	47975872	T	A	SNP	0|0	0|1	52|0	5|16
Chr10	47975874	47975874	A	G	SNP	0|0	0|1	53|0	7|17
Chr10	47975889	47975889	A	C	SNP	0|0	0|1	51|0	8|14
Chr10	47976053	47976053	G	A	SNP	0|0	1|1	49|0	0|26
Chr10	47976075	47976075	A	T	SNP	0|0	1|1	51|0	0|22
Chr10	47976136	47976136	A	T	SNP	0|0	0|1	35|0	2|18
Chr10	47976549	47976549	T	C	SNP	0|0	1|1	18|0	1|16

Chr10	47976619	47976619	T	A	SNP	0|0	1|1	20|0	0|13
Chr10	47976757	47976757	C	G	SNP	0|0	0|1	21|0	1|11
Chr10	47976821	47976821	A	T	SNP	0|0	1|1	20|0	0|13
Chr10	47977013	47977013	G	A	SNP	0|0	0|1	24|0	6|6
Chr10	47977162	47977162	A	T	SNP	0|0	0|1	21|0	1|7
Chr10	47979670	47979670	C	T	SNP	0|0	0|1	26|0	3|5
Chr10	47981044	47981044	T	C	SNP	0|0	0|1	57|0	10|21
Chr10	47981671	47981671	C	T	SNP	0|0	0|1	60|0	5|5
Chr10	47981709	47981709	T	A	SNP	0|0	0|1	74|0	6|5
Chr10	47982221	47982221	C	A	SNP	0|0	0|1	33|0	1|13
Chr10	47982280	47982280	A	C	SNP	0|0	0|1	38|0	2|11
Chr10	47983164	47983164	T	C	SNP	0|0	0|1	32|0	7|9
Chr10	47984157	47984157	C	G	SNP	0|0	1|1	28|0	0|8
Chr10	47984369	47984369	C	T	SNP	0|0	1|1	43|2	0|10
Chr10	47984691	47984691	C	T	SNP	0|0	0|1	30|0	2|14
Chr10	47984820	47984820	T	C	SNP	0|0	0|1	46|0	5|9
Chr10	47984860	47984860	C	G	SNP	0|0	0|1	49|0	6|7
Chr10	47984948	47984948	T	C	SNP	0|0	0|1	44|0	7|8
Chr10	47984965	47984965	G	A	SNP	0|0	0|1	41|0	7|10
Chr10	47984985	47984985	T	C	SNP	0|0	0|1	40|0	6|13
Chr10	47985221	47985221	C	A	SNP	0|0	0|1	33|0	7|17
Chr10	47985630	47985630	T	A	SNP	0|0	0|1	61|0	4|28
Chr10	47985897	47985897	T	C	SNP	0|0	0|1	71|0	10|21
Chr10	47986091	47986091	C	T	SNP	0|0	0|1	55|0	5|14
Chr10	47986092	47986092	G	A	SNP	0|0	0|1	57|0	5|14
Chr10	47986106	47986106	T	G	SNP	0|0	0|1	56|0	5|14
Chr10	47986108	47986108	A	C	SNP	0|0	0|1	56|0	5|16
Chr10	47986150	47986150	T	C	SNP	0|0	0|1	58|0	5|13
Chr10	47986280	47986280	T	C	SNP	0|0	0|1	79|0	5|10
Chr10	47986391	47986391	A	G	SNP	0|0	0|1	88|0	11|9
Chr10	47986516	47986516	C	T	SNP	0|0	0|1	53|1	6|11
Chr10	47986689	47986689	A	G	SNP	0|0	0|1	62|0	12|12
Chr10	47986838	47986838	A	T	SNP	0|0	0|1	39|1	5|13
Chr10	47987421	47987421	T	C	SNP	0|0	0|1	10|0	2|7
Chr10	47987485	47987485	T	G	SNP	0|0	0|1	11|0	3|8
Chr10	47987877	47987877	G	A	SNP	0|0	0|1	36|2	4|10
Chr10	47988827	47988827	T	G	SNP	0|0	0|1	43|0	5|22
Chr10	47988919	47988919	C	A	SNP	0|0	0|1	46|0	4|19
Chr10	47989096	47989096	C	T	SNP	0|0	0|1	43|0	5|11
Chr10	47990087	47990087	G	A	SNP	0|0	0|1	60|0	6|6
Chr10	47990633	47990633	G	T	SNP	0|0	1|1	61|0	1|20
Chr10	47990761	47990761	T	C	SNP	0|0	0|1	57|0	5|11

Chr10	47990810	47990810	T	C	SNP	0|0	0|1	44|0	3|12
Chr10	47990938	47990938	A	C	SNP	0|0	0|1	53|0	2|16
Chr10	47991075	47991075	T	A	SNP	0|0	0|1	60|0	11|8
Chr10	47991430	47991430	T	A	SNP	0|0	0|1	27|0	4|4
Chr10	47991440	47991440	A	G	SNP	0|0	0|1	21|0	4|4
Chr10	47992204	47992204	T	C	SNP	0|0	0|1	11|0	2|10
Chr10	47992323	47992323	A	G	SNP	0|0	0|1	30|0	4|8
Chr10	47992499	47992499	T	C	SNP	0|0	0|1	17|0	1|8
Chr10	47993605	47993605	G	C	SNP	0|0	0|1	73|2	6|11
Chr10	47994867	47994867	A	C	SNP	0|0	0|1	47|0	6|11
Chr10	47995308	47995308	G	A	SNP	0|0	0|1	93|0	7|18
Chr10	47995995	47995995	T	A	SNP	0|0	0|1	74|0	6|17
Chr10	47996038	47996038	C	A	SNP	0|0	0|1	79|0	8|16
Chr10	47996498	47996498	T	A	SNP	0|0	0|1	83|3	6|15
Chr10	47997831	47997831	C	T	SNP	0|0	0|1	18|0	3|5
Chr10	47997856	47997856	C	T	SNP	0|0	0|1	27|0	3|5
Chr10	47997959	47997959	A	C	SNP	0|0	0|1	45|0	2|9
Chr10	47998666	47998666	T	A	SNP	0|0	0|1	67|1	6|15
Chr10	47998743	47998743	G	A	SNP	0|0	0|1	67|5	10|9
Chr10	48000740	48000740	C	A	SNP	0|0	0|1	66|3	8|7
Chr10	48000787	48000787	A	G	SNP	0|0	0|1	51|0	10|8
Chr10	48002571	48002571	T	A	SNP	0|0	0|1	39|0	3|10
Chr10	48002580	48002580	A	G	SNP	0|0	0|1	32|0	3|9
Chr10	48002591	48002591	C	A	SNP	0|0	0|1	31|0	3|9
Chr10	48002621	48002621	T	C	SNP	0|0	0|1	28|0	3|7
Chr10	48002736	48002736	C	A	SNP	0|0	1|1	15|0	0|9
Chr10	48003851	48003851	T	A	SNP	0|0	0|1	38|0	8|6
Chr10	48003853	48003853	A	C	SNP	0|0	0|1	38|0	8|6
Chr10	48004161	48004161	A	T	SNP	0|0	0|1	18|0	4|7
Chr10	48004174	48004174	C	G	SNP	0|0	0|1	16|0	4|7
Chr10	48004609	48004609	G	A	SNP	0|0	0|1	32|1	5|8
Chr10	48004742	48004742	C	T	SNP	0|0	0|1	45|1	2|10
Chr10	48004795	48004795	T	G	SNP	0|0	0|1	38|1	2|10
Chr10	48004861	48004861	C	A	SNP	0|0	1|1	28|1	0|10
Chr10	48005464	48005464	A	C	SNP	0|0	0|1	49|0	6|15
Chr10	48005478	48005478	T	A	SNP	0|0	0|1	46|0	6|18
Chr10	48005647	48005647	C	A	SNP	0|0	0|1	76|0	11|25
Chr10	48005741	48005741	G	A	SNP	0|0	0|1	59|1	11|15
Chr10	48006660	48006660	T	A	SNP	0|0	0|1	35|0	12|7
Chr10	48006966	48006966	A	G	SNP	0|0	0|1	59|0	8|11
Chr10	48007027	48007027	A	G	SNP	0|0	0|1	64|0	4|11
Chr10	48008937	48008937	T	G	SNP	0|0	0|1	63|0	9|13

Chr10	48009970	48009970	G	A	SNP	0|0	0|1	41|2	2|10
Chr10	48010213	48010213	G	C	SNP	0|0	0|1	45|1	4|8
Chr10	48010278	48010278	C	G	SNP	0|0	0|1	33|0	7|5
Chr10	48011117	48011117	T	G	SNP	0|0	0|1	13|0	2|6
Chr10	48011191	48011191	A	C	SNP	0|0	1|1	11|0	0|11
Chr10	48011239	48011239	T	G	SNP	0|0	1|1	14|0	0|11
Chr10	48011339	48011339	A	T	SNP	0|0	0|1	10|0	2|7
Chr10	48012018	48012018	T	A	SNP	0|0	0|1	46|2	6|11
Chr10	48012074	48012074	G	T	SNP	0|0	0|1	34|0	5|12
Chr10	48012886	48012886	G	A	SNP	0|0	0|1	75|0	5|10
Chr10	48012917	48012917	A	C	SNP	0|0	0|1	70|0	4|9
Chr10	48013782	48013782	G	A	SNP	0|0	0|1	42|0	1|12
Chr10	48013787	48013787	A	C	SNP	0|0	0|1	44|0	1|12
Chr10	48013807	48013807	C	A	SNP	0|0	0|1	37|0	1|16
Chr10	48013945	48013945	C	T	SNP	0|0	0|1	65|0	9|12
Chr10	48013984	48013984	A	G	SNP	0|0	0|1	66|6	10|9
Chr10	48014288	48014288	C	G	SNP	0|0	0|1	41|0	8|13
Chr10	48014458	48014458	G	A	SNP	0|0	0|1	40|0	5|11
Chr10	48014760	48014760	T	C	SNP	0|0	0|1	47|0	5|7
Chr10	48015123	48015123	T	C	SNP	0|0	0|1	43|1	6|16
Chr10	48015666	48015666	C	T	SNP	0|0	0|1	56|0	5|20
Chr10	48015938	48015938	G	T	SNP	0|0	0|1	55|0	5|17
Chr10	48016464	48016464	A	G	SNP	0|0	0|1	39|0	3|7
Chr10	48016489	48016489	G	A	SNP	0|0	0|1	45|0	3|10
Chr10	48016522	48016522	G	A	SNP	0|0	0|1	46|0	4|13
Chr10	48017177	48017177	A	T	SNP	0|0	0|1	61|2	5|8
Chr10	48017424	48017424	G	A	SNP	0|0	0|1	64|2	10|14
Chr10	48017446	48017446	T	C	SNP	0|0	0|1	66|2	9|17
Chr10	48017555	48017555	G	A	SNP	0|0	0|1	66|0	5|16
Chr10	48018227	48018227	C	T	SNP	0|0	0|1	47|2	3|10
Chr10	48018233	48018233	C	A	SNP	0|0	0|1	47|2	3|10
Chr10	48018247	48018247	G	A	SNP	0|0	0|1	45|2	3|10
Chr10	48018944	48018944	T	A	SNP	0|0	0|1	19|0	1|14
Chr10	48019000	48019000	C	T	SNP	0|0	1|1	16|1	0|10
Chr10	48019626	48019626	G	A	SNP	0|0	0|1	43|1	4|7
Chr10	48021587	48021587	A	T	SNP	0|0	1|1	26|0	0|9
Chr10	48021601	48021601	C	T	SNP	0|0	1|1	27|0	0|8
Chr10	48023525	48023525	A	C	SNP	0|0	1|1	15|0	0|10
Chr10	48023563	48023563	T	C	SNP	0|0	1|1	17|0	0|9
Chr10	48024059	48024059	T	C	SNP	0|0	0|1	29|1	6|3
Chr10	48024120	48024120	T	A	SNP	0|0	0|1	30|0	5|4
Chr10	48024453	48024453	A	C	SNP	0|0	0|1	30|0	3|5

Chr10	48024467	48024467	C	T	SNP	0|0	0|1	33|0	4|6
Chr10	48024468	48024468	G	T	SNP	0|0	0|1	33|0	4|6
Chr10	48024494	48024494	G	A	SNP	0|0	0|1	34|0	2|6
Chr10	48024503	48024503	A	G	SNP	0|0	0|1	34|0	2|6
Chr10	48024508	48024508	T	C	SNP	0|0	0|1	34|0	2|6
Chr10	48024514	48024514	G	T	SNP	0|0	0|1	35|0	2|6
Chr10	48024519	48024519	T	C	SNP	0|0	0|1	37|0	2|7
Chr10	48024520	48024520	C	T	SNP	0|0	0|1	37|0	2|7
Chr10	48024536	48024536	C	T	SNP	0|0	0|1	40|0	2|6
Chr10	48024561	48024561	C	T	SNP	0|0	0|1	39|0	2|6
Chr10	48024587	48024587	C	T	SNP	0|0	0|1	46|0	2|6
Chr10	48024590	48024590	A	G	SNP	0|0	0|1	47|0	2|6
Chr10	48024596	48024596	T	C	SNP	0|0	0|1	52|0	3|6
Chr10	48024598	48024598	A	G	SNP	0|0	0|1	52|0	3|6
Chr10	48024599	48024599	C	T	SNP	0|0	0|1	52|0	3|6
Chr10	48024611	48024611	T	C	SNP	0|0	0|1	52|0	4|5
Chr10	48024612	48024612	G	A	SNP	0|0	0|1	52|0	4|5
Chr10	48024618	48024618	T	G	SNP	0|0	0|1	54|0	3|5
Chr10	48024631	48024631	G	A	SNP	0|0	0|1	58|0	3|5
Chr10	48024636	48024636	T	C	SNP	0|0	0|1	56|0	3|5
Chr10	48024726	48024726	G	A	SNP	0|0	0|1	42|0	4|5
Chr10	48024737	48024737	T	C	SNP	0|0	0|1	43|0	4|5
Chr10	48024738	48024738	T	C	SNP	0|0	0|1	43|0	4|5
Chr10	48024750	48024750	A	G	SNP	0|0	0|1	43|0	3|5
Chr10	48024755	48024755	A	G	SNP	0|0	0|1	42|0	3|5
Chr10	48024760	48024760	C	A	SNP	0|0	0|1	42|0	3|5
Chr10	48024761	48024761	G	A	SNP	0|0	0|1	42|0	3|5
Chr10	48024769	48024769	C	T	SNP	0|0	0|1	36|0	3|5
Chr10	48024779	48024779	T	C	SNP	0|0	0|1	39|0	3|5
Chr10	48024832	48024832	C	T	SNP	0|0	0|1	37|0	1|7
Chr10	48024835	48024835	A	G	SNP	0|0	0|1	37|0	1|7
Chr10	48024840	48024840	T	C	SNP	0|0	0|1	35|0	1|7
Chr10	48024843	48024843	T	C	SNP	0|0	0|1	35|0	1|7
Chr10	48024898	48024898	G	A	SNP	0|0	0|1	17|0	3|5
Chr10	48025293	48025293	A	T	SNP	0|0	0|1	30|1	3|8
Chr10	48028290	48028290	A	G	SNP	0|0	0|1	48|0	3|14
Chr10	48028489	48028489	A	G	SNP	0|0	0|1	34|0	5|7
Chr10	48028509	48028509	C	T	SNP	0|0	0|1	35|0	4|6
Chr10	48028647	48028647	G	C	SNP	0|0	0|1	22|0	4|9
Chr10	48028746	48028746	A	G	SNP	0|0	0|1	27|0	2|8
Chr10	48028760	48028760	C	G	SNP	0|0	0|1	25|0	2|8
Chr10	48028768	48028768	C	T	SNP	0|0	0|1	27|0	1|8

Chr10	48028773	48028773	A	G	SNP	0|0	0|1	27|0	1|8
Chr10	48029773	48029773	A	G	SNP	0|0	0|1	23|1	5|10
Chr10	48029809	48029809	A	G	SNP	0|0	0|1	24|1	6|11
Chr10	48029843	48029843	C	A	SNP	0|0	0|1	25|1	5|10
Chr10	48029921	48029921	A	G	SNP	0|1	0|1	32|3	6|8
Chr10	48029930	48029930	A	T	SNP	0|0	0|1	35|2	5|7
Chr10	48029989	48029989	T	C	SNP	0|0	0|1	40|2	6|4
Chr10	48030033	48030033	C	T	SNP	0|0	0|1	40|1	3|6
Chr10	48031220	48031220	T	A	SNP	0|0	0|1	43|0	7|15
Chr10	48031323	48031323	G	A	SNP	0|0	0|1	14|0	4|12
Chr10	48031337	48031337	G	A	SNP	0|0	0|1	16|0	4|11
Chr10	48031344	48031344	C	A	SNP	0|0	0|1	17|0	4|11
Chr10	48031357	48031357	G	A	SNP	0|0	0|1	21|0	4|9
Chr10	48031365	48031365	A	G	SNP	0|0	0|1	26|0	4|7
Chr10	48031385	48031385	G	A	SNP	0|0	0|1	25|0	2|9
Chr10	48031396	48031396	G	A	SNP	0|0	1|1	25|0	0|10
Chr10	48031407	48031407	A	C	SNP	0|0	1|1	27|0	0|10
Chr10	48031423	48031423	C	T	SNP	0|0	1|1	21|0	0|9
Chr10	48031458	48031458	A	G	SNP	0|0	0|1	27|0	1|8
Chr10	48031476	48031476	G	A	SNP	0|0	0|1	34|0	1|8
Chr10	48031507	48031507	C	T	SNP	0|0	0|1	32|0	1|7
Chr10	48031512	48031512	C	G	SNP	0|0	0|1	27|0	1|7
Chr10	48032196	48032196	C	A	SNP	0|0	0|1	45|1	8|13
Chr10	48032336	48032336	T	C	SNP	0|1	0|1	41|3	3|10
Chr10	48032341	48032341	G	T	SNP	0|1	0|1	39|2	4|10
Chr10	48032750	48032750	T	A	SNP	0|0	0|1	33|1	3|12
Chr10	48032815	48032815	A	C	SNP	0|0	0|1	21|1	5|14
Chr10	48032836	48032836	A	T	SNP	0|0	0|1	20|0	3|12
Chr10	48033048	48033048	A	G	SNP	0|0	0|1	26|0	5|9
Chr10	48033136	48033136	G	T	SNP	0|0	0|1	31|0	6|9
Chr10	48034731	48034731	A	G	SNP	0|0	0|1	60|0	6|12
Chr10	48037028	48037028	A	C	SNP	0|0	0|1	14|0	2|7
Chr10	48037861	48037861	T	G	SNP	0|0	0|1	25|1	4|11
Chr10	48037874	48037874	T	C	SNP	0|0	0|1	28|1	4|11
Chr10	48037884	48037884	A	T	SNP	0|0	0|1	33|1	4|11
Chr10	48037887	48037887	T	C	SNP	0|0	0|1	33|1	4|11
Chr10	48037901	48037901	C	T	SNP	0|0	0|1	34|1	3|9
Chr10	48037932	48037932	C	A	SNP	0|0	0|1	35|0	3|6
Chr10	48037958	48037958	T	C	SNP	0|0	0|1	36|0	4|6
Chr10	48037960	48037960	C	T	SNP	0|0	0|1	36|0	4|6
Chr10	48037966	48037966	C	T	SNP	0|0	0|1	36|0	3|8
Chr10	48037975	48037975	T	C	SNP	0|0	0|1	34|0	3|6

Chr10	48038002	48038002	A	G	SNP	0|0	0|1	28|0	3|5
Chr10	48038060	48038060	T	C	SNP	0|0	0|1	40|0	7|6
Chr10	48038062	48038062	T	C	SNP	0|0	0|1	41|0	7|6
Chr10	48038072	48038072	C	A	SNP	0|0	0|1	45|0	7|6
Chr10	48038100	48038100	A	T	SNP	0|0	0|1	51|0	5|6
Chr10	48038738	48038738	G	A	SNP	0|1	0|1	27|2	6|7
Chr10	48038791	48038791	A	G	SNP	0|0	0|1	32|1	6|5
Chr10	48038809	48038809	A	C	SNP	0|0	0|1	38|1	6|3
Chr10	48039507	48039507	T	A	SNP	0|0	0|1	8|0	5|3
Chr10	48040421	48040421	A	C	SNP	0|0	0|1	31|2	4|14
Chr10	48040772	48040772	A	T	SNP	0|0	0|1	29|1	7|9
Chr10	48040773	48040773	A	T	SNP	0|0	0|1	29|1	7|8
Chr10	48041973	48041973	G	C	SNP	0|0	0|1	39|0	8|6
Chr10	48044223	48044223	A	G	SNP	0|0	0|1	33|0	1|13
Chr10	48044316	48044316	C	T	SNP	0|0	0|1	20|0	2|18
Chr10	48044353	48044353	T	C	SNP	0|0	0|1	18|0	3|8
Chr10	48044369	48044369	G	C	SNP	0|0	0|1	17|0	2|7
Chr10	48044562	48044562	C	A	SNP	0|1	0|1	9|2	2|6
Chr10	48044570	48044570	A	G	SNP	0|1	0|1	10|2	3|6
Chr10	48044598	48044598	C	A	SNP	0|1	0|1	17|2	3|7
Chr10	48044651	48044651	T	C	SNP	0|0	0|1	21|1	3|7
Chr10	48046332	48046332	G	C	SNP	0|0	0|1	46|1	6|22
Chr10	48046336	48046336	A	C	SNP	0|0	0|1	45|1	5|22
Chr10	48046593	48046593	A	C	SNP	0|0	0|1	39|0	7|11
Chr10	48046644	48046644	C	A	SNP	0|0	0|1	27|0	1|8
Chr10	48046664	48046664	G	C	SNP	0|0	0|1	27|0	3|7
Chr10	48046692	48046692	T	A	SNP	0|0	0|1	21|0	2|8
Chr10	48046905	48046905	C	A	SNP	0|0	0|1	24|0	5|4
Chr10	48047199	48047199	G	A	SNP	0|0	0|1	27|0	4|7
Chr10	48047954	48047954	G	A	SNP	0|0	0|1	54|1	10|17
Chr10	48048004	48048004	A	C	SNP	0|0	0|1	50|1	9|12
Chr10	48048149	48048149	T	G	SNP	0|0	0|1	44|1	10|15
Chr10	48048177	48048177	G	A	SNP	0|0	0|1	48|1	10|15
Chr10	48049214	48049214	C	T	SNP	0|0	0|1	45|2	4|5
Chr10	48049218	48049218	G	A	SNP	0|0	0|1	47|2	4|5
Chr10	48049617	48049617	A	G	SNP	0|0	0|1	27|0	4|8
Chr10	48049915	48049915	G	A	SNP	0|0	0|1	66|2	5|13
Chr10	48050029	48050029	T	C	SNP	0|1	0|1	67|10	9|16
Chr10	48050388	48050388	C	T	SNP	0|0	0|1	65|3	13|14
Chr10	48050435	48050435	C	T	SNP	0|0	0|1	50|3	8|10
Chr10	48050644	48050644	G	T	SNP	0|0	0|1	50|1	2|25
Chr10	48050868	48050868	A	G	SNP	0|0	0|1	46|2	4|12

Chr10	48050891	48050891	C	T	SNP	0|0	0|1	48|1	4|14
Chr10	48051210	48051210	C	T	SNP	0|0	0|1	39|2	4|10
Chr10	48053184	48053184	T	A	SNP	0|0	0|1	35|1	6|15
Chr10	48053371	48053371	G	C	SNP	0|0	0|1	41|2	7|10
Chr10	48054483	48054483	C	T	SNP	0|1	0|1	40|4	6|6
Chr10	48058564	48058564	T	G	SNP	0|0	0|1	26|0	1|15
Chr10	48058659	48058659	G	A	SNP	0|0	0|1	44|1	3|20
Chr10	48059149	48059149	T	C	SNP	0|0	0|1	62|0	7|12
Chr10	48060866	48060866	A	T	SNP	0|0	0|1	71|0	7|9
Chr10	48061522	48061522	A	T	SNP	0|0	0|1	26|1	3|11
Chr10	48063242	48063242	C	T	SNP	0|0	0|1	20|0	1|7
Chr10	48066594	48066594	C	T	SNP	0|0	0|1	34|1	2|9
Chr10	48066679	48066679	C	T	SNP	0|0	0|1	48|0	4|8
Chr10	48066735	48066735	C	T	SNP	0|0	0|1	54|0	4|12
Chr10	48066784	48066784	G	A	SNP	0|0	0|1	83|1	6|12
Chr10	48066870	48066870	G	A	SNP	0|0	0|1	65|1	2|7
Chr10	48067079	48067079	C	T	SNP	0|0	0|1	43|0	6|11
Chr10	48068420	48068420	G	A	SNP	0|0	0|1	35|2	1|9
Chr10	48068429	48068429	G	A	SNP	0|0	0|1	37|2	1|9
Chr10	48068443	48068443	C	T	SNP	0|0	0|1	41|1	1|9
Chr10	48068642	48068642	T	C	SNP	0|0	0|1	35|0	5|6
Chr10	48068887	48068887	T	A	SNP	0|1	0|1	65|9	8|11
Chr10	48069243	48069243	A	C	SNP	0|0	0|1	78|2	9|21
Chr10	48069658	48069658	T	C	SNP	0|0	0|1	61|4	9|7
Chr10	48069706	48069706	G	A	SNP	0|0	0|1	67|3	7|6
Chr10	48069747	48069747	T	C	SNP	0|0	0|1	61|3	7|8
Chr10	48069791	48069791	C	A	SNP	0|0	0|1	50|2	6|9
Chr10	48069844	48069844	A	G	SNP	0|1	0|1	55|5	3|13
Chr10	48069930	48069930	A	T	SNP	0|0	0|1	66|4	3|8
Chr10	48070058	48070058	A	T	SNP	0|0	0|1	59|1	3|16
Chr10	48070080	48070080	T	A	SNP	0|0	0|1	58|1	2|17
Chr10	48070342	48070342	T	A	SNP	0|0	0|1	55|2	4|11
Chr10	48070385	48070385	G	T	SNP	0|0	0|1	59|1	6|11
Chr10	48070440	48070440	G	A	SNP	0|0	0|1	55|0	6|16
Chr10	48070997	48070997	G	C	SNP	0|0	0|1	50|0	3|10
Chr10	48071913	48071913	G	A	SNP	0|0	0|1	72|2	2|11
Chr10	48076657	48076657	G	T	SNP	0|0	0|1	49|1	9|15
Chr10	48076977	48076977	T	C	SNP	0|0	0|1	45|1	10|15
Chr10	48079908	48079908	T	A	SNP	0|0	0|1	25|0	3|9
Chr10	48080112	48080112	A	T	SNP	0|0	0|1	25|1	3|12
Chr10	48080280	48080280	C	T	SNP	0|0	1|1	34|4	1|10
Chr10	48081338	48081338	G	C	SNP	0|0	1|1	25|0	0|9

Chr10	48081792	48081792	C	T	SNP	0|0	0|1	10|0	5|3
Chr10	48081843	48081843	G	A	SNP	0|0	0|1	12|0	6|4
Chr10	48081873	48081873	G	A	SNP	0|0	0|1	14|0	6|6
Chr10	48086665	48086665	A	G	SNP	0|0	0|1	44|2	9|10
Chr10	48086773	48086773	A	C	SNP	0|0	0|1	43|1	11|7
Chr10	48087489	48087489	A	G	SNP	0|0	0|1	43|0	5|8
Chr10	48089078	48089078	G	A	SNP	0|0	0|1	71|3	3|6
Chr10	48089109	48089109	A	G	SNP	0|0	0|1	78|3	3|6
Chr10	48089120	48089120	A	G	SNP	0|1	0|1	72|9	4|6
Chr10	48089135	48089135	A	C	SNP	0|0	0|1	76|5	4|6
Chr10	48094127	48094127	G	A	SNP	0|1	0|1	69|11	9|5
Chr10	48097766	48097766	G	T	SNP	0|1	0|1	75|9	4|4
Chr10	48098495	48098495	T	C	SNP	0|1	0|1	55|16	4|9
Chr10	48098506	48098506	T	G	SNP	0|1	0|1	54|19	4|9
Chr10	48098510	48098510	C	T	SNP	0|1	0|1	52|20	4|9
Chr10	48098623	48098623	T	C	SNP	0|1	0|1	41|14	5|13
Chr10	48101115	48101115	A	G	SNP	0|0	0|1	28|1	4|6
Chr10	48101182	48101182	G	A	SNP	0|0	0|1	39|2	6|8
Chr10	48101363	48101363	T	G	SNP	0|1	0|1	41|4	3|10
Chr10	48101434	48101434	A	G	SNP	0|0	0|1	52|1	1|13
Chr10	48102016	48102016	C	A	SNP	0|0	0|1	50|2	7|14
Chr10	48102283	48102283	C	A	SNP	0|0	0|1	41|0	6|16
Chr10	48102950	48102950	C	G	SNP	0|0	0|1	42|1	3|7
Chr10	48103084	48103084	C	A	SNP	0|0	0|1	49|0	4|11
Chr10	48103103	48103103	C	A	SNP	0|0	0|1	44|2	3|11
Chr10	48103467	48103467	T	A	SNP	0|0	0|1	49|2	2|23
Chr10	48103496	48103496	T	G	SNP	0|0	0|1	41|2	4|25
Chr10	48104090	48104090	A	C	SNP	0|0	0|1	47|0	2|13
Chr10	48104142	48104142	A	C	SNP	0|0	0|1	72|0	3|18
Chr10	48104238	48104238	A	T	SNP	0|0	0|1	69|1	5|15
Chr10	48104248	48104248	C	G	SNP	0|0	0|1	71|1	6|15
Chr10	48108761	48108761	A	T	SNP	0|0	0|1	18|1	4|7
Chr10	48108787	48108787	T	C	SNP	0|0	0|1	27|0	5|7
Chr10	48109027	48109027	C	T	SNP	0|0	0|1	48|2	7|18
Chr10	48109032	48109032	T	C	SNP	0|0	0|1	49|2	7|19
Chr10	48109033	48109033	C	A	SNP	0|0	0|1	49|2	7|19
Chr10	48109038	48109038	A	T	SNP	0|0	0|1	49|2	7|19
Chr10	48109082	48109082	C	G	SNP	0|0	0|1	51|2	7|20
Chr10	48109281	48109281	T	G	SNP	0|0	0|1	51|1	6|15
Chr10	48109320	48109320	G	C	SNP	0|0	0|1	48|1	6|13
Chr10	48109341	48109341	T	G	SNP	0|0	0|1	49|1	8|14
Chr10	48109465	48109465	A	C	SNP	0|0	0|1	48|5	12|20

Chr10	48109580	48109580	T	G	SNP	0|0	0|1	54|0	5|19
Chr10	48109602	48109602	C	G	SNP	0|0	0|1	53|0	4|14
Chr10	48109606	48109606	A	T	SNP	0|0	0|1	53|0	5|15
Chr10	48109634	48109634	T	G	SNP	0|0	0|1	56|0	6|11
Chr10	48109637	48109637	C	T	SNP	0|0	0|1	54|0	6|11
Chr10	48109640	48109640	T	A	SNP	0|0	0|1	53|0	7|10
Chr10	48109699	48109699	A	G	SNP	0|0	0|1	65|0	9|7
Chr10	48109884	48109884	T	A	SNP	0|0	0|1	27|0	3|6
Chr10	48109891	48109891	T	C	SNP	0|0	0|1	24|0	3|6
Chr10	48110179	48110179	A	G	SNP	0|0	0|1	49|2	6|18
Chr10	48110186	48110186	C	T	SNP	0|0	0|1	50|2	6|18
Chr10	48110229	48110229	C	T	SNP	0|0	0|1	42|3	5|17
Chr10	48110288	48110288	T	C	SNP	0|0	0|1	50|3	3|12
Chr10	48110333	48110333	A	T	SNP	0|0	0|1	44|3	2|8
Chr10	48110335	48110335	A	C	SNP	0|0	0|1	44|3	2|9
Chr10	48110340	48110340	T	G	SNP	0|0	0|1	48|3	3|9
Chr10	48110346	48110346	G	A	SNP	0|0	0|1	47|2	3|10
Chr10	48110370	48110370	C	A	SNP	0|0	0|1	51|1	4|8
Chr10	48110375	48110375	C	T	SNP	0|0	0|1	50|1	4|7
Chr10	48110453	48110453	C	T	SNP	0|0	0|1	61|1	6|12
Chr10	48110465	48110465	A	G	SNP	0|0	0|1	60|1	7|16
Chr10	48110508	48110508	A	C	SNP	0|0	0|1	73|0	8|21
Chr10	48110687	48110687	C	A	SNP	0|0	0|1	73|2	10|13
Chr10	48113597	48113597	A	G	SNP	0|0	0|1	57|0	5|16
Chr10	48115513	48115513	G	A	SNP	0|0	0|1	49|1	4|12
Chr10	48115559	48115559	A	C	SNP	0|0	0|1	56|1	3|12
Chr10	48115590	48115590	G	A	SNP	0|0	0|1	66|2	2|16
Chr10	48115707	48115707	A	C	SNP	0|0	0|1	47|2	6|17
Chr10	48119600	48119600	T	C	SNP	0|0	0|1	66|1	2|20
Chr10	48119856	48119856	G	T	SNP	0|0	0|1	61|2	5|8
Chr10	48123973	48123973	T	C	SNP	0|0	0|1	25|1	5|5
Chr10	48126201	48126201	C	G	SNP	0|1	0|1	23|2	1|8
Chr10	48126220	48126220	T	C	SNP	0|0	0|1	29|2	3|10
Chr10	48126385	48126385	A	C	SNP	0|0	0|1	62|3	4|9
Chr10	48126525	48126525	G	C	SNP	0|0	0|1	51|3	5|19
Chr10	48126822	48126822	G	A	SNP	0|0	0|1	86|4	6|16
Chr10	48127516	48127516	T	A	SNP	0|0	0|1	67|5	2|6
Chr10	48127550	48127550	G	A	SNP	0|0	0|1	63|3	6|8
Chr10	48127567	48127567	T	G	SNP	0|0	0|1	63|3	5|8
Chr10	48127627	48127627	G	C	SNP	0|0	0|1	50|2	7|8
Chr10	48128304	48128304	G	T	SNP	0|0	0|1	43|0	5|12
Chr10	48128405	48128405	C	T	SNP	0|0	0|1	38|0	3|13

Chr10	48129479	48129479	C	T	SNP	0|0	0|1	69|3	5|15
Chr10	48131992	48131992	T	C	SNP	0|0	0|1	38|1	6|7
Chr10	48132151	48132151	C	T	SNP	0|0	0|1	38|0	2|6
Chr10	48133164	48133164	T	C	SNP	0|0	0|1	71|1	8|13
Chr10	48133919	48133919	A	G	SNP	0|0	0|1	60|3	6|3
Chr10	48134021	48134021	C	T	SNP	0|0	0|1	77|0	8|7
Chr10	48134178	48134178	C	T	SNP	0|0	0|1	89|1	25|11
Chr10	48134228	48134228	A	T	SNP	0|1	0|1	64|28	18|14
Chr10	48134808	48134808	G	A	SNP	0|1	0|0	62|35	11|1
Chr10	48135020	48135020	C	T	SNP	0|0	0|1	62|2	4|6
Chr10	48135252	48135252	G	A	SNP	0|0	0|1	44|1	5|8
Chr10	48135294	48135294	G	A	SNP	0|0	0|1	39|1	4|6
Chr10	48136336	48136336	G	A	SNP	0|0	0|1	46|1	16|12
Chr10	48136364	48136364	T	C	SNP	0|0	0|1	50|0	11|6
Chr10	48136923	48136923	C	T	SNP	0|0	0|1	27|0	8|6
Chr10	48137323	48137323	G	A	SNP	0|1	1|1	33|4	2|22
Chr10	48137473	48137473	A	G	SNP	0|0	0|1	20|0	5|23
Chr10	48138180	48138180	A	G	SNP	0|0	0|1	47|1	3|16
Chr10	48138444	48138444	T	G	SNP	0|1	0|1	55|5	9|23
Chr10	48138570	48138570	T	C	SNP	0|1	0|1	40|3	8|28
Chr10	48138709	48138709	G	A	SNP	0|1	0|1	35|6	5|37
Chr10	48138795	48138795	G	A	SNP	0|1	0|1	41|3	5|27
Chr10	48138803	48138803	A	G	SNP	0|0	0|1	39|2	4|27
Chr10	48146683	48146683	G	A	SNP	0|0	0|1	39|0	4|10
Chr10	48147580	48147580	C	A	SNP	0|0	0|1	26|0	7|7
Chr10	48147731	48147731	C	A	SNP	0|0	0|1	65|5	10|9
Chr10	48147920	48147920	G	C	SNP	0|0	0|1	77|4	7|15
Chr10	48148431	48148431	A	G	SNP	0|0	0|1	65|2	5|24
Chr10	48148722	48148722	T	G	SNP	0|0	0|1	37|1	5|14
Chr10	48148996	48148996	A	G	SNP	0|0	0|1	34|0	6|3
Chr10	48149220	48149220	T	C	SNP	0|0	0|1	29|0	4|5
Chr10	48149975	48149975	G	C	SNP	0|0	0|1	69|2	6|16
Chr10	48152623	48152623	G	C	SNP	0|0	0|1	61|3	8|18
Chr10	48152641	48152641	T	A	SNP	0|0	0|1	56|4	10|15
Chr10	48153052	48153052	C	G	SNP	0|0	0|1	43|1	2|7
Chr10	48153069	48153069	G	A	SNP	0|0	0|1	44|2	2|9
Chr10	48153993	48153993	C	G	SNP	0|0	0|1	21|0	1|7
Chr10	48155030	48155030	T	G	SNP	0|0	0|1	55|0	9|10
Chr10	48155338	48155338	A	G	SNP	0|1	0|1	47|5	9|13
Chr10	48155413	48155413	G	A	SNP	0|1	0|1	40|3	5|15
Chr10	48156314	48156314	T	A	SNP	0|0	0|1	36|1	6|10
Chr10	48156517	48156517	G	T	SNP	0|0	0|1	37|0	4|16

Chr10	48156707	48156707	T	C	SNP	0|1	0|1	36|3	4|6
Chr10	48156850	48156850	G	C	SNP	0|1	0|1	26|2	3|13
Chr10	48156894	48156894	G	A	SNP	0|0	0|1	31|1	1|16
Chr10	48157208	48157208	A	T	SNP	0|0	0|1	48|3	7|15
Chr10	48157302	48157302	A	G	SNP	0|1	0|1	53|4	4|12
Chr10	48157349	48157349	A	T	SNP	0|1	0|1	54|6	5|12
Chr10	48157364	48157364	C	A	SNP	0|1	0|1	56|5	4|13
Chr10	48157418	48157418	C	G	SNP	0|1	0|1	53|5	3|11
Chr10	48157510	48157510	A	G	SNP	0|0	0|1	71|5	3|11
Chr10	48157589	48157589	T	A	SNP	0|1	0|1	60|5	2|7
Chr10	48157632	48157632	T	C	SNP	0|0	0|1	53|3	4|6
Chr10	48157665	48157665	G	A	SNP	0|0	0|1	57|2	5|6
Chr10	48157746	48157746	G	A	SNP	0|0	0|1	56|2	4|4
Chr10	48158372	48158372	A	C	SNP	0|0	0|1	75|5	9|17
Chr10	48158376	48158376	G	C	SNP	0|0	0|1	82|5	9|17
Chr10	48158621	48158621	T	G	SNP	0|0	0|1	69|3	10|25
Chr10	48158673	48158673	C	A	SNP	0|0	0|1	57|3	11|23
Chr10	48158691	48158691	C	T	SNP	0|0	0|1	66|3	10|21
Chr10	48158716	48158716	T	A	SNP	0|0	0|1	61|4	9|18
Chr10	48158794	48158794	G	T	SNP	0|0	0|1	47|2	5|7
Chr10	48158804	48158804	C	G	SNP	0|0	0|1	43|2	4|6
Chr10	48158858	48158858	A	G	SNP	0|1	0|1	35|3	6|10
Chr10	48158866	48158866	C	T	SNP	0|1	0|1	31|3	5|10
Chr10	48159438	48159438	G	A	SNP	0|0	0|1	48|2	4|10
Chr10	48159592	48159592	G	A	SNP	0|0	0|1	43|0	7|20
Chr10	48159616	48159616	G	A	SNP	0|0	0|1	45|0	7|15
Chr10	48159632	48159632	A	G	SNP	0|0	0|1	49|0	9|13
Chr10	48159893	48159893	T	G	SNP	0|0	0|1	48|2	8|9
Chr10	48159970	48159970	C	A	SNP	0|0	0|1	61|2	4|7
Chr10	48159989	48159989	G	T	SNP	0|0	0|1	56|2	6|7
Chr10	48160265	48160265	T	A	SNP	0|1	0|1	44|7	5|9
Chr10	48160408	48160408	G	A	SNP	0|0	0|1	56|2	6|20
Chr10	48160684	48160684	A	G	SNP	0|0	0|1	60|2	7|19
Chr10	48160911	48160911	A	T	SNP	0|0	0|1	52|1	2|7
Chr10	48160930	48160930	A	G	SNP	0|0	0|1	57|1	2|9
Chr10	48161072	48161072	C	T	SNP	0|0	0|1	51|3	5|12
Chr10	48161195	48161195	T	C	SNP	0|0	0|1	39|1	3|7
Chr10	48161674	48161674	A	C	SNP	0|0	0|1	37|1	6|11
Chr10	48161733	48161733	G	A	SNP	0|0	0|1	37|2	6|9
Chr10	48161951	48161951	C	T	SNP	0|0	0|1	24|0	2|7
Chr10	48161985	48161985	A	T	SNP	0|0	0|1	27|0	2|8
Chr10	48161992	48161992	T	A	SNP	0|0	0|1	28|0	1|8

Chr10	48162189	48162189	G	A	SNP	0|0	0|1	53|1	8|14
Chr10	48162833	48162833	A	G	SNP	0|0	0|1	67|1	3|10
Chr10	48163058	48163058	G	A	SNP	0|0	0|1	33|1	1|9
Chr10	48166465	48166465	A	G	SNP	0|0	0|1	51|0	3|12
Chr10	48166502	48166502	T	G	SNP	0|0	0|1	57|1	3|10
Chr10	48166727	48166727	G	A	SNP	0|0	0|1	51|2	3|18
Chr10	48167045	48167045	T	C	SNP	0|0	0|1	49|5	3|17
Chr10	48167228	48167228	C	A	SNP	0|0	0|1	22|0	3|8
Chr10	48169865	48169865	T	C	SNP	0|0	0|1	14|0	5|5
Chr10	48169866	48169866	T	C	SNP	0|0	0|1	14|0	5|5
Chr10	48169867	48169867	T	A	SNP	0|0	0|1	14|0	5|5
Chr10	48169926	48169926	T	C	SNP	0|0	0|1	21|0	3|7
Chr10	48170570	48170570	G	A	SNP	0|0	0|1	80|1	6|32
Chr10	48170798	48170798	G	C	SNP	0|0	0|1	89|2	11|18
Chr10	48170966	48170966	G	A	SNP	0|0	0|1	45|0	1|8
Chr10	48174348	48174348	G	A	SNP	0|0	0|1	59|0	9|7
Chr10	48174986	48174986	G	A	SNP	0|0	1|1	34|0	0|11
Chr10	48175102	48175102	G	A	SNP	0|0	0|1	32|1	2|6
Chr10	48176022	48176022	G	C	SNP	0|0	0|1	17|0	2|9
Chr10	48176609	48176609	A	G	SNP	0|0	0|1	19|0	5|8
Chr10	48176655	48176655	A	C	SNP	0|0	0|1	25|0	3|6
Chr10	48176829	48176829	T	C	SNP	0|0	0|1	24|2	4|9
Chr10	48176835	48176835	C	A	SNP	0|0	0|1	28|2	4|9
Chr10	48176862	48176862	T	C	SNP	0|0	0|1	29|2	5|7
Chr10	48176870	48176870	G	A	SNP	0|0	0|1	28|2	5|7
Chr10	48176880	48176880	T	C	SNP	0|1	0|1	32|2	6|6
Chr10	48176896	48176896	T	C	SNP	0|1	0|1	32|4	2|6
Chr10	48176901	48176901	T	C	SNP	0|1	0|1	32|4	3|6
Chr10	48176905	48176905	T	A	SNP	0|1	0|1	31|4	3|6
Chr10	48176969	48176969	T	C	SNP	0|1	0|1	17|2	4|5
Chr10	48177305	48177305	T	C	SNP	0|0	0|1	22|0	3|6
Chr10	48177338	48177338	C	T	SNP	0|0	0|1	16|0	3|6
Chr10	48177421	48177421	A	G	SNP	0|0	0|1	20|0	6|5
Chr10	48177440	48177440	T	C	SNP	0|0	0|1	19|0	6|5
Chr10	48179081	48179081	T	G	SNP	0|0	0|1	17|0	7|7
Chr10	48179090	48179090	A	T	SNP	0|0	0|1	18|0	7|7
Chr10	48179139	48179139	T	G	SNP	0|0	0|1	11|0	5|8
Chr10	48180457	48180457	A	G	SNP	0|0	0|1	42|1	7|16
Chr10	48180559	48180559	A	G	SNP	0|0	0|1	36|1	8|10
Chr10	48180581	48180581	T	G	SNP	0|0	0|1	37|1	9|12
Chr10	48180586	48180586	A	G	SNP	0|0	0|1	39|1	9|12
Chr10	48180591	48180591	A	T	SNP	0|0	0|1	41|1	9|11

Chr10	48180671	48180671	A	C	SNP	0|0	0|1	61|1	9|10
Chr10	48180770	48180770	A	C	SNP	0|0	0|1	51|0	8|14
Chr10	48180812	48180812	C	A	SNP	0|0	0|1	56|1	7|17
Chr10	48180844	48180844	C	T	SNP	0|0	0|1	60|2	4|13
Chr10	48180853	48180853	G	A	SNP	0|0	0|1	57|2	4|10
Chr10	48180861	48180861	A	G	SNP	0|0	0|1	59|2	5|11
Chr10	48180877	48180877	T	G	SNP	0|0	0|1	60|3	5|13
Chr10	48181171	48181171	G	T	SNP	0|1	0|1	65|5	3|14
Chr10	48181317	48181317	T	C	SNP	0|0	0|1	72|4	1|14
Chr10	48185607	48185607	C	G	SNP	0|0	0|1	21|0	1|19
Chr10	48186896	48186896	A	T	SNP	0|0	0|1	34|2	4|11
Chr10	48187146	48187146	T	C	SNP	0|0	0|1	19|0	4|4
Chr10	48187341	48187341	A	G	SNP	0|0	0|1	56|3	3|16
Chr10	48187463	48187463	T	C	SNP	0|0	0|1	49|0	4|4
Chr10	48187564	48187564	A	C	SNP	0|0	0|1	42|0	3|8
Chr10	48187648	48187648	G	T	SNP	0|0	0|1	48|1	5|8
Chr10	48187654	48187654	A	C	SNP	0|0	0|1	46|1	5|9
Chr10	48189259	48189259	A	C	SNP	0|0	0|1	63|1	11|7
Chr10	48189270	48189270	G	C	SNP	0|0	0|1	59|1	11|7
Chr10	48189444	48189444	C	G	SNP	0|0	0|1	39|0	8|14
Chr10	48189525	48189525	C	T	SNP	0|0	0|1	43|0	7|14
Chr10	48189638	48189638	C	A	SNP	0|0	0|1	31|1	9|8
Chr10	48189648	48189648	C	A	SNP	0|0	0|1	28|1	9|8
Chr10	48189759	48189759	A	T	SNP	0|0	0|1	22|1	6|8
Chr10	48189840	48189840	A	T	SNP	0|0	0|1	30|0	3|12
Chr10	48189841	48189841	A	C	SNP	0|0	0|1	29|0	3|12
Chr10	48189843	48189843	C	T	SNP	0|0	0|1	29|0	3|12
Chr10	48189896	48189896	G	T	SNP	0|0	1|1	39|1	0|13
Chr10	48190027	48190027	T	C	SNP	0|0	0|1	31|2	10|6
Chr10	48190247	48190247	A	G	SNP	0|0	0|1	29|2	5|13
Chr10	48190264	48190264	T	G	SNP	0|0	0|1	35|1	4|13
Chr10	48190300	48190300	G	A	SNP	0|0	0|1	36|1	5|8
Chr10	48190406	48190406	A	G	SNP	0|0	0|1	34|2	2|10
Chr10	48190594	48190594	T	C	SNP	0|0	0|1	48|3	1|14
Chr10	48190626	48190626	G	T	SNP	0|0	0|1	45|3	2|14
Chr10	48190689	48190689	G	A	SNP	0|1	0|1	35|3	7|13
Chr10	48192432	48192432	C	G	SNP	0|0	0|1	65|4	3|13
Chr10	48196522	48196522	C	T	SNP	0|0	0|1	35|1	1|9
Chr10	48196769	48196769	T	A	SNP	0|0	0|1	50|3	1|12
Chr10	48197057	48197057	A	G	SNP	0|0	0|1	61|2	4|22
Chr10	48197290	48197290	G	T	SNP	0|0	0|1	58|6	9|13
Chr10	48200490	48200490	A	T	SNP	0|0	0|1	15|0	1|7

Chr10	48200516	48200516	T	C	SNP	0|0	0|1	14|0	2|6
Chr10	48200898	48200898	A	G	SNP	0|0	0|1	29|1	1|8
Chr10	48201278	48201278	G	C	SNP	0|0	0|1	50|0	4|10
Chr10	48201407	48201407	A	C	SNP	0|0	0|1	51|1	3|20
Chr10	48201741	48201741	C	G	SNP	0|0	0|1	44|0	3|15
Chr10	48202732	48202732	G	C	SNP	0|0	0|1	57|1	5|16
Chr10	48202738	48202738	C	A	SNP	0|0	0|1	58|1	5|16
Chr10	48202955	48202955	G	A	SNP	0|0	0|1	47|0	3|12
Chr10	48202994	48202994	A	G	SNP	0|0	0|1	44|0	8|7
Chr10	48203039	48203039	T	A	SNP	0|0	0|1	56|0	9|7
Chr10	48203628	48203628	C	T	SNP	0|0	0|1	43|0	2|14
Chr10	48203659	48203659	T	C	SNP	0|0	0|1	43|1	2|14
Chr10	48203684	48203684	C	T	SNP	0|0	0|1	40|1	5|12
Chr10	48204187	48204187	G	A	SNP	0|0	0|1	54|4	1|9
Chr10	48204229	48204229	G	A	SNP	0|0	0|1	56|3	1|14
Chr10	48204250	48204250	A	T	SNP	0|0	0|1	56|2	1|14
Chr10	48204252	48204252	A	G	SNP	0|0	0|1	54|1	1|13
Chr10	48204295	48204295	G	A	SNP	0|0	0|1	49|2	3|11
Chr10	48204658	48204658	T	A	SNP	0|1	0|1	36|5	8|10
Chr10	48204717	48204717	A	C	SNP	0|0	0|1	41|3	7|5
Chr10	48204786	48204786	C	G	SNP	0|0	0|1	54|3	6|4
Chr10	48204799	48204799	T	C	SNP	0|0	0|1	55|2	6|3
Chr10	48204933	48204933	G	A	SNP	0|0	0|1	25|0	6|4
Chr10	48205224	48205224	C	G	SNP	0|0	0|1	20|0	4|6
Chr10	48205441	48205441	G	A	SNP	0|0	0|1	28|1	4|12
Chr10	48206041	48206041	C	T	SNP	0|0	0|1	38|1	6|5
Chr10	48206200	48206200	C	T	SNP	0|0	0|1	46|0	1|9
Chr10	48206202	48206202	G	C	SNP	0|0	0|1	45|0	1|9
Chr10	48206659	48206659	A	G	SNP	0|0	0|1	22|0	5|10
Chr10	48206660	48206660	C	T	SNP	0|0	0|1	24|0	5|10
Chr10	48206707	48206707	G	C	SNP	0|0	0|1	16|0	5|8
Chr10	48206735	48206735	G	C	SNP	0|0	0|1	14|0	6|6
Chr10	48206747	48206747	G	T	SNP	0|0	0|1	14|0	6|4
Chr10	48206748	48206748	T	G	SNP	0|0	0|1	13|0	6|4
Chr10	48208974	48208974	C	T	SNP	0|0	0|1	37|0	7|7
Chr10	48209094	48209094	G	A	SNP	0|0	0|1	32|1	6|6
Chr10	48211656	48211656	A	G	SNP	0|0	1|1	27|1	0|8
Chr10	48212299	48212299	T	C	SNP	0|0	0|1	41|1	4|15
Chr10	48213028	48213028	C	G	SNP	0|0	0|1	52|2	7|14
Chr10	48213046	48213046	T	C	SNP	0|0	0|1	49|2	8|14
Chr10	48213272	48213272	T	G	SNP	0|0	0|1	44|2	7|16
Chr10	48213286	48213286	T	C	SNP	0|0	0|1	46|2	7|16

Chr10	48213290	48213290	A	G	SNP	0|0	0|1	46|2	7|16
Chr10	48213295	48213295	C	T	SNP	0|0	0|1	45|2	8|17
Chr10	48213304	48213304	A	G	SNP	0|0	0|1	45|2	7|17
Chr10	48213335	48213335	A	G	SNP	0|0	0|1	43|3	11|16
Chr10	48213344	48213344	A	G	SNP	0|0	0|1	47|3	11|17
Chr10	48213377	48213377	T	C	SNP	0|1	0|1	51|5	8|13
Chr10	48213395	48213395	T	A	SNP	0|1	0|1	51|4	12|8
Chr10	48213619	48213619	C	T	SNP	0|0	0|1	61|4	5|21
Chr10	48213624	48213624	A	G	SNP	0|0	0|1	62|4	5|22
Chr10	48213898	48213898	A	G	SNP	0|0	0|1	49|2	6|12
Chr10	48213938	48213938	C	T	SNP	0|0	0|1	44|1	5|9
Chr10	48214054	48214054	T	A	SNP	0|0	0|1	45|1	8|11
Chr10	48214586	48214586	T	C	SNP	0|0	0|1	32|2	5|17
Chr10	48214607	48214607	A	G	SNP	0|0	0|1	30|1	5|13
Chr10	48214611	48214611	C	T	SNP	0|0	0|1	30|1	5|10
Chr10	48215185	48215185	A	T	SNP	0|0	0|1	62|1	6|11
Chr10	48215707	48215707	G	A	SNP	0|0	0|1	79|1	5|17
Chr10	48216342	48216342	A	G	SNP	0|0	0|1	48|0	3|15
Chr10	48216362	48216362	G	A	SNP	0|0	0|1	41|0	5|18
Chr10	48216572	48216572	C	A	SNP	0|0	0|1	39|0	3|15
Chr10	48216629	48216629	T	A	SNP	0|0	0|1	36|0	5|14
Chr10	48216654	48216654	G	C	SNP	0|0	0|1	34|0	5|14
Chr10	48217620	48217620	C	T	SNP	0|0	0|1	30|1	3|14
Chr10	48218131	48218131	A	G	SNP	0|1	0|1	45|8	3|10
Chr10	48218303	48218303	G	C	SNP	0|1	0|1	54|5	2|9
Chr10	48218414	48218414	T	C	SNP	0|0	0|1	66|4	5|24
Chr10	48218474	48218474	G	A	SNP	0|0	0|1	55|2	5|21
Chr10	48218759	48218759	C	T	SNP	0|0	0|1	60|0	9|13
Chr10	48218821	48218821	C	T	SNP	0|0	0|1	47|0	9|12
Chr10	48218847	48218847	T	C	SNP	0|0	0|1	49|0	8|13
Chr10	48218894	48218894	A	G	SNP	0|0	0|1	43|1	7|17
Chr10	48218897	48218897	G	A	SNP	0|0	0|1	41|1	7|18
Chr10	48219104	48219104	G	T	SNP	0|0	0|1	50|0	9|6
Chr10	48219309	48219309	A	T	SNP	0|0	0|1	61|4	4|6
Chr10	48219684	48219684	T	C	SNP	0|0	0|1	54|2	7|8
Chr10	48219849	48219849	G	A	SNP	0|0	0|1	35|0	7|6
Chr10	48220061	48220061	A	G	SNP	0|1	0|1	44|5	4|7
Chr10	48220117	48220117	C	A	SNP	0|0	0|1	48|4	6|7
Chr10	48220797	48220797	A	C	SNP	0|0	0|1	31|0	3|14
Chr10	48220870	48220870	T	A	SNP	0|0	0|1	26|0	3|13
Chr10	48222094	48222094	C	A	SNP	0|0	0|1	47|0	5|23
Chr10	48222745	48222745	C	T	SNP	0|1	0|1	25|4	3|9

Chr10	48222952	48222952	T	A	SNP	0|1	0|1	26|2	4|8
Chr10	48223222	48223222	C	G	SNP	0|0	0|1	50|1	5|8
Chr10	48223298	48223298	T	A	SNP	0|0	0|1	43|3	1|9
Chr10	48223320	48223320	T	A	SNP	0|1	0|1	39|2	1|8
Chr10	48224261	48224261	G	A	SNP	0|0	0|1	18|0	1|9
Chr10	48224265	48224265	C	T	SNP	0|0	0|1	16|0	1|9
Chr10	48224552	48224552	A	G	SNP	0|0	0|1	33|1	2|10
Chr10	48224770	48224770	G	A	SNP	0|0	0|1	22|0	2|9
Chr10	48224776	48224776	T	G	SNP	0|0	0|1	21|1	1|10
Chr10	48224797	48224797	T	C	SNP	0|0	0|1	19|1	1|7
Chr10	48236216	48236216	T	A	SNP	0|0	0|1	47|0	3|10
Chr10	48237848	48237848	T	C	SNP	0|0	0|1	42|1	6|5
Chr10	48238429	48238429	T	C	SNP	0|0	0|1	74|4	5|22
Chr10	48239250	48239250	C	T	SNP	0|0	0|1	53|2	7|11
Chr10	48239291	48239291	A	G	SNP	0|0	0|1	46|3	4|13
Chr10	48239628	48239628	T	C	SNP	0|1	0|1	29|7	6|9
Chr10	48239629	48239629	T	A	SNP	0|1	0|1	29|7	6|9
Chr10	48239658	48239658	T	G	SNP	0|1	0|1	30|7	4|9
Chr10	48239838	48239838	T	A	SNP	0|0	0|1	43|4	8|9
Chr10	48239896	48239896	G	A	SNP	0|1	0|1	44|5	4|9
Chr10	48239937	48239937	C	T	SNP	0|0	0|1	52|3	2|9
Chr10	48239947	48239947	A	C	SNP	0|0	0|1	48|2	2|7
Chr10	48240084	48240084	G	C	SNP	0|0	0|1	45|1	4|7
Chr10	48240277	48240277	C	T	SNP	0|0	0|1	44|1	4|5
Chr10	48240320	48240320	C	G	SNP	0|0	0|1	66|1	4|5
Chr10	48240615	48240615	A	T	SNP	0|0	0|1	25|1	5|4
Chr10	48240892	48240892	A	C	SNP	0|0	0|1	17|0	1|10
Chr10	48242467	48242467	T	G	SNP	0|0	0|1	49|3	3|21
Chr10	48242896	48242896	A	G	SNP	0|0	0|1	55|1	5|12
Chr10	48243814	48243814	T	G	SNP	0|0	0|1	35|0	3|12
Chr10	48243826	48243826	A	G	SNP	0|0	0|1	39|0	3|11
Chr10	48246096	48246096	C	T	SNP	0|0	0|1	18|1	2|12
Chr10	48246792	48246792	A	T	SNP	0|0	0|1	47|3	3|9
Chr10	48246825	48246825	G	A	SNP	0|1	0|1	41|4	3|9
Chr10	48246948	48246948	A	G	SNP	0|1	0|1	38|4	2|9
Chr10	48247077	48247077	A	G	SNP	0|1	1|1	34|3	0|16
Chr10	48247094	48247094	T	A	SNP	0|1	1|1	36|3	0|15
Chr10	48247120	48247120	A	G	SNP	0|1	1|1	29|3	0|14
Chr10	48247150	48247150	T	C	SNP	0|1	1|1	25|3	0|10
Chr10	48247173	48247173	T	C	SNP	0|1	0|1	28|3	1|9
Chr10	48252164	48252164	T	C	SNP	0|0	0|1	58|2	7|14
Chr10	48252795	48252795	C	T	SNP	0|0	0|1	40|0	11|19

Chr10	48253038	48253038	T	G	SNP	0|0	0|1	30|2	7|12
Chr10	48253103	48253103	G	T	SNP	0|0	0|1	34|1	3|14
Chr10	48253198	48253198	C	T	SNP	0|0	0|1	48|1	3|6
Chr10	48255955	48255955	T	C	SNP	0|0	0|1	28|0	4|10
Chr10	48256879	48256879	T	C	SNP	0|0	0|1	57|3	9|10
Chr10	48257606	48257606	G	T	SNP	0|0	0|1	52|3	5|17
Chr10	48260027	48260027	A	C	SNP	0|0	0|1	64|3	6|21
Chr10	48263691	48263691	C	T	SNP	0|1	0|1	12|1	3|9
Chr10	48263737	48263737	G	A	SNP	0|1	0|1	8|1	2|8
Chr10	48264381	48264381	T	A	SNP	0|0	0|1	56|0	5|16
Chr10	48264636	48264636	A	G	SNP	0|0	0|1	56|1	3|20
Chr10	48264930	48264930	G	A	SNP	0|0	0|1	41|0	6|18
Chr10	48266020	48266020	G	A	SNP	0|0	0|1	70|1	5|10
Chr10	48266382	48266382	A	T	SNP	0|0	0|1	70|3	11|18
Chr10	48269828	48269828	A	T	SNP	0|0	0|1	38|1	8|20
Chr10	48275463	48275463	A	C	SNP	0|1	0|1	24|3	6|12
Chr10	48276647	48276647	C	A	SNP	0|0	0|1	37|1	7|11
Chr10	48277081	48277081	A	G	SNP	0|1	0|1	26|2	5|6
Chr10	48277441	48277441	C	A	SNP	0|0	0|1	25|0	1|7
Chr10	48277538	48277538	A	G	SNP	0|0	0|1	42|0	8|8
Chr10	48277643	48277643	G	A	SNP	0|0	0|1	34|0	9|14
Chr10	48279063	48279063	T	G	SNP	0|0	0|1	33|1	2|8
Chr10	48279379	48279379	C	T	SNP	0|1	0|1	46|4	5|10
Chr10	48279394	48279394	A	T	SNP	0|0	0|1	43|2	4|10
Chr10	48281020	48281020	A	C	SNP	0|0	0|1	47|0	7|13
Chr10	48281279	48281279	T	G	SNP	0|0	0|1	54|1	6|15
Chr10	48281521	48281521	A	T	SNP	0|0	0|1	50|2	4|16
Chr10	48284965	48284965	C	T	SNP	0|0	0|1	56|1	7|18
Chr10	48285710	48285710	A	G	SNP	0|0	0|1	57|1	4|10
Chr10	48285828	48285828	G	A	SNP	0|0	0|1	49|1	7|9
Chr10	48285951	48285951	T	C	SNP	0|0	0|1	65|2	5|12
Chr10	48288496	48288496	G	A	SNP	0|0	0|1	36|0	4|8
Chr10	48289055	48289055	A	G	SNP	0|0	0|1	79|1	2|11
Chr10	48292473	48292473	T	A	SNP	0|0	0|1	24|1	2|8
Chr10	48294007	48294007	C	G	SNP	0|1	0|1	53|7	3|9
Chr10	48296350	48296350	A	T	SNP	0|1	0|1	23|2	4|4
Chr10	48296714	48296714	A	T	SNP	0|0	0|1	28|0	2|10
Chr10	48299992	48299992	A	G	SNP	0|0	0|1	40|2	5|18
Chr10	48300216	48300216	T	C	SNP	0|0	0|1	40|1	5|12
Chr10	48304438	48304438	G	C	SNP	0|1	0|1	16|3	1|7
Chr10	48304612	48304612	A	G	SNP	0|0	0|1	42|0	4|9
Chr10	48304629	48304629	T	A	SNP	0|0	0|1	39|0	4|9

Chr10	48304831	48304831	G	A	SNP	0|0	0|1	30|0	2|11
Chr10	48305914	48305914	A	T	SNP	0|0	0|1	40|1	2|11
Chr10	48306059	48306059	T	C	SNP	0|0	0|1	39|1	2|13
Chr10	48306195	48306195	A	T	SNP	0|1	0|1	40|3	10|10
Chr10	48306205	48306205	C	A	SNP	0|0	0|1	44|3	10|8
Chr10	48306326	48306326	G	T	SNP	0|0	0|1	50|4	6|12
Chr10	48307575	48307575	A	C	SNP	0|0	0|1	43|2	8|10
Chr10	48307983	48307983	G	A	SNP	0|0	0|1	31|1	2|8
Chr10	48308210	48308210	G	T	SNP	0|0	0|1	63|2	4|14
Chr10	48308537	48308537	G	A	SNP	0|0	0|1	37|0	5|8
Chr10	48308538	48308538	T	A	SNP	0|0	0|1	37|0	5|8
Chr10	48309399	48309399	C	G	SNP	0|0	0|1	26|1	3|12
Chr10	48310482	48310482	A	G	SNP	0|0	0|1	29|0	6|16
Chr10	48310796	48310796	C	A	SNP	0|0	0|1	50|1	8|11
Chr10	48310915	48310915	A	C	SNP	0|0	0|1	45|1	2|18
Chr10	48311131	48311131	C	T	SNP	0|0	1|1	46|4	2|16
Chr10	48311243	48311243	G	A	SNP	0|0	0|1	53|3	4|13
Chr10	48311413	48311413	A	G	SNP	0|0	0|1	51|2	2|10
Chr10	48311751	48311751	C	T	SNP	0|0	0|1	57|3	4|11
Chr10	48311840	48311840	C	T	SNP	0|0	0|1	65|3	12|12
Chr10	48312410	48312410	T	C	SNP	0|1	0|1	43|5	5|24
Chr10	48312505	48312505	C	T	SNP	0|0	0|1	52|3	6|15
Chr10	48312521	48312521	T	G	SNP	0|0	0|1	51|3	5|15
Chr10	48312523	48312523	T	G	SNP	0|0	0|1	51|3	5|15
Chr10	48312534	48312534	C	G	SNP	0|1	0|1	47|4	5|15
Chr10	48312538	48312538	C	T	SNP	0|1	0|1	44|4	5|16
Chr10	48312605	48312605	C	G	SNP	0|1	0|1	37|3	4|9
Chr10	48312646	48312646	C	A	SNP	0|1	0|1	32|4	4|10
Chr10	48312737	48312737	G	C	SNP	0|0	0|1	38|1	2|15
Chr10	48312797	48312797	T	C	SNP	0|0	0|1	41|0	6|17
Chr10	48312812	48312812	T	G	SNP	0|0	0|1	39|0	6|17
Chr10	48312824	48312824	A	T	SNP	0|0	0|1	37|0	7|9
Chr10	48312826	48312826	C	A	SNP	0|0	0|1	40|0	7|9
Chr10	48312863	48312863	A	G	SNP	0|1	0|1	38|3	5|10
Chr10	48312895	48312895	A	T	SNP	0|0	0|1	42|3	9|11
Chr10	48312897	48312897	T	G	SNP	0|0	0|1	43|3	9|11
Chr10	48312937	48312937	T	C	SNP	0|1	0|1	42|7	5|12
Chr10	48312999	48312999	C	T	SNP	0|1	0|1	38|5	4|13
Chr10	48313520	48313520	G	A	SNP	0|0	0|1	29|0	5|4
Chr10	48313586	48313586	G	A	SNP	0|0	0|1	26|1	4|4
Chr10	48313695	48313695	A	G	SNP	0|1	0|1	14|2	4|8
Chr10	48313702	48313702	G	C	SNP	0|1	0|1	15|2	5|10

Chr10	48313802	48313802	A	G	SNP	0|0	0|1	29|0	5|10
Chr10	48314005	48314005	G	A	SNP	0|0	0|1	33|3	4|7
Chr10	48314114	48314114	A	T	SNP	0|0	0|1	30|2	2|10
Chr10	48314180	48314180	T	G	SNP	0|0	0|1	37|2	8|11
Chr10	48314249	48314249	C	T	SNP	0|1	0|1	35|3	9|10
Chr10	48314305	48314305	T	C	SNP	0|1	0|1	34|4	9|14
Chr10	48314380	48314380	T	A	SNP	0|1	0|1	37|4	1|11
Chr10	48314386	48314386	A	T	SNP	0|1	0|1	36|4	1|11
Chr10	48314442	48314442	A	C	SNP	0|0	0|1	37|1	1|8
Chr10	48314946	48314946	T	A	SNP	0|1	0|1	38|3	7|7
Chr10	48315006	48315006	T	C	SNP	0|0	0|1	39|2	8|8
Chr10	48315128	48315128	T	C	SNP	0|1	0|1	42|6	5|16
Chr10	48315338	48315338	A	T	SNP	0|0	0|1	28|2	6|9
Chr10	48315347	48315347	A	T	SNP	0|1	0|1	27|2	5|7
Chr10	48315430	48315430	C	A	SNP	0|1	0|1	37|3	6|10
Chr10	48316118	48316118	G	T	SNP	0|0	0|1	16|1	8|10
Chr10	48316878	48316878	A	G	SNP	0|0	0|1	16|0	2|7
Chr10	48318123	48318123	A	G	SNP	0|0	0|1	10|0	2|8
Chr10	48318159	48318159	A	G	SNP	0|0	0|1	9|0	2|8
Chr10	48321570	48321570	C	A	SNP	0|0	0|1	21|1	5|7
Chr10	48322132	48322132	A	G	SNP	0|0	0|1	35|0	5|3
Chr10	48322465	48322465	T	A	SNP	0|0	0|1	46|1	9|9
Chr10	48324168	48324168	A	T	SNP	0|1	0|1	20|2	2|6
Chr10	48324271	48324271	T	G	SNP	0|0	0|1	27|1	1|9
Chr10	48324684	48324684	A	C	SNP	0|0	0|1	24|1	1|8
Chr10	48324748	48324748	A	G	SNP	0|0	0|1	33|1	1|7
Chr10	48325841	48325841	C	G	SNP	0|1	0|1	27|5	5|9
Chr10	48326406	48326406	T	A	SNP	0|1	0|1	34|3	2|6
Chr10	48326528	48326528	G	A	SNP	0|0	0|1	45|5	10|11
Chr10	48326904	48326904	G	A	SNP	0|1	0|1	50|4	3|14
Chr10	48327084	48327084	T	G	SNP	0|1	0|1	19|4	2|12
Chr10	48327349	48327349	A	G	SNP	0|1	0|1	32|4	3|9
Chr10	48327713	48327713	A	G	SNP	0|0	0|1	66|1	7|24
Chr10	48328186	48328186	A	T	SNP	0|0	0|1	53|4	6|14
Chr10	48328296	48328296	C	G	SNP	0|0	0|1	55|0	9|13
Chr10	48328865	48328865	C	T	SNP	0|0	0|1	13|0	3|5
Chr10	48328949	48328949	G	T	SNP	0|0	0|1	18|0	2|7
Chr10	48331567	48331567	C	G	SNP	0|0	0|1	33|1	3|13
Chr10	48331792	48331792	A	G	SNP	0|1	0|1	43|4	1|10
Chr10	48332365	48332365	G	C	SNP	0|1	0|1	33|4	3|8
Chr10	48333876	48333876	C	A	SNP	0|0	0|1	43|0	6|14
Chr10	48335105	48335105	T	C	SNP	0|0	0|1	72|0	6|16

Chr10	48335278	48335278	T	C	SNP	0|0	1|1	79|4	1|21
Chr10	48335590	48335590	T	A	SNP	0|0	0|1	49|1	6|15
Chr10	48335885	48335885	C	T	SNP	0|0	0|1	49|2	1|12
Chr10	48335969	48335969	A	C	SNP	0|0	0|1	58|6	7|11
Chr10	48336236	48336236	G	T	SNP	0|0	0|1	52|2	5|12
Chr10	48336471	48336471	A	G	SNP	0|0	0|1	56|2	3|14
Chr10	48336684	48336684	A	T	SNP	0|0	0|1	45|3	4|10
Chr10	48336808	48336808	A	G	SNP	0|0	0|1	49|2	11|17
Chr10	48337240	48337240	C	T	SNP	0|0	0|1	53|1	2|9
Chr10	48337257	48337257	T	C	SNP	0|0	0|1	51|1	3|14
Chr10	48337269	48337269	T	C	SNP	0|0	0|1	46|1	4|12
Chr10	48337316	48337316	C	T	SNP	0|0	0|1	40|1	3|14
Chr10	48337328	48337328	G	A	SNP	0|0	0|1	41|1	3|12
Chr10	48337374	48337374	C	T	SNP	0|0	0|1	39|0	5|13
Chr10	48337376	48337376	G	C	SNP	0|0	0|1	39|0	5|14
Chr10	48337477	48337477	G	C	SNP	0|1	0|1	37|3	7|8
Chr10	48337489	48337489	T	C	SNP	0|1	0|1	38|3	6|6
Chr10	48337550	48337550	G	A	SNP	0|0	0|1	42|3	7|4
Chr10	48337589	48337589	G	C	SNP	0|0	0|1	45|3	7|5
Chr10	48337768	48337768	A	G	SNP	0|0	0|1	40|0	5|3
Chr10	48337886	48337886	T	C	SNP	0|0	0|1	21|0	9|4
Chr10	48337892	48337892	G	A	SNP	0|0	0|1	20|0	9|4
Chr10	48337926	48337926	A	G	SNP	0|0	0|1	21|0	8|4
Chr10	48337946	48337946	A	G	SNP	0|0	0|1	20|0	8|4
Chr10	48337953	48337953	C	T	SNP	0|0	0|1	21|0	8|4
Chr10	48338067	48338067	G	T	SNP	0|0	0|1	27|0	7|5
Chr10	48338074	48338074	C	T	SNP	0|0	0|1	26|0	7|5
Chr10	48338086	48338086	C	T	SNP	0|0	0|1	31|0	6|5
Chr10	48338093	48338093	T	C	SNP	0|0	0|1	32|0	6|5
Chr10	48338400	48338400	C	T	SNP	0|0	0|1	44|3	5|7
Chr10	48338721	48338721	C	T	SNP	0|0	0|1	36|0	2|6
Chr10	48339194	48339194	G	C	SNP	0|0	0|1	29|0	6|11
Chr10	48341587	48341587	C	T	SNP	0|0	0|1	18|0	4|10
Chr10	48342291	48342291	C	G	SNP	0|0	0|1	45|0	1|8
Chr10	48342675	48342675	T	C	SNP	0|0	0|1	27|0	1|8
Chr10	48342720	48342720	T	A	SNP	0|0	1|1	18|0	0|10
Chr10	48344148	48344148	C	T	SNP	0|0	0|1	51|3	11|24
Chr10	48344287	48344287	G	A	SNP	0|0	0|1	68|4	7|13
Chr10	48344980	48344980	T	G	SNP	0|0	0|1	41|2	4|24
Chr10	48345658	48345658	A	C	SNP	0|0	0|1	53|2	5|11
Chr10	48345795	48345795	C	A	SNP	0|0	0|1	31|1	2|9
Chr10	48346578	48346578	T	A	SNP	0|0	0|1	58|0	5|15

Chr10	48347436	48347436	G	A	SNP	0|0	0|1	59|4	4|14
Chr10	48347543	48347543	A	C	SNP	0|0	0|1	58|1	3|10
Chr10	48347975	48347975	G	A	SNP	0|0	0|1	61|2	7|15
Chr10	48348238	48348238	T	C	SNP	0|0	0|1	67|0	4|20
Chr10	48348273	48348273	G	A	SNP	0|0	0|1	69|0	5|23
Chr10	48348670	48348670	G	A	SNP	0|0	0|1	43|2	1|11
Chr10	48348719	48348719	T	C	SNP	0|0	0|1	32|1	6|8
Chr10	48348860	48348860	G	T	SNP	0|0	0|1	40|2	8|10
Chr10	48349113	48349113	C	T	SNP	0|1	0|1	52|4	12|11
Chr10	48350115	48350115	G	C	SNP	0|0	0|1	74|3	8|14
Chr10	48350643	48350643	C	A	SNP	0|1	0|1	51|4	1|13
Chr10	48350929	48350929	G	T	SNP	0|1	0|1	22|3	11|9
Chr10	48351188	48351188	A	C	SNP	0|1	0|1	39|3	2|14
Chr10	48351337	48351337	A	G	SNP	0|0	0|1	45|2	4|9
Chr10	48351920	48351920	G	A	SNP	0|0	0|1	63|1	10|14
Chr10	48351970	48351970	G	A	SNP	0|0	0|1	53|1	8|17
Chr10	48351974	48351974	C	G	SNP	0|0	0|1	53|1	8|19
Chr10	48352004	48352004	A	G	SNP	0|0	0|1	46|0	5|17
Chr10	48352196	48352196	C	T	SNP	0|0	0|1	13|0	2|7
Chr10	48353297	48353297	G	A	SNP	0|0	0|1	40|2	3|25
Chr10	48353449	48353449	C	T	SNP	0|0	0|1	54|2	6|17
Chr10	48353603	48353603	T	C	SNP	0|0	0|1	33|2	6|9
Chr10	48353653	48353653	G	A	SNP	0|0	0|1	36|1	6|13
Chr10	48353944	48353944	A	T	SNP	0|0	0|1	30|1	3|6
Chr10	48354706	48354706	G	T	SNP	0|0	0|1	61|3	6|16
Chr10	48357226	48357226	G	A	SNP	0|1	0|1	38|5	1|7
Chr10	48360130	48360130	T	G	SNP	0|1	0|1	35|3	4|6
Chr10	48360131	48360131	G	A	SNP	0|1	0|1	34|3	4|5
Chr10	48360133	48360133	T	A	SNP	0|1	0|1	33|3	4|5
Chr10	48361583	48361583	G	A	SNP	0|0	0|1	17|0	2|7
Chr10	48361623	48361623	T	C	SNP	0|0	1|1	18|0	0|8
Chr10	48361679	48361679	A	T	SNP	0|0	0|1	28|0	1|12
Chr10	48362089	48362089	C	A	SNP	0|1	0|1	67|6	5|12
Chr10	48362412	48362412	T	C	SNP	0|0	0|1	51|1	4|13
Chr10	48363275	48363275	C	T	SNP	0|0	0|1	19|1	3|11
Chr10	48363756	48363756	A	T	SNP	0|0	0|1	66|3	4|8
Chr10	48364484	48364484	A	G	SNP	0|0	0|1	44|1	5|10
Chr10	48366859	48366859	G	A	SNP	0|1	0|1	41|3	3|5
Chr10	48366926	48366926	G	A	SNP	0|0	0|1	33|0	5|4
Chr10	48369022	48369022	C	T	SNP	0|0	0|1	35|0	8|8
Chr10	48369291	48369291	G	A	SNP	0|0	0|1	63|0	3|8
Chr10	48370133	48370133	C	A	SNP	0|0	0|1	45|3	10|15

Chr10	48370163	48370163	G	A	SNP	0|0	0|1	45|4	11|12
Chr10	48370405	48370405	T	G	SNP	0|1	0|1	61|8	13|18
Chr10	48370461	48370461	T	C	SNP	0|0	0|1	53|3	12|21
Chr10	48370478	48370478	G	A	SNP	0|0	0|1	50|3	11|23
Chr10	48370529	48370529	G	A	SNP	0|0	0|1	42|2	9|23
Chr10	48370596	48370596	A	G	SNP	0|1	0|1	25|2	5|15
Chr10	48370613	48370613	C	G	SNP	0|1	0|1	20|2	3|15
Chr10	48370694	48370694	C	T	SNP	0|1	0|1	33|3	5|15
Chr10	48370874	48370874	G	A	SNP	0|0	0|1	45|1	5|5
Chr10	48370882	48370882	G	A	SNP	0|0	0|1	51|1	4|4
Chr10	48370921	48370921	C	T	SNP	0|0	0|1	57|1	7|6
Chr10	48371650	48371650	T	C	SNP	0|0	0|1	49|0	6|18
Chr10	48373869	48373869	A	T	SNP	0|0	0|1	26|1	1|8
Chr10	48373905	48373905	C	A	SNP	0|0	0|1	25|1	3|7
Chr10	48373911	48373911	A	G	SNP	0|0	0|1	24|1	4|7
Chr10	48374020	48374020	T	C	SNP	0|1	0|1	19|2	9|10
Chr10	48374041	48374041	C	T	SNP	0|1	0|1	19|2	8|12
Chr10	48374079	48374079	G	A	SNP	0|1	0|1	27|2	6|10
Chr10	48374109	48374109	C	T	SNP	0|0	0|1	28|1	8|7
Chr10	48374196	48374196	A	T	SNP	0|0	0|1	40|0	3|5
Chr10	48374223	48374223	G	A	SNP	0|0	0|1	28|0	4|4
Chr10	48374231	48374231	A	G	SNP	0|0	0|1	30|0	6|4
Chr10	48375059	48375059	C	A	SNP	0|0	0|1	15|1	4|5
Chr10	48375334	48375334	T	G	SNP	0|0	0|1	35|2	5|9
Chr10	48375657	48375657	T	A	SNP	0|0	0|1	72|5	4|14
Chr10	48375699	48375699	G	A	SNP	0|0	0|1	69|3	3|11
Chr10	48376493	48376493	A	G	SNP	0|0	0|1	49|3	5|15
Chr10	48380735	48380735	T	C	SNP	0|0	0|1	58|2	4|11
Chr10	48380811	48380811	A	T	SNP	0|0	0|1	42|3	3|5
Chr10	48381563	48381563	C	T	SNP	0|0	0|1	44|1	2|8
Chr10	48382152	48382152	A	T	SNP	0|0	1|1	11|0	0|10
Chr10	48382229	48382229	T	C	SNP	0|0	0|1	33|0	3|11
Chr10	48382292	48382292	C	T	SNP	0|0	0|1	35|0	3|7
Chr10	48382599	48382599	A	G	SNP	0|0	0|1	60|4	7|16
Chr10	48382747	48382747	G	T	SNP	0|1	0|1	48|4	8|20
Chr10	48382874	48382874	T	C	SNP	0|1	0|1	54|7	10|10
Chr10	48382911	48382911	T	C	SNP	0|1	0|1	52|4	8|10
Chr10	48384912	48384912	A	G	SNP	0|0	0|1	35|2	3|13
Chr10	48385158	48385158	C	G	SNP	0|0	0|1	47|2	1|10
Chr10	48388028	48388028	A	T	SNP	0|0	0|1	45|1	3|11
Chr10	48388553	48388553	G	A	SNP	0|0	0|1	56|1	5|20
Chr10	48389149	48389149	A	G	SNP	0|1	0|1	31|4	3|17

Chr10	48391030	48391030	T	C	SNP	0|0	0|1	16|0	1|7
Chr10	48393171	48393171	G	C	SNP	0|0	0|1	37|1	5|12
Chr10	48393727	48393727	A	G	SNP	0|0	0|1	19|0	3|7
Chr10	48393758	48393758	G	A	SNP	0|0	0|1	17|0	3|6
Chr10	48393763	48393763	C	T	SNP	0|0	0|1	16|0	3|6
Chr10	48393804	48393804	G	A	SNP	0|0	0|1	14|0	3|5
Chr10	48393810	48393810	G	A	SNP	0|0	0|1	15|0	3|5
Chr10	48393855	48393855	C	T	SNP	0|0	0|1	18|0	2|6
Chr10	48393875	48393875	G	C	SNP	0|0	0|1	23|0	1|7
Chr10	48393959	48393959	C	T	SNP	0|0	0|1	32|0	4|8
Chr10	48393987	48393987	C	T	SNP	0|0	0|1	29|1	4|8
Chr10	48394001	48394001	C	T	SNP	0|1	0|1	29|3	5|11
Chr10	48394004	48394004	A	G	SNP	0|1	0|1	30|4	5|11
Chr10	48396230	48396230	C	T	SNP	0|0	0|1	50|1	6|9
Chr10	48396273	48396273	A	G	SNP	0|0	0|1	47|1	5|11
Chr10	48398769	48398769	A	T	SNP	0|0	0|1	31|1	8|9
Chr10	48398948	48398948	T	G	SNP	0|0	0|1	30|1	1|12
Chr10	48399130	48399130	T	C	SNP	0|0	0|1	32|1	5|10
Chr10	48399462	48399462	G	T	SNP	0|1	0|1	19|2	2|10
Chr10	48400425	48400425	T	C	SNP	0|0	0|1	26|0	2|6
Chr10	48400891	48400891	C	T	SNP	0|0	0|1	49|0	4|8
Chr10	48400896	48400896	C	A	SNP	0|0	0|1	45|0	4|8
Chr10	48402259	48402259	A	C	SNP	0|0	0|1	49|1	8|13
Chr10	48403178	48403178	T	A	SNP	0|0	0|1	30|0	3|7
Chr10	48405391	48405391	A	C	SNP	0|0	0|1	15|0	2|7
Chr10	48406017	48406017	T	A	SNP	0|0	0|1	31|0	2|6
Chr10	48406123	48406123	G	A	SNP	0|0	0|1	39|1	3|9
Chr10	48406126	48406126	T	C	SNP	0|0	0|1	39|1	4|8
Chr10	48406160	48406160	A	C	SNP	0|0	0|1	34|1	5|8
Chr10	48406171	48406171	T	C	SNP	0|0	0|1	36|1	5|10
Chr10	48406267	48406267	A	G	SNP	0|0	0|1	50|0	6|12
Chr10	48406272	48406272	C	T	SNP	0|0	0|1	52|0	6|12
Chr10	48406297	48406297	C	T	SNP	0|0	0|1	55|1	6|15
Chr10	48406549	48406549	G	T	SNP	0|0	0|1	60|1	6|15
Chr10	48406798	48406798	A	G	SNP	0|0	0|1	48|0	8|14
Chr10	48408367	48408367	A	T	SNP	0|0	0|1	55|0	6|12
Chr10	48408622	48408622	T	C	SNP	0|0	0|1	42|3	5|18
Chr10	48408663	48408663	G	A	SNP	0|1	0|1	49|5	3|19
Chr10	48409246	48409246	T	A	SNP	0|0	0|1	75|2	6|15
Chr10	48409781	48409781	A	C	SNP	0|0	0|1	50|2	8|11
Chr10	48409963	48409963	C	T	SNP	0|0	0|1	34|0	7|12
Chr10	48410228	48410228	T	C	SNP	0|0	0|1	27|1	3|5

Chr10	48410523	48410523	T	A	SNP	0|0	0|1	45|0	2|12
Chr10	48412209	48412209	T	A	SNP	0|0	0|1	68|0	1|12
Chr10	48412835	48412835	G	A	SNP	0|1	0|1	61|5	9|15
Chr10	48412951	48412951	G	A	SNP	0|0	0|1	40|1	5|16
Chr10	48415542	48415542	A	G	SNP	0|0	0|1	10|0	4|5
Chr10	48415988	48415988	C	T	SNP	0|0	0|1	13|0	4|4
Chr10	48416953	48416953	T	G	SNP	0|0	0|1	38|0	2|11
Chr10	48417269	48417269	C	T	SNP	0|0	0|1	42|0	5|13
Chr10	48417417	48417417	C	A	SNP	0|0	0|1	26|0	2|6
Chr10	48417462	48417462	C	G	SNP	0|0	0|1	23|0	2|6
Chr10	48417942	48417942	C	T	SNP	0|0	0|1	30|0	3|8
Chr10	48418354	48418354	G	A	SNP	0|0	0|1	45|2	9|13
Chr10	48418675	48418675	T	C	SNP	0|0	0|1	22|0	3|11
Chr10	48419781	48419781	A	T	SNP	0|0	0|1	46|1	5|14
Chr10	48420097	48420097	A	T	SNP	0|0	0|1	22|0	5|4
Chr10	48420113	48420113	T	C	SNP	0|0	0|1	23|0	5|4
Chr10	48421664	48421664	C	T	SNP	0|0	0|1	14|1	2|10
Chr10	48421781	48421781	A	T	SNP	0|0	0|1	10|0	3|6
Chr10	48421782	48421782	A	T	SNP	0|0	0|1	10|0	3|6
Chr10	48423831	48423831	A	C	SNP	0|1	0|1	73|9	8|11
Chr10	48425086	48425086	C	G	SNP	0|0	0|1	46|3	7|14
Chr10	48425151	48425151	T	C	SNP	0|0	0|1	37|3	9|13
Chr10	48425370	48425370	G	T	SNP	0|0	0|1	24|1	5|14
Chr10	48426462	48426462	A	G	SNP	0|0	0|1	16|1	4|5
Chr10	48426907	48426907	A	T	SNP	0|0	0|1	17|0	1|9
Chr10	48429086	48429086	A	G	SNP	0|0	0|1	15|1	5|3
Chr10	48429306	48429306	G	A	SNP	0|1	0|1	12|1	3|10
Chr10	48430696	48430696	C	A	SNP	0|0	0|1	24|1	2|6
Chr10	48431629	48431629	A	G	SNP	0|0	0|1	9|0	4|4
Chr10	48433526	48433526	A	G	SNP	0|0	0|1	36|1	5|10
Chr10	48433614	48433614	T	G	SNP	0|0	0|1	34|2	11|11
Chr10	48433970	48433970	T	C	SNP	0|1	0|1	28|3	9|4
Chr10	48434455	48434455	C	A	SNP	0|0	0|1	62|4	7|17
Chr10	48435065	48435065	A	G	SNP	0|0	0|1	69|0	10|13
Chr10	48435258	48435258	G	A	SNP	0|0	0|1	54|0	5|15
Chr10	48435384	48435384	A	G	SNP	0|0	0|1	54|0	10|19
Chr10	48435745	48435745	C	T	SNP	0|0	0|1	42|1	11|21
Chr10	48435801	48435801	A	C	SNP	0|0	0|1	44|0	10|16
Chr10	48435961	48435961	G	T	SNP	0|0	0|1	35|1	4|11
Chr10	48435962	48435962	G	C	SNP	0|0	0|1	34|1	4|11
Chr10	48435965	48435965	C	T	SNP	0|0	0|1	34|1	4|11
Chr10	48435995	48435995	A	G	SNP	0|0	1|1	29|1	0|8

Chr10	48436014	48436014	A	G	SNP	0|0	1|1	19|1	0|8
Chr10	48436018	48436018	C	A	SNP	0|0	1|1	18|1	0|9
Chr10	48436019	48436019	A	G	SNP	0|0	1|1	18|1	0|9
Chr10	48436024	48436024	A	G	SNP	0|0	1|1	19|1	0|9
Chr10	48436029	48436029	T	C	SNP	0|0	1|1	19|0	0|9
Chr10	48436040	48436040	G	A	SNP	0|0	0|1	18|0	1|7
Chr10	48436051	48436051	G	A	SNP	0|0	0|1	19|0	1|7
Chr10	48436054	48436054	G	A	SNP	0|0	0|1	19|0	1|7
Chr10	48436063	48436063	A	G	SNP	0|0	0|1	20|0	2|7
Chr10	48436064	48436064	C	T	SNP	0|0	0|1	20|0	2|7
Chr10	48436310	48436310	C	G	SNP	0|0	1|1	32|0	0|10
Chr10	48436329	48436329	T	C	SNP	0|0	1|1	28|0	0|10
Chr10	48436439	48436439	A	T	SNP	0|0	0|1	22|0	2|9
Chr10	48437811	48437811	T	A	SNP	0|0	0|1	26|1	3|15
Chr10	48437999	48437999	T	G	SNP	0|0	0|1	40|0	5|20
Chr10	48439199	48439199	A	G	SNP	0|0	0|1	45|1	3|10
Chr10	48443507	48443507	G	A	SNP	0|0	1|1	41|1	0|12
Chr10	48443701	48443701	T	C	SNP	0|1	1|1	11|1	0|9
Chr10	48447777	48447777	C	T	SNP	0|0	0|1	50|0	6|4
Chr10	48447790	48447790	T	C	SNP	0|0	0|1	49|0	8|6
Chr10	48448066	48448066	T	C	SNP	0|0	0|1	23|0	2|10
Chr10	48448324	48448324	T	A	SNP	0|0	0|1	34|2	1|18
Chr10	48448402	48448402	T	A	SNP	0|0	0|1	35|1	3|13
Chr10	48448712	48448712	T	C	SNP	0|0	0|1	51|3	4|18
Chr10	48449596	48449596	G	A	SNP	0|0	0|1	47|4	9|35
Chr10	48450184	48450184	G	A	SNP	0|0	0|1	30|1	5|9
Chr10	48450501	48450501	T	C	SNP	0|0	0|1	14|1	2|6
Chr10	48450506	48450506	C	G	SNP	0|0	0|1	14|1	2|6
Chr10	48450518	48450518	A	G	SNP	0|0	0|1	14|1	2|6
Chr10	48450521	48450521	C	A	SNP	0|0	0|1	14|1	2|6
Chr10	48450524	48450524	G	A	SNP	0|0	0|1	14|1	2|6
Chr10	48451344	48451344	C	T	SNP	0|0	0|1	19|0	3|5
Chr10	48451414	48451414	A	G	SNP	0|0	0|1	19|0	3|5
Chr10	48451825	48451825	G	T	SNP	0|0	0|1	49|3	6|6
Chr10	48451942	48451942	T	G	SNP	0|0	0|1	56|4	4|14
Chr10	48452314	48452314	A	G	SNP	0|0	0|1	59|2	2|7
Chr10	48452899	48452899	C	T	SNP	0|0	0|1	29|1	3|11
Chr10	48453204	48453204	G	C	SNP	0|0	0|1	35|1	6|9
Chr10	48454151	48454151	A	G	SNP	0|0	0|1	40|1	6|4
Chr10	48454363	48454363	G	A	SNP	0|1	0|1	40|3	10|13
Chr10	48454528	48454528	C	T	SNP	0|0	0|1	42|2	7|6
Chr10	48454582	48454582	T	G	SNP	0|0	0|1	48|1	7|3

Chr10	48454599	48454599	A	G	SNP	0|0	0|1	43|1	8|5
Chr10	48454619	48454619	C	T	SNP	0|0	0|1	43|1	8|5
Chr10	48454655	48454655	T	C	SNP	0|0	0|1	43|0	6|9
Chr10	48454662	48454662	G	A	SNP	0|0	0|1	45|0	5|10
Chr10	48454668	48454668	A	G	SNP	0|0	0|1	46|0	5|10
Chr10	48454738	48454738	G	A	SNP	0|0	0|1	48|3	4|18
Chr10	48454751	48454751	C	T	SNP	0|0	0|1	51|3	4|17
Chr10	48455258	48455258	T	C	SNP	0|0	0|1	55|0	8|9
Chr10	48458047	48458047	C	T	SNP	0|0	0|1	34|1	2|10
Chr10	48458419	48458419	T	C	SNP	0|0	0|1	51|2	5|9
Chr10	48458616	48458616	G	A	SNP	0|0	0|1	46|1	6|14
Chr10	48459040	48459040	A	G	SNP	0|0	0|1	42|0	5|14
Chr10	48460027	48460027	G	A	SNP	0|1	0|1	41|4	9|12
Chr10	48460266	48460266	T	C	SNP	0|0	0|1	41|3	9|13
Chr10	48462915	48462915	C	T	SNP	0|0	0|1	57|2	4|21
Chr10	48463777	48463777	C	T	SNP	0|0	0|1	38|0	4|10
Chr10	48463921	48463921	C	T	SNP	0|0	0|1	30|1	3|9
Chr10	48463984	48463984	T	C	SNP	0|0	0|1	25|1	1|7
Chr10	48464140	48464140	A	C	SNP	0|0	0|1	25|1	4|6
Chr10	48465319	48465319	T	A	SNP	0|0	0|1	68|2	5|11
Chr10	48467024	48467024	A	G	SNP	0|1	0|1	40|5	7|8
Chr10	48467123	48467123	A	G	SNP	0|1	0|1	57|6	3|6
Chr10	48468755	48468755	A	G	SNP	0|0	0|1	18|0	1|7
Chr10	48468907	48468907	C	G	SNP	0|0	0|1	25|1	1|7
Chr10	48469020	48469020	C	T	SNP	0|0	0|1	28|2	5|14
Chr10	48469148	48469148	G	A	SNP	0|0	1|1	55|1	0|17
Chr10	48470244	48470244	C	T	SNP	0|0	1|1	23|0	0|13
Chr10	48470492	48470492	T	G	SNP	0|0	1|1	24|0	0|9
Chr10	48470795	48470795	A	C	SNP	0|0	0|1	28|1	5|12
Chr10	48471485	48471485	T	C	SNP	0|0	0|1	53|2	5|17
Chr10	48471532	48471532	C	T	SNP	0|0	0|1	49|2	6|16
Chr10	48471676	48471676	C	T	SNP	0|0	0|1	50|2	4|17
Chr10	48471683	48471683	C	A	SNP	0|0	0|1	51|2	4|17
Chr10	48472140	48472140	T	A	SNP	0|0	0|1	30|0	5|13
Chr10	48472413	48472413	A	C	SNP	0|0	0|1	44|5	5|7
Chr10	48473100	48473100	A	G	SNP	0|0	0|1	42|0	2|7
Chr10	48473496	48473496	C	T	SNP	0|0	0|1	54|1	2|13
Chr10	48473597	48473597	C	A	SNP	0|0	0|1	47|2	5|6
Chr10	48473598	48473598	G	A	SNP	0|0	0|1	46|2	5|6
Chr10	48473658	48473658	G	T	SNP	0|0	0|1	52|2	7|5
Chr10	48473829	48473829	G	A	SNP	0|0	0|1	37|1	6|5
Chr10	48474197	48474197	C	G	SNP	0|1	0|1	36|6	3|13

Chr10	48474422	48474422	C	A	SNP	0|0	0|1	35|0	3|11
Chr10	48474586	48474586	A	G	SNP	0|0	0|1	61|1	10|13
Chr10	48475051	48475051	C	T	SNP	0|0	0|1	57|1	5|12
Chr10	48478016	48478016	T	G	SNP	0|0	0|1	68|1	5|14
Chr10	48478210	48478210	A	G	SNP	0|0	0|1	55|1	3|6
Chr10	48478291	48478291	A	G	SNP	0|0	0|1	55|0	2|6
Chr10	48478595	48478595	C	T	SNP	0|0	0|1	60|2	11|19
Chr10	48478950	48478950	G	T	SNP	0|0	0|1	46|4	10|20
Chr10	48479004	48479004	T	C	SNP	0|0	0|1	45|2	10|11
Chr10	48479396	48479396	A	G	SNP	0|0	0|1	43|0	6|13
Chr10	48480572	48480572	T	C	SNP	0|0	0|1	22|0	5|10
Chr10	48481730	48481730	C	A	SNP	0|0	0|1	56|2	6|5
Chr10	48482214	48482214	C	T	SNP	0|0	0|1	46|0	7|9
Chr10	48482566	48482566	A	G	SNP	0|0	0|1	29|0	4|8
Chr10	48484588	48484588	A	G	SNP	0|0	0|1	30|2	1|11
Chr10	48484619	48484619	G	A	SNP	0|0	0|1	34|1	1|8
Chr10	48484634	48484634	G	A	SNP	0|0	0|1	30|1	1|8
Chr10	48484646	48484646	G	T	SNP	0|0	0|1	31|2	1|10
Chr10	48485207	48485207	G	T	SNP	0|1	0|1	19|3	6|9
Chr10	48485237	48485237	A	G	SNP	0|1	0|1	20|5	5|12
Chr10	48485273	48485273	A	G	SNP	0|1	0|1	28|4	7|20
Chr10	48486508	48486508	G	A	SNP	0|0	0|1	48|1	4|26
Chr10	48486809	48486809	C	G	SNP	0|0	0|1	42|0	7|14
Chr10	48486907	48486907	G	T	SNP	0|0	0|1	47|1	5|10
Chr10	48487431	48487431	G	T	SNP	0|0	0|1	47|4	5|14
Chr10	48488022	48488022	A	G	SNP	0|0	0|1	44|0	2|13
Chr10	48488457	48488457	C	T	SNP	0|1	0|1	37|3	2|12
Chr10	48488605	48488605	A	T	SNP	0|0	0|1	23|1	1|8
Chr10	48490035	48490035	T	G	SNP	0|0	1|1	54|0	0|9
Chr10	48490069	48490069	T	A	SNP	0|0	0|1	54|0	1|9
Chr10	48490180	48490180	A	G	SNP	0|0	0|1	62|1	5|10
Chr10	48490571	48490571	T	C	SNP	0|0	0|1	65|0	11|18
Chr10	48490940	48490940	G	C	SNP	0|0	0|1	46|2	2|7
Chr10	48491171	48491171	T	C	SNP	0|1	0|1	60|5	3|11
Chr10	48491737	48491737	C	A	SNP	0|0	0|1	32|1	16|16
Chr10	48491882	48491882	T	C	SNP	0|0	0|1	67|2	5|10
Chr10	48494123	48494123	G	A	SNP	0|0	0|1	36|1	3|11
Chr10	48494281	48494281	A	G	SNP	0|0	0|1	20|0	6|7
Chr10	48494312	48494312	C	A	SNP	0|0	0|1	19|0	7|6
Chr10	48495385	48495385	G	A	SNP	0|0	0|1	28|1	7|11
Chr10	48495392	48495392	T	A	SNP	0|0	0|1	27|1	6|12
Chr10	48495461	48495461	G	A	SNP	0|0	0|1	38|1	6|10

Chr10	48495722	48495722	G	A	SNP	0|0	0|1	58|1	6|21
Chr10	48495813	48495813	A	G	SNP	0|0	0|1	64|1	3|17
Chr10	48495838	48495838	T	A	SNP	0|0	0|1	63|1	3|23
Chr10	48496101	48496101	C	G	SNP	0|0	0|1	57|0	4|29
Chr10	48496166	48496166	C	T	SNP	0|0	0|1	55|0	3|26
Chr10	48496276	48496276	G	A	SNP	0|0	0|1	48|3	4|12
Chr10	48496417	48496417	C	A	SNP	0|0	0|1	50|4	7|8
Chr10	48496613	48496613	G	A	SNP	0|0	0|1	47|3	5|17
Chr10	48496765	48496765	C	T	SNP	0|0	0|1	61|3	7|15
Chr10	48496995	48496995	C	G	SNP	0|0	0|1	68|1	8|13
Chr10	48499076	48499076	C	T	SNP	0|0	0|1	56|1	8|21
Chr10	48499476	48499476	T	C	SNP	0|0	0|1	52|0	6|8
Chr10	48499565	48499565	G	C	SNP	0|0	0|1	65|1	8|18
Chr10	48499673	48499673	A	G	SNP	0|0	0|1	68|1	3|29
Chr10	48499894	48499894	A	G	SNP	0|0	0|1	51|1	10|6
Chr10	48500401	48500401	G	C	SNP	0|1	0|1	31|6	8|6
Chr10	48500530	48500530	T	A	SNP	0|0	0|1	10|0	1|8
Chr10	48501510	48501510	T	G	SNP	0|0	0|1	24|1	1|9
Chr10	48501945	48501945	G	T	SNP	0|0	0|1	28|2	4|8
Chr10	48502008	48502008	A	C	SNP	0|0	0|1	34|2	4|9
Chr10	48502278	48502278	G	A	SNP	0|0	1|1	35|2	0|8
Chr10	48502393	48502393	C	T	SNP	0|1	0|1	28|4	3|15
Chr10	48502457	48502457	A	G	SNP	0|0	0|1	39|2	3|19
Chr10	48502722	48502722	T	A	SNP	0|0	0|1	39|0	5|17
Chr10	48503156	48503156	C	T	SNP	1|1	0|1	1|60	17|10
Chr10	48503335	48503335	G	A	SNP	0|0	0|1	50|0	10|15
Chr10	48505000	48505000	A	T	SNP	0|0	0|1	39|1	2|7
Chr10	48505073	48505073	G	A	SNP	0|0	0|1	36|2	4|8
Chr10	48505132	48505132	A	T	SNP	0|0	0|1	27|1	6|6
Chr10	48505294	48505294	T	G	SNP	0|1	0|1	25|7	7|5
Chr10	48505456	48505456	G	A	SNP	0|0	0|1	24|0	4|10
Chr10	48505506	48505506	G	C	SNP	0|0	0|1	32|1	4|8
Chr10	48507744	48507744	A	T	SNP	0|0	0|1	59|0	4|18
Chr10	48508070	48508070	T	A	SNP	0|0	0|1	53|1	4|10
Chr10	48509147	48509147	T	C	SNP	0|0	0|1	51|1	11|18
Chr10	48509637	48509637	A	T	SNP	0|1	0|1	21|2	4|14
Chr10	48509684	48509684	G	A	SNP	0|0	0|1	16|1	3|7
Chr10	48509753	48509753	A	T	SNP	0|1	0|1	11|1	2|8
Chr10	48509781	48509781	A	T	SNP	0|0	0|1	12|0	3|9
Chr10	48509857	48509857	C	A	SNP	0|0	0|1	19|0	3|12
Chr10	48509930	48509930	C	T	SNP	0|0	0|1	16|1	1|8
Chr10	48510457	48510457	A	T	SNP	0|0	0|1	13|0	1|8

Chr10	48511551	48511551	A	G	SNP	0|0	0|1	9|0	2|6
Chr10	48511575	48511575	C	A	SNP	0|0	0|1	14|0	2|8
Chr10	48511582	48511582	A	T	SNP	0|0	0|1	14|0	2|8
Chr10	48511720	48511720	T	A	SNP	0|0	0|1	16|0	5|3
Chr10	48511745	48511745	A	G	SNP	0|0	0|1	14|0	3|6
Chr10	48511801	48511801	C	T	SNP	0|0	0|1	16|0	2|6
Chr10	48520944	48520944	C	G	SNP	0|0	0|1	54|1	4|16
Chr10	48524424	48524424	T	C	SNP	0|1	0|1	11|1	6|7
Chr10	48524444	48524444	A	G	SNP	0|0	0|1	8|0	5|3
Chr10	48525015	48525015	A	G	SNP	0|0	0|1	30|1	3|7
Chr10	48525026	48525026	G	A	SNP	0|0	0|1	27|1	3|6
Chr10	48525039	48525039	G	C	SNP	0|0	0|1	25|1	3|6
Chr10	48526864	48526864	A	C	SNP	0|1	0|1	17|2	4|5
Chr10	48527000	48527000	T	C	SNP	0|1	0|1	23|2	4|4
Chr10	48527644	48527644	C	T	SNP	0|0	0|1	9|0	4|6
Chr10	48528314	48528314	T	C	SNP	0|0	0|1	13|0	2|7
Chr10	48528334	48528334	G	C	SNP	0|0	0|1	15|0	2|7
Chr10	48529549	48529549	G	C	SNP	0|0	0|1	21|0	1|8
Chr10	48529626	48529626	A	C	SNP	0|0	0|1	21|0	2|6
Chr10	48529867	48529867	A	G	SNP	0|0	0|1	11|0	1|10
Chr10	48531018	48531018	A	T	SNP	0|0	0|1	14|1	6|3
Chr10	48532363	48532363	A	C	SNP	0|0	0|1	59|4	10|19
Chr10	48534394	48534394	T	C	SNP	0|0	0|1	44|1	6|8
Chr10	48534664	48534664	A	T	SNP	0|0	0|1	44|1	6|10
Chr10	48535953	48535953	G	T	SNP	0|1	0|1	9|6	6|6
Chr10	48535998	48535998	A	G	SNP	0|1	0|1	8|4	4|5
Chr10	48538214	48538214	A	G	SNP	0|1	0|1	12|1	3|6
Chr10	48538506	48538506	A	G	SNP	0|0	0|1	53|2	4|9
Chr10	48538906	48538906	C	G	SNP	0|1	0|1	66|7	9|16
Chr10	48539374	48539374	T	C	SNP	0|0	0|1	31|2	1|9
Chr10	48539606	48539606	C	T	SNP	0|0	0|1	48|2	5|9
Chr10	48539930	48539930	T	A	SNP	0|0	0|1	51|2	6|14
Chr10	48540387	48540387	A	G	SNP	0|0	0|1	58|1	5|17
Chr10	48540583	48540583	G	A	SNP	0|0	0|1	58|1	5|13
Chr10	48541003	48541003	C	A	SNP	0|1	0|1	51|4	11|19
Chr10	48541087	48541087	C	T	SNP	0|1	0|1	53|4	6|16
Chr10	48541088	48541088	A	T	SNP	0|1	0|1	53|4	6|15
Chr10	48541149	48541149	C	A	SNP	0|0	0|1	62|4	8|7
Chr10	48541155	48541155	C	A	SNP	0|0	0|1	63|4	7|6
Chr10	48542412	48542412	T	G	SNP	0|0	0|1	62|3	10|18
Chr10	48542759	48542759	T	A	SNP	0|0	0|1	44|0	1|10
Chr10	48542934	48542934	C	A	SNP	0|0	0|1	38|0	10|9

Chr10	48543055	48543055	A	T	SNP	0|0	0|1	48|3	6|10
Chr10	48543168	48543168	T	C	SNP	0|0	0|1	59|1	8|14
Chr10	48545212	48545212	A	G	SNP	0|1	0|1	27|3	1|8
Chr10	48545250	48545250	C	T	SNP	0|1	0|1	27|5	4|10
Chr10	48545296	48545296	A	G	SNP	0|1	0|1	37|5	7|9
Chr10	48545426	48545426	T	A	SNP	0|0	0|1	34|1	6|12
Chr10	48546907	48546907	T	G	SNP	0|0	0|1	43|3	8|8
Chr10	48547482	48547482	T	C	SNP	0|1	0|1	12|2	3|5
Chr10	48547506	48547506	A	G	SNP	0|1	0|1	16|2	3|5
Chr10	48547858	48547858	C	G	SNP	0|0	0|1	14|1	3|9
Chr10	48547944	48547944	G	A	SNP	0|1	0|1	32|3	3|9
Chr10	48548107	48548107	C	T	SNP	0|0	0|1	42|0	5|5
Chr10	48549239	48549239	T	A	SNP	0|1	0|1	15|4	6|4
Chr10	48549240	48549240	T	A	SNP	0|1	0|1	15|4	6|4
Chr10	48549257	48549257	T	A	SNP	0|1	0|1	12|4	5|3
Chr10	48549302	48549302	A	G	SNP	0|1	0|1	8|5	5|3
Chr10	48549378	48549378	T	C	SNP	0|1	0|1	10|2	2|7
Chr10	48551211	48551211	T	C	SNP	0|0	0|1	30|2	6|13
Chr10	48551502	48551502	G	T	SNP	0|1	0|1	32|3	4|11
Chr10	48551531	48551531	T	G	SNP	0|1	0|1	32|3	4|7
Chr10	48551669	48551669	A	C	SNP	0|1	0|1	31|6	10|9
Chr10	48552047	48552047	C	T	SNP	0|0	0|1	55|2	3|19
Chr10	48553874	48553874	T	G	SNP	0|0	0|1	32|2	6|15
Chr10	48554855	48554855	C	T	SNP	0|1	0|1	53|6	4|15
Chr10	48554867	48554867	T	A	SNP	0|1	0|1	54|5	4|18
Chr10	48554982	48554982	T	C	SNP	0|1	0|1	43|6	3|10
Chr10	48555498	48555498	T	C	SNP	0|1	0|1	42|5	3|24
Chr10	48555669	48555669	T	G	SNP	0|0	1|1	57|4	0|20
Chr10	48555796	48555796	T	C	SNP	0|0	0|1	51|0	3|23
Chr10	48556422	48556422	T	G	SNP	0|0	0|1	29|4	6|8
Chr10	48556874	48556874	C	G	SNP	0|1	0|1	19|2	5|9
Chr10	48557012	48557012	G	A	SNP	0|0	0|1	31|3	9|8
Chr10	48557992	48557992	T	G	SNP	0|0	0|1	47|2	9|18
Chr10	48558227	48558227	G	A	SNP	0|1	0|1	37|3	10|9
Chr10	48558263	48558263	G	A	SNP	0|1	0|1	45|4	13|11
Chr10	48558264	48558264	C	T	SNP	0|1	0|1	45|4	13|10
Chr10	48558887	48558887	C	G	SNP	0|0	0|1	58|3	5|11
Chr10	48559301	48559301	T	A	SNP	0|0	0|1	47|3	11|9
Chr10	48559507	48559507	G	T	SNP	0|0	0|1	39|2	4|5
Chr10	48559521	48559521	G	A	SNP	0|0	0|1	42|2	4|4
Chr10	48559712	48559712	A	T	SNP	0|0	0|1	25|0	2|18
Chr10	48560007	48560007	C	A	SNP	0|1	0|1	35|5	7|9

Chr10	48560412	48560412	A	G	SNP	0|0	0|1	29|1	3|11
Chr10	48560462	48560462	C	T	SNP	0|0	0|1	37|0	3|10
Chr10	48560807	48560807	T	C	SNP	0|1	0|1	22|2	4|7
Chr10	48561041	48561041	A	G	SNP	0|0	0|1	19|0	4|4
Chr10	48561152	48561152	C	T	SNP	0|0	0|1	33|1	3|5
Chr10	48561158	48561158	C	T	SNP	0|0	0|1	33|1	3|5
Chr10	48561181	48561181	G	T	SNP	0|0	0|1	42|1	2|6
Chr10	48561463	48561463	C	A	SNP	0|1	1|1	37|3	0|10
Chr10	48561514	48561514	A	T	SNP	0|1	1|1	38|11	0|13
Chr10	48561563	48561563	G	T	SNP	0|1	1|1	38|8	0|12
Chr10	48562471	48562471	T	C	SNP	0|0	1|1	28|1	0|16
Chr10	48562747	48562747	G	A	SNP	0|0	0|1	31|1	1|7
Chr10	48564401	48564401	A	G	SNP	0|1	0|1	19|2	3|6
Chr10	48564575	48564575	C	T	SNP	0|0	0|1	60|0	4|8
Chr10	48564613	48564613	T	C	SNP	0|0	0|1	72|1	8|13
Chr10	48565010	48565010	A	G	SNP	0|0	0|1	49|5	10|7
Chr10	48565688	48565688	C	T	SNP	0|0	0|1	55|1	6|14
Chr10	48565824	48565824	T	C	SNP	0|0	0|1	40|2	11|12
Chr10	48566023	48566023	G	A	SNP	0|0	0|1	33|1	8|8
Chr10	48566337	48566337	T	A	SNP	0|1	0|1	46|4	3|5
Chr10	48567444	48567444	C	T	SNP	0|0	0|1	40|2	9|12
Chr10	48567762	48567762	A	T	SNP	0|1	0|1	42|4	5|12
Chr10	48568852	48568852	C	G	SNP	0|0	0|1	46|1	7|13
Chr10	48569085	48569085	A	C	SNP	0|1	0|1	55|9	5|11
Chr10	48574224	48574224	G	A	SNP	0|1	0|1	52|5	3|16
Chr10	48574326	48574326	G	T	SNP	0|0	0|1	57|4	3|15
Chr10	48574545	48574545	A	T	SNP	0|1	0|1	55|4	2|6
Chr10	48574617	48574617	G	A	SNP	0|1	0|1	58|5	6|8
Chr10	48574651	48574651	C	T	SNP	0|1	0|1	58|5	5|8
Chr10	48574653	48574653	A	T	SNP	0|1	0|1	58|5	5|8
Chr10	48574664	48574664	A	T	SNP	0|0	0|1	58|4	5|7
Chr10	48574707	48574707	C	T	SNP	0|1	0|1	66|7	5|7
Chr10	48574795	48574795	A	G	SNP	0|1	0|1	75|11	5|11
Chr10	48574932	48574932	G	T	SNP	0|0	0|1	43|1	7|23
Chr10	48575078	48575078	G	A	SNP	0|0	0|1	71|4	6|22
Chr10	48575326	48575326	G	A	SNP	0|1	0|1	56|8	5|19
Chr10	48575724	48575724	T	C	SNP	0|1	0|1	46|5	10|6
Chr10	48575926	48575926	T	A	SNP	0|0	0|1	43|2	4|9
Chr10	48576106	48576106	A	T	SNP	0|1	0|1	41|4	4|8
Chr10	48576256	48576256	C	A	SNP	0|0	0|1	24|1	1|8
Chr10	48576647	48576647	A	G	SNP	0|1	0|1	36|4	2|7
Chr10	48577965	48577965	G	A	SNP	0|0	0|1	31|2	4|7

Chr10	48578239	48578239	G	A	SNP	0|0	0|1	45|3	3|12
Chr10	48578329	48578329	C	A	SNP	0|0	0|1	45|2	3|8
Chr10	48578340	48578340	C	A	SNP	0|0	0|1	45|2	3|8
Chr10	48578373	48578373	A	G	SNP	0|0	0|1	48|2	6|9
Chr10	48578418	48578418	C	T	SNP	0|0	0|1	49|3	4|13
Chr10	48578469	48578469	T	A	SNP	0|0	0|1	45|2	5|18
Chr10	48578503	48578503	T	C	SNP	0|0	0|1	44|3	3|17
Chr10	48578650	48578650	G	A	SNP	0|0	0|1	47|1	6|5
Chr10	48578695	48578695	T	C	SNP	0|0	0|1	44|0	9|10
Chr10	48578957	48578957	A	T	SNP	0|1	0|1	40|11	5|16
Chr10	48579095	48579095	A	G	SNP	0|1	0|1	58|7	6|14
Chr10	48579207	48579207	T	C	SNP	0|1	0|1	43|11	6|20
Chr10	48579484	48579484	A	G	SNP	0|0	0|1	46|0	16|15
Chr10	48579547	48579547	T	A	SNP	0|0	0|1	44|1	10|9
Chr10	48579548	48579548	T	A	SNP	0|0	0|1	44|1	10|9
Chr10	48579584	48579584	G	A	SNP	0|0	0|1	46|0	7|8
Chr10	48579688	48579688	A	C	SNP	0|0	0|1	64|2	2|20
Chr10	48580671	48580671	G	C	SNP	0|0	0|1	9|0	3|5
Chr10	48580737	48580737	G	A	SNP	0|0	0|1	15|0	4|5
Chr10	48580740	48580740	A	G	SNP	0|0	0|1	13|0	4|5
Chr10	48580744	48580744	G	C	SNP	0|0	0|1	13|0	4|5
Chr10	48580750	48580750	T	G	SNP	0|0	0|1	11|0	5|7
Chr10	48580765	48580765	G	A	SNP	0|0	0|1	13|0	4|7
Chr10	48580781	48580781	G	T	SNP	0|0	0|1	17|0	4|7
Chr10	48580784	48580784	A	C	SNP	0|0	0|1	17|0	4|7
Chr10	48580785	48580785	C	T	SNP	0|0	0|1	17|0	4|7
Chr10	48580879	48580879	C	T	SNP	0|0	0|1	14|0	1|7
Chr10	48582698	48582698	C	G	SNP	0|0	0|1	25|0	2|10
Chr10	48582701	48582701	C	T	SNP	0|0	0|1	25|0	2|11
Chr10	48582757	48582757	A	G	SNP	0|0	0|1	39|1	3|18
Chr10	48582759	48582759	A	G	SNP	0|0	0|1	38|1	3|19
Chr10	48582818	48582818	G	A	SNP	0|0	0|1	50|2	7|20
Chr10	48582873	48582873	T	C	SNP	0|0	0|1	49|1	7|12
Chr10	48582902	48582902	T	A	SNP	0|0	0|1	33|1	6|13
Chr10	48582926	48582926	T	A	SNP	0|0	0|1	29|1	6|13
Chr10	48582930	48582930	G	C	SNP	0|0	0|1	29|1	6|13
Chr10	48582940	48582940	C	T	SNP	0|0	0|1	30|1	2|14
Chr10	48583021	48583021	C	T	SNP	0|0	0|1	33|1	1|13
Chr10	48584004	48584004	C	T	SNP	0|0	0|1	30|1	5|11
Chr10	48584173	48584173	A	G	SNP	0|0	1|1	41|1	1|17
Chr10	48585133	48585133	A	G	SNP	0|1	0|1	37|4	4|4
Chr10	48585651	48585651	T	A	SNP	0|1	0|1	32|6	5|14

Chr10	48585791	48585791	G	A	SNP	0|1	0|1	31|8	3|14
Chr10	48586356	48586356	C	T	SNP	0|1	0|1	30|9	6|20
Chr10	48586491	48586491	A	G	SNP	0|1	0|1	29|3	3|8
Chr10	48587139	48587139	T	G	SNP	0|0	0|1	46|4	5|21
Chr10	48587654	48587654	T	A	SNP	0|0	0|1	45|1	5|12
Chr10	48587704	48587704	A	T	SNP	0|0	0|1	44|2	5|13
Chr10	48589382	48589382	T	C	SNP	0|0	0|1	32|2	4|6
Chr10	48589700	48589700	T	C	SNP	0|1	0|1	51|6	4|10
Chr10	48589747	48589747	A	G	SNP	0|0	0|1	59|3	3|10
Chr10	48589835	48589835	G	T	SNP	0|1	0|1	59|8	1|15
Chr10	48590007	48590007	T	C	SNP	0|0	0|1	70|4	5|22
Chr10	48590347	48590347	C	A	SNP	0|1	0|1	66|6	3|17
Chr10	48590800	48590800	A	G	SNP	0|0	0|1	38|1	7|12
Chr10	48591636	48591636	C	G	SNP	0|0	0|1	54|1	3|8
Chr10	48591680	48591680	A	G	SNP	0|1	0|1	53|4	5|7
Chr10	48591874	48591874	C	T	SNP	0|0	0|1	63|5	6|18
Chr10	48592005	48592005	C	A	SNP	0|0	0|1	65|2	9|19
Chr10	48592116	48592116	G	A	SNP	0|0	0|1	48|2	6|9
Chr10	48592311	48592311	C	T	SNP	0|1	0|1	51|5	7|28
Chr10	48592799	48592799	G	C	SNP	0|0	0|1	41|1	13|7
Chr10	48593398	48593398	C	T	SNP	0|0	1|1	32|1	1|15
Chr10	48594388	48594388	T	C	SNP	0|0	0|1	60|2	7|16
Chr10	48594479	48594479	G	A	SNP	0|0	0|1	59|3	12|17
Chr10	48595049	48595049	T	A	SNP	0|0	0|1	48|4	4|14
Chr10	48596313	48596313	A	G	SNP	0|0	0|1	48|3	9|13
Chr10	48596630	48596630	T	G	SNP	0|0	0|1	51|2	6|8
Chr10	48596702	48596702	T	C	SNP	0|0	0|1	65|3	3|9
Chr10	48596870	48596870	G	A	SNP	0|1	0|1	35|5	12|9
Chr10	48597791	48597791	T	C	SNP	0|0	0|1	47|2	14|14
Chr10	48597793	48597793	A	G	SNP	0|0	0|1	48|2	14|14
Chr10	48598942	48598942	G	T	SNP	0|1	0|1	37|8	7|19
Chr10	48599387	48599387	G	A	SNP	0|0	0|1	47|0	4|8
Chr10	48599633	48599633	G	A	SNP	0|0	0|1	36|1	8|22
Chr10	48599694	48599694	A	T	SNP	0|0	0|1	42|2	9|12
Chr10	48599733	48599733	C	G	SNP	0|0	0|1	50|3	5|14
Chr10	48600639	48600639	T	C	SNP	0|0	0|1	42|3	3|15
Chr10	48601471	48601471	C	G	SNP	0|0	0|1	24|2	9|9
Chr10	48602232	48602232	C	G	SNP	0|0	0|1	52|2	4|11
Chr10	48602388	48602388	G	A	SNP	0|0	0|1	34|3	4|11
Chr10	48603758	48603758	C	G	SNP	0|1	0|1	44|5	9|18
Chr10	48604811	48604811	T	G	SNP	0|1	0|1	44|8	7|13
Chr10	48607157	48607157	T	G	SNP	0|1	0|1	25|3	4|8

Chr10	48608052	48608052	C	T	SNP	0|0	0|1	40|0	6|10
Chr10	48608110	48608110	A	T	SNP	0|0	0|1	51|0	8|10
Chr10	48608322	48608322	T	A	SNP	0|1	0|1	63|8	4|20
Chr10	48608746	48608746	A	G	SNP	0|0	0|1	57|2	7|6
Chr10	48609669	48609669	G	T	SNP	0|0	0|1	26|1	3|8
Chr10	48611372	48611372	G	A	SNP	0|1	0|1	35|5	11|6
Chr10	48611376	48611376	G	A	SNP	0|1	0|1	37|5	11|6
Chr10	48612161	48612161	G	A	SNP	0|1	0|1	46|4	6|19
Chr10	48612934	48612934	T	A	SNP	0|1	0|1	33|14	3|16
Chr10	48612992	48612992	T	C	SNP	0|1	0|1	33|10	4|10
Chr10	48613175	48613175	T	A	SNP	0|1	0|1	13|4	5|5
Chr10	48613389	48613389	A	G	SNP	0|0	0|1	49|3	4|11
Chr10	48613841	48613841	T	C	SNP	0|0	0|1	12|0	5|4
Chr10	48614628	48614628	A	G	SNP	0|0	0|1	21|1	7|3
Chr10	48614722	48614722	G	A	SNP	0|1	0|1	27|2	5|5
Chr10	48614760	48614760	T	C	SNP	0|0	0|1	18|1	5|4
Chr10	48615726	48615726	G	T	SNP	0|0	0|1	25|1	3|7
Chr10	48615727	48615727	T	G	SNP	0|0	0|1	25|1	3|7
Chr10	48615748	48615748	G	A	SNP	0|1	0|1	25|2	4|13
Chr10	48615972	48615972	C	G	SNP	0|1	0|1	27|10	8|11
Chr10	48617466	48617466	A	G	SNP	0|0	0|1	55|1	9|20
Chr10	48617483	48617483	G	A	SNP	0|0	0|1	51|3	9|17
Chr10	48618756	48618756	A	G	SNP	0|0	0|1	49|2	10|19
Chr10	48620860	48620860	C	T	SNP	0|0	1|1	36|2	0|8
Chr10	48621413	48621413	G	C	SNP	0|0	0|1	39|2	5|4
Chr10	48625708	48625708	A	C	SNP	0|1	0|1	8|3	1|7
Chr10	48625915	48625915	G	A	SNP	0|0	0|1	26|1	1|8
Chr10	48626126	48626126	T	A	SNP	0|0	0|1	32|0	6|13
Chr10	48626139	48626139	G	A	SNP	0|0	0|1	39|0	6|11
Chr10	48626281	48626281	G	A	SNP	0|1	0|1	53|4	3|6
Chr10	48626597	48626597	A	G	SNP	0|1	0|1	58|7	6|14
Chr10	48626642	48626642	C	A	SNP	0|1	0|1	56|9	6|13
Chr10	48627186	48627186	G	C	SNP	0|0	0|1	77|4	14|11
Chr10	48627656	48627656	T	G	SNP	0|0	0|1	82|1	5|14
Chr10	48627850	48627850	C	G	SNP	0|0	0|1	68|3	10|9
Chr10	48627986	48627986	A	T	SNP	0|0	0|1	53|1	7|18
Chr10	48630786	48630786	C	A	SNP	0|0	0|1	44|2	6|8
Chr10	48630826	48630826	T	C	SNP	0|0	0|1	42|2	6|10
Chr10	48630884	48630884	C	G	SNP	0|1	0|1	38|4	8|13
Chr10	48631055	48631055	G	A	SNP	0|1	0|1	25|5	7|9
Chr10	48631108	48631108	A	C	SNP	0|1	0|1	14|5	7|9
Chr10	48631115	48631115	A	G	SNP	0|1	0|1	14|5	7|9

Chr10	48631178	48631178	C	A	SNP	0|1	0|1	9|2	3|16
Chr10	48631222	48631222	T	A	SNP	0|0	0|1	14|1	2|17
Chr10	48631250	48631250	A	G	SNP	0|0	0|1	21|1	2|21
Chr10	48631298	48631298	A	T	SNP	0|0	0|1	29|1	4|25
Chr10	48632123	48632123	G	C	SNP	0|0	0|1	21|1	3|7
Chr10	48632719	48632719	T	A	SNP	0|0	0|1	11|0	5|9
Chr10	48632724	48632724	T	A	SNP	0|0	0|1	11|0	5|9
Chr10	48632792	48632792	G	T	SNP	0|0	0|1	30|2	5|9
Chr10	48633172	48633172	G	A	SNP	0|0	0|1	65|2	4|16
Chr10	48633195	48633195	G	T	SNP	0|0	0|1	68|2	6|19
Chr10	48633563	48633563	A	C	SNP	0|1	0|1	63|11	8|18
Chr10	48635242	48635242	T	G	SNP	0|1	0|1	25|2	5|9
Chr10	48635278	48635278	C	T	SNP	0|0	0|1	39|2	5|11
Chr10	48635309	48635309	A	G	SNP	0|0	0|1	46|2	7|8
Chr10	48635357	48635357	A	T	SNP	0|0	0|1	48|2	7|5
Chr10	48639271	48639271	G	A	SNP	0|1	0|1	22|3	2|9
Chr10	48639422	48639422	A	G	SNP	0|0	0|1	22|1	4|12
Chr10	48639491	48639491	G	A	SNP	0|0	0|1	38|0	4|10
Chr10	48640916	48640916	A	T	SNP	0|1	0|1	10|1	9|11
Chr10	48645454	48645454	G	A	SNP	0|1	0|1	55|6	6|12
Chr10	48645712	48645712	A	T	SNP	0|0	0|1	55|4	8|19
Chr10	48645727	48645727	A	G	SNP	0|1	0|1	54|4	8|16
Chr10	48645738	48645738	C	T	SNP	0|1	0|1	50|4	5|16
Chr10	48648165	48648165	C	A	SNP	0|1	0|1	22|2	5|3
Chr10	48648176	48648176	G	C	SNP	0|1	0|1	22|2	5|3
Chr10	48648644	48648644	G	A	SNP	0|1	0|1	12|1	2|8
Chr10	48648675	48648675	G	T	SNP	0|1	0|1	18|3	2|9
Chr10	48649072	48649072	G	T	SNP	0|0	0|1	47|1	5|10
Chr10	48649089	48649089	T	A	SNP	0|0	0|1	44|1	6|10
Chr10	48649271	48649271	G	A	SNP	0|0	0|1	54|2	4|6
Chr10	48649395	48649395	A	G	SNP	0|1	0|1	65|9	7|17
Chr10	48649520	48649520	A	T	SNP	0|0	0|1	66|4	8|15
Chr10	48649547	48649547	C	A	SNP	0|0	0|1	69|3	7|12
Chr10	48649555	48649555	G	A	SNP	0|0	0|1	68|3	7|14
Chr10	48649689	48649689	C	T	SNP	0|1	0|1	42|4	4|15
Chr10	48649818	48649818	T	G	SNP	0|0	0|1	42|1	3|12
Chr10	48649822	48649822	G	A	SNP	0|0	0|1	42|1	3|12
Chr10	48650202	48650202	T	C	SNP	0|1	0|1	9|2	4|8
Chr10	48650428	48650428	G	T	SNP	0|0	0|1	24|0	4|4
Chr10	48650429	48650429	T	A	SNP	0|0	0|1	24|0	4|4
Chr10	48650521	48650521	G	C	SNP	0|1	0|1	35|4	5|10
Chr10	48650533	48650533	A	C	SNP	0|1	0|1	33|4	6|10

Chr10	48650663	48650663	G	T	SNP	0|0	0|1	42|2	2|16
Chr10	48651316	48651316	T	A	SNP	0|1	1|1	39|8	1|15
Chr10	48651366	48651366	G	A	SNP	0|1	1|1	57|6	1|17
Chr10	48651400	48651400	G	A	SNP	0|0	0|1	56|4	1|13
Chr10	48651711	48651711	C	T	SNP	0|0	0|1	35|1	6|19
Chr10	48652791	48652791	T	A	SNP	0|1	0|1	33|5	5|6
Chr10	48652793	48652793	T	A	SNP	0|1	0|1	34|5	7|7
Chr10	48653165	48653165	C	G	SNP	0|0	0|1	75|3	3|15
Chr10	48653465	48653465	G	C	SNP	0|0	0|1	63|1	5|21
Chr10	48655879	48655879	C	A	SNP	0|1	0|1	33|4	5|3
Chr10	48656994	48656994	A	C	SNP	0|1	0|1	57|5	4|10
Chr10	48657030	48657030	T	C	SNP	0|1	0|1	50|5	2|8
Chr10	48657366	48657366	G	A	SNP	0|1	0|1	43|4	4|9
Chr10	48657537	48657537	A	G	SNP	0|1	0|1	25|6	2|7
Chr10	48657753	48657753	T	C	SNP	0|1	0|1	19|4	6|9
Chr10	48657833	48657833	G	A	SNP	0|1	0|1	16|3	4|5
Chr10	48657880	48657880	T	C	SNP	0|1	0|1	22|3	5|9
Chr10	48658012	48658012	A	G	SNP	0|1	0|1	39|5	3|9
Chr10	48658134	48658134	A	T	SNP	0|1	0|1	23|2	4|5
Chr10	48658356	48658356	A	G	SNP	0|1	0|1	38|6	2|8
Chr10	48658416	48658416	A	G	SNP	0|1	0|1	42|7	2|6
Chr10	48658440	48658440	G	A	SNP	0|1	0|1	36|4	2|6
Chr10	48658463	48658463	G	T	SNP	0|1	0|1	36|2	3|7
Chr10	48658487	48658487	G	T	SNP	0|0	0|1	42|2	8|7
Chr10	48658502	48658502	G	T	SNP	0|0	0|1	42|2	7|6
Chr10	48658624	48658624	A	T	SNP	0|0	0|1	59|1	7|8
Chr10	48658751	48658751	A	T	SNP	0|0	0|1	28|0	7|10
Chr10	48658819	48658819	G	A	SNP	0|0	0|1	44|2	5|6
Chr10	48658941	48658941	C	T	SNP	0|0	0|1	51|0	5|13
Chr10	48658942	48658942	C	T	SNP	0|0	0|1	51|0	5|13
Chr10	48659145	48659145	T	A	SNP	0|1	0|1	52|7	4|17
Chr10	48659278	48659278	C	G	SNP	0|1	0|1	42|4	7|13
Chr10	48660182	48660182	T	C	SNP	0|0	0|1	30|1	4|4
Chr10	48660218	48660218	G	C	SNP	0|0	0|1	25|1	6|4
Chr10	48660578	48660578	C	A	SNP	0|0	0|1	78|3	5|13
Chr10	48661165	48661165	G	A	SNP	0|1	0|1	34|3	1|10
Chr10	48661174	48661174	T	G	SNP	0|1	0|1	33|5	1|8
Chr10	48661495	48661495	A	G	SNP	0|1	0|1	9|7	3|5
Chr10	48661502	48661502	T	G	SNP	0|1	0|1	9|7	3|5
Chr10	48661551	48661551	C	T	SNP	0|1	0|1	12|5	3|8
Chr10	48661757	48661757	C	T	SNP	0|1	0|1	29|3	3|6
Chr10	48662263	48662263	T	A	SNP	0|0	0|1	29|2	3|8

Chr10	48662349	48662349	C	G	SNP	0|0	0|1	31|2	2|14
Chr10	48663163	48663163	T	G	SNP	0|0	0|1	13|0	3|9
Chr10	48663958	48663958	G	A	SNP	0|0	0|1	34|2	2|7
Chr10	48664241	48664241	A	G	SNP	0|0	0|1	13|0	5|4
Chr10	48664990	48664990	C	T	SNP	0|1	0|1	18|3	5|8
Chr10	48665232	48665232	C	T	SNP	0|0	0|1	18|0	1|7
Chr10	48665361	48665361	G	T	SNP	0|0	0|1	28|2	3|11
Chr10	48665671	48665671	C	A	SNP	0|0	0|1	48|2	8|19
Chr10	48665705	48665705	A	C	SNP	0|0	0|1	42|0	5|23
Chr10	48665740	48665740	T	C	SNP	0|0	0|1	51|0	7|22
Chr10	48666574	48666574	C	T	SNP	0|0	0|1	36|3	4|5
Chr10	48668636	48668636	T	G	SNP	0|1	0|1	65|9	4|14
Chr10	48668703	48668703	A	G	SNP	0|1	0|1	60|8	12|18
Chr10	48669722	48669722	G	A	SNP	0|0	0|1	44|2	2|10
Chr10	48669723	48669723	T	A	SNP	0|0	0|1	44|2	2|10
Chr10	48670365	48670365	T	G	SNP	0|0	0|1	22|1	4|11
Chr10	48670376	48670376	G	A	SNP	0|0	0|1	25|1	5|13
Chr10	48670430	48670430	A	C	SNP	0|0	0|1	28|1	3|18
Chr10	48670447	48670447	A	G	SNP	0|0	0|1	31|1	3|17
Chr10	48670558	48670558	A	G	SNP	0|0	0|1	43|2	7|16
Chr10	48670629	48670629	C	T	SNP	0|0	0|1	35|2	12|12
Chr10	48670631	48670631	G	T	SNP	0|0	0|1	35|2	12|12
Chr10	48670634	48670634	G	T	SNP	0|0	0|1	35|2	12|11
Chr10	48670848	48670848	G	A	SNP	0|0	0|1	37|2	5|13
Chr10	48670868	48670868	G	A	SNP	0|0	0|1	36|2	3|10
Chr10	48670942	48670942	G	T	SNP	0|0	0|1	34|2	4|8
Chr10	48671006	48671006	T	A	SNP	0|0	1|1	34|3	1|15
Chr10	48671205	48671205	A	C	SNP	0|1	0|1	37|5	2|6
Chr10	48671231	48671231	A	G	SNP	0|1	0|1	35|5	3|5
Chr10	48671790	48671790	A	C	SNP	0|0	0|1	21|0	4|4
Chr10	48672234	48672234	T	C	SNP	0|0	0|1	26|0	1|7
Chr10	48672249	48672249	T	C	SNP	0|0	0|1	27|0	1|7
Chr10	48672295	48672295	T	C	SNP	0|0	0|1	26|1	3|8
Chr10	48672306	48672306	A	C	SNP	0|1	0|1	27|2	3|9
Chr10	48672317	48672317	T	C	SNP	0|1	0|1	27|2	4|10
Chr10	48672322	48672322	C	T	SNP	0|1	0|1	27|2	4|10
Chr10	48672328	48672328	T	C	SNP	0|0	0|1	29|2	4|10
Chr10	48672332	48672332	G	C	SNP	0|0	0|1	28|2	4|13
Chr10	48672339	48672339	C	T	SNP	0|0	0|1	32|2	4|15
Chr10	48672348	48672348	T	C	SNP	0|0	0|1	36|2	4|16
Chr10	48672362	48672362	C	G	SNP	0|0	0|1	43|2	4|15
Chr10	48672385	48672385	T	C	SNP	0|0	0|1	48|2	4|14

Chr10	48672391	48672391	C	T	SNP	0|0	0|1	49|2	4|14
Chr10	48672434	48672434	A	G	SNP	0|0	0|1	64|2	6|15
Chr10	48672557	48672557	A	G	SNP	0|1	0|1	68|5	4|19
Chr10	48672566	48672566	G	A	SNP	0|1	0|1	68|5	4|20
Chr10	48672830	48672830	T	C	SNP	0|0	0|1	49|1	10|9
Chr10	48672879	48672879	G	T	SNP	0|0	0|1	38|1	10|12
Chr10	48673018	48673018	T	G	SNP	0|0	0|1	44|2	6|3
Chr10	48676211	48676211	G	A	SNP	0|0	0|1	39|1	7|3
Chr10	48676219	48676219	G	A	SNP	0|0	0|1	43|1	7|3
Chr10	48676223	48676223	T	C	SNP	0|0	0|1	43|0	7|3
Chr10	48676229	48676229	C	T	SNP	0|0	0|1	43|0	7|3
Chr10	48676237	48676237	G	A	SNP	0|0	0|1	44|0	5|3
Chr10	48676247	48676247	T	C	SNP	0|0	0|1	36|0	5|3
Chr10	48676253	48676253	G	A	SNP	0|0	0|1	35|0	6|3
Chr10	48676257	48676257	G	A	SNP	0|0	0|1	36|0	5|3
Chr10	48676262	48676262	G	A	SNP	0|0	0|1	38|0	5|3
Chr10	48676280	48676280	G	A	SNP	0|0	0|1	39|0	5|3
Chr10	48676284	48676284	C	T	SNP	0|0	0|1	39|0	5|3
Chr10	48676442	48676442	T	C	SNP	0|0	0|1	23|0	4|7
Chr10	48676513	48676513	A	C	SNP	0|1	0|1	22|2	8|5
Chr10	48676549	48676549	C	A	SNP	0|1	0|1	21|2	7|4
Chr10	48680571	48680571	T	G	SNP	0|0	0|1	55|3	8|12
Chr10	48680577	48680577	A	C	SNP	0|0	0|1	62|3	8|13
Chr10	48680679	48680679	T	G	SNP	0|0	0|1	52|3	2|13
Chr10	48680764	48680764	A	G	SNP	0|0	0|1	55|2	2|17
Chr10	48680805	48680805	T	G	SNP	0|1	0|1	54|4	4|17
Chr10	48680937	48680937	A	C	SNP	0|1	0|1	55|4	5|17
Chr10	48680959	48680959	T	C	SNP	0|1	0|1	54|4	3|16
Chr10	48681280	48681280	C	T	SNP	0|0	0|1	61|1	12|16
Chr10	48681319	48681319	G	C	SNP	0|0	0|1	63|2	11|19
Chr10	48681531	48681531	A	T	SNP	0|0	0|1	60|4	9|13
Chr10	48682082	48682082	A	G	SNP	0|1	0|1	46|4	3|8
Chr10	48682162	48682162	C	T	SNP	0|0	0|1	60|4	4|6
Chr10	48682163	48682163	T	C	SNP	0|0	0|1	59|4	4|6
Chr10	48682401	48682401	C	T	SNP	0|0	0|1	76|2	12|19
Chr10	48682461	48682461	A	C	SNP	0|0	0|1	77|2	11|15
Chr10	48682619	48682619	C	A	SNP	0|1	1|1	64|7	1|21
Chr10	48683420	48683420	G	T	SNP	0|0	0|1	13|0	4|5
Chr10	48683577	48683577	A	G	SNP	0|1	0|1	37|3	2|6
Chr10	48684185	48684185	G	C	SNP	0|0	0|1	58|2	9|6
Chr10	48684310	48684310	A	T	SNP	0|0	0|1	55|3	9|12
Chr10	48684351	48684351	C	G	SNP	0|0	0|1	48|3	9|8

Chr10	48684472	48684472	C	G	SNP	0|0	0|1	49|1	6|8
Chr10	48684756	48684756	A	G	SNP	0|0	0|1	35|0	3|12
Chr10	48684997	48684997	G	T	SNP	0|1	0|1	40|5	1|12
Chr10	48685085	48685085	T	C	SNP	0|1	0|1	32|6	1|11
Chr10	48685101	48685101	C	A	SNP	0|1	0|1	34|6	1|13
Chr10	48685183	48685183	A	T	SNP	0|0	1|1	37|2	0|14
Chr10	48685218	48685218	G	A	SNP	0|0	1|1	43|0	0|10
Chr10	48685597	48685597	T	A	SNP	0|0	0|1	53|3	6|16
Chr10	48685703	48685703	T	C	SNP	0|1	0|1	37|5	4|16
Chr10	48686293	48686293	G	A	SNP	0|0	0|1	42|3	5|12
Chr10	48686741	48686741	T	A	SNP	0|1	1|1	22|2	0|9
Chr10	48686750	48686750	A	T	SNP	0|1	0|1	20|2	1|9
Chr10	48687027	48687027	C	T	SNP	0|0	0|1	25|0	2|14
Chr10	48687947	48687947	C	T	SNP	0|0	0|1	26|1	3|7
Chr10	48688223	48688223	A	G	SNP	0|0	0|1	39|0	4|7
Chr10	48688252	48688252	T	C	SNP	0|0	0|1	39|0	6|8
Chr10	48688583	48688583	A	G	SNP	0|1	0|1	47|6	4|17
Chr10	48689540	48689540	C	T	SNP	0|0	0|1	65|0	10|13
Chr10	48690060	48690060	C	G	SNP	0|0	0|1	59|4	11|9
Chr10	48690259	48690259	A	G	SNP	0|1	0|1	52|4	8|17
Chr10	48691897	48691897	T	C	SNP	0|0	0|1	30|2	3|7
Chr10	48692446	48692446	C	T	SNP	0|1	0|1	40|9	6|14
Chr10	48692693	48692693	A	T	SNP	0|1	0|1	45|4	5|12
Chr10	48692695	48692695	G	T	SNP	0|1	0|1	45|4	5|12
Chr10	48692988	48692988	G	T	SNP	0|0	0|1	45|3	9|15
Chr10	48694224	48694224	G	T	SNP	0|1	0|1	16|2	4|4
Chr10	48694502	48694502	T	A	SNP	0|1	0|1	10|1	5|4
Chr10	48694587	48694587	G	A	SNP	0|1	0|1	11|2	7|7
Chr10	48694715	48694715	C	T	SNP	0|1	0|1	17|2	5|9
Chr10	48694802	48694802	G	A	SNP	0|1	0|1	19|6	5|20
Chr10	48694848	48694848	A	G	SNP	0|1	0|1	23|5	3|16
Chr10	48694889	48694889	A	G	SNP	0|1	1|1	31|4	2|15
Chr10	48694905	48694905	T	C	SNP	0|1	0|1	32|4	2|10
Chr10	48694957	48694957	C	T	SNP	0|0	0|1	22|0	4|6
Chr10	48695063	48695063	A	G	SNP	0|1	0|1	14|3	2|6
Chr10	48695541	48695541	T	A	SNP	0|1	0|1	10|2	2|6
Chr10	48695781	48695781	A	G	SNP	0|0	0|1	46|3	5|19
Chr10	48695811	48695811	T	G	SNP	0|0	0|1	50|2	3|18
Chr10	48696487	48696487	G	A	SNP	0|0	0|1	22|1	3|12
Chr10	48697429	48697429	C	T	SNP	0|1	0|1	66|5	8|8
Chr10	48697764	48697764	T	G	SNP	0|1	0|1	55|5	12|13
Chr10	48698344	48698344	G	A	SNP	0|1	0|1	37|4	5|9

Chr10	48699467	48699467	A	C	SNP	0|0	0|1	51|1	5|14
Chr10	48700169	48700169	A	C	SNP	0|0	0|1	33|3	11|16
Chr10	48700578	48700578	G	A	SNP	0|1	0|1	36|8	8|8
Chr10	48702429	48702429	A	T	SNP	0|1	0|1	25|4	5|7
Chr10	48702430	48702430	A	T	SNP	0|1	0|1	26|4	5|7
Chr10	48702431	48702431	A	T	SNP	0|1	0|1	26|4	5|7
Chr10	48702811	48702811	C	T	SNP	0|0	0|1	45|1	11|13
Chr10	48703926	48703926	A	T	SNP	0|0	0|1	22|0	2|10
Chr10	48705545	48705545	C	A	SNP	0|1	0|1	38|4	3|8
Chr10	48705662	48705662	G	A	SNP	0|0	0|1	49|1	2|13
Chr10	48705932	48705932	G	A	SNP	0|1	0|1	44|11	8|7
Chr10	48706903	48706903	T	C	SNP	0|1	0|1	44|4	5|12
Chr10	48707094	48707094	A	G	SNP	0|0	0|1	31|0	4|13
Chr10	48707192	48707192	G	C	SNP	0|0	0|1	34|0	3|12
Chr10	48707615	48707615	T	C	SNP	0|0	0|1	45|1	7|5
Chr10	48713269	48713269	G	A	SNP	0|1	0|1	27|5	3|6
Chr10	48717351	48717351	C	T	SNP	0|1	0|1	30|3	6|13
Chr10	48719373	48719373	C	T	SNP	0|1	0|1	41|8	5|11
Chr10	48719400	48719400	A	C	SNP	0|1	0|1	42|8	3|12
Chr10	48719511	48719511	C	T	SNP	0|1	0|1	25|6	3|16
Chr10	48719570	48719570	G	C	SNP	0|0	0|1	40|3	2|19
Chr10	48719600	48719600	T	A	SNP	0|0	0|1	49|4	2|18
Chr10	48719678	48719678	C	T	SNP	0|1	0|1	49|4	2|15
Chr10	48719679	48719679	T	A	SNP	0|1	0|1	48|4	2|15
Chr10	48720678	48720678	G	A	SNP	0|1	0|1	30|4	6|4
Chr10	48722910	48722910	A	G	SNP	0|0	0|1	24|1	2|12
Chr10	48722945	48722945	A	G	SNP	0|1	0|1	28|3	2|12
Chr10	48723132	48723132	A	G	SNP	0|0	0|1	34|2	5|9
Chr10	48723250	48723250	C	A	SNP	0|0	0|1	21|1	4|5
Chr10	48723251	48723251	T	A	SNP	0|0	0|1	21|1	4|5
Chr10	48723473	48723473	T	A	SNP	0|0	0|1	15|0	4|5
Chr10	48724870	48724870	G	C	SNP	0|1	0|1	21|11	1|10
Chr10	48727894	48727894	A	T	SNP	0|0	0|1	44|5	3|13
Chr10	48731730	48731730	T	C	SNP	0|0	0|1	74|5	6|22
Chr10	48732383	48732383	G	A	SNP	0|1	0|1	70|4	9|7
Chr10	48732397	48732397	C	T	SNP	0|0	0|1	71|5	9|8
Chr10	48732689	48732689	C	G	SNP	0|0	0|1	36|1	2|7
Chr10	48733167	48733167	A	T	SNP	0|0	0|1	13|0	2|8
Chr10	48734272	48734272	G	T	SNP	0|1	0|1	9|4	2|7
Chr10	48735482	48735482	T	A	SNP	0|0	0|1	48|5	3|6
Chr10	48735532	48735532	G	C	SNP	0|0	0|1	39|2	3|7
Chr10	48736222	48736222	G	A	SNP	0|0	0|1	56|2	4|14

Chr10	48736355	48736355	A	T	SNP	0|1	0|1	61|9	3|15
Chr10	48736839	48736839	A	C	SNP	0|1	0|1	51|7	1|19
Chr10	48737635	48737635	A	T	SNP	0|0	0|1	59|3	7|9
Chr10	48740699	48740699	C	G	SNP	0|1	1|1	14|2	0|9
Chr10	48740756	48740756	G	T	SNP	0|1	0|1	19|2	2|13
Chr10	48741105	48741105	T	C	SNP	0|0	0|1	72|4	5|14
Chr10	48741133	48741133	T	C	SNP	0|0	0|1	60|4	6|15
Chr10	48741165	48741165	A	G	SNP	0|1	0|1	52|9	7|15
Chr10	48741461	48741461	G	A	SNP	0|1	0|1	45|5	5|11
Chr10	48742045	48742045	A	T	SNP	0|1	0|1	52|7	5|20
Chr10	48742305	48742305	T	C	SNP	0|1	0|1	50|6	9|10
Chr10	48742601	48742601	C	T	SNP	0|1	0|1	49|7	7|15
Chr10	48742642	48742642	T	A	SNP	0|1	0|1	53|5	6|15
Chr10	48743263	48743263	C	G	SNP	0|0	0|1	51|4	7|12
Chr10	48743324	48743324	T	G	SNP	0|1	0|1	52|4	5|11
Chr10	48743372	48743372	G	A	SNP	0|1	0|1	46|7	4|14
Chr10	48743475	48743475	C	T	SNP	0|1	0|1	42|6	10|11
Chr10	48743477	48743477	C	A	SNP	0|1	0|1	41|6	10|11
Chr10	48743762	48743762	T	C	SNP	0|1	0|1	38|4	4|7
Chr10	48743763	48743763	G	A	SNP	0|1	0|1	38|4	4|7
Chr10	48743852	48743852	A	C	SNP	0|1	0|1	44|4	6|9
Chr10	48743889	48743889	C	T	SNP	0|1	0|1	42|4	8|10
Chr10	48744035	48744035	C	G	SNP	0|1	0|1	64|5	11|23
Chr10	48744092	48744092	T	C	SNP	0|1	0|1	71|9	10|19
Chr10	48744276	48744276	G	A	SNP	0|1	0|1	63|8	6|19
Chr10	48744347	48744347	T	C	SNP	0|0	0|1	82|7	5|25
Chr10	48744423	48744423	A	C	SNP	0|0	0|1	71|7	9|20
Chr10	48744821	48744821	C	G	SNP	0|1	0|1	63|11	8|12
Chr10	48744940	48744940	T	C	SNP	0|1	0|1	57|7	5|18
Chr10	48745163	48745163	C	T	SNP	0|1	0|1	56|6	8|17
Chr10	48745226	48745226	C	A	SNP	0|1	1|1	37|5	1|16
Chr10	48745341	48745341	A	C	SNP	0|0	0|1	46|2	2|8
Chr10	48745741	48745741	G	A	SNP	0|1	0|1	35|4	11|20
Chr10	48745820	48745820	G	A	SNP	0|0	0|1	43|3	11|19
Chr10	48745960	48745960	G	A	SNP	0|0	0|1	34|2	5|12
Chr10	48746103	48746103	T	C	SNP	0|0	0|1	43|1	6|11
Chr10	48746110	48746110	C	T	SNP	0|0	0|1	44|1	6|12
Chr10	48746261	48746261	T	C	SNP	0|0	0|1	62|0	7|17
Chr10	48746431	48746431	C	G	SNP	0|1	0|1	35|3	1|13
Chr10	48746778	48746778	G	A	SNP	0|1	0|1	36|4	6|6
Chr10	48747425	48747425	G	A	SNP	0|0	0|1	11|0	3|7
Chr10	48747431	48747431	G	C	SNP	0|0	0|1	11|0	3|5

Chr10	48749500	48749500	G	T	SNP	0|1	0|1	36|3	3|12
Chr10	48752722	48752722	T	C	SNP	0|0	0|1	48|3	8|20
Chr10	48752737	48752737	A	G	SNP	0|0	0|1	47|3	9|20
Chr10	48753597	48753597	A	G	SNP	0|1	0|1	51|5	9|11
Chr10	48753641	48753641	A	G	SNP	0|0	0|1	45|3	9|10
Chr10	48754709	48754709	T	C	SNP	0|1	0|1	55|6	11|8
Chr10	48755332	48755332	A	G	SNP	0|1	0|1	53|6	6|16
Chr10	48755373	48755373	C	T	SNP	0|1	0|1	42|6	6|16
Chr10	48755753	48755753	G	C	SNP	0|0	0|1	53|2	9|19
Chr10	48756117	48756117	G	T	SNP	0|0	0|1	37|0	5|13
Chr10	48756143	48756143	C	T	SNP	0|0	0|1	33|0	6|16
Chr10	48757048	48757048	T	A	SNP	0|0	0|1	41|3	4|10
Chr10	48757086	48757086	G	T	SNP	0|0	0|1	48|3	8|8
Chr10	48757207	48757207	A	T	SNP	0|1	0|1	39|3	4|4
Chr10	48757726	48757726	G	A	SNP	0|0	0|1	42|2	6|3
Chr10	48757755	48757755	A	C	SNP	0|0	0|1	35|2	6|6
Chr10	48758197	48758197	G	C	SNP	0|0	0|1	54|4	4|18
Chr10	48758267	48758267	T	A	SNP	0|0	0|1	70|4	3|22
Chr10	48758917	48758917	T	C	SNP	0|0	0|1	15|2	1|7
Chr10	48759250	48759250	C	T	SNP	0|0	0|1	19|0	6|5
Chr10	48759541	48759541	A	C	SNP	0|1	1|1	35|5	0|17
Chr10	48760427	48760427	C	T	SNP	0|1	0|1	65|7	11|9
Chr10	48760871	48760871	A	T	SNP	0|1	0|1	19|8	8|8
Chr10	48762117	48762117	C	G	SNP	0|1	0|1	9|2	1|8
Chr10	48763587	48763587	G	A	SNP	0|0	0|1	36|1	3|17
Chr10	48765186	48765186	A	T	SNP	0|0	0|1	44|2	3|8
Chr10	48765248	48765248	C	A	SNP	0|0	0|1	52|3	2|7
Chr10	48765295	48765295	T	A	SNP	0|0	0|1	50|4	2|7
Chr10	48765351	48765351	A	T	SNP	0|1	0|1	46|6	6|8
Chr10	48765501	48765501	T	C	SNP	0|0	0|1	37|2	6|13
Chr10	48765594	48765594	T	C	SNP	0|1	0|1	41|3	3|8
Chr10	48765710	48765710	T	C	SNP	0|1	0|1	26|4	2|7
Chr10	48765809	48765809	A	G	SNP	0|1	0|1	10|2	1|7
Chr10	48766703	48766703	C	T	SNP	0|0	0|1	64|3	1|9
Chr10	48767026	48767026	A	T	SNP	0|0	0|1	59|3	3|11
Chr10	48767062	48767062	T	C	SNP	0|0	0|1	53|3	2|10
Chr10	48767471	48767471	G	T	SNP	0|1	0|1	23|9	5|4
Chr10	48767538	48767538	A	T	SNP	0|1	0|1	28|6	5|4
Chr10	48767778	48767778	T	C	SNP	0|1	0|1	42|5	3|8
Chr10	48767823	48767823	G	A	SNP	0|1	0|1	49|4	1|13
Chr10	48769223	48769223	A	G	SNP	0|0	0|1	43|2	1|10
Chr10	48769652	48769652	A	G	SNP	0|1	0|1	59|5	7|18

Chr10	48769762	48769762	A	G	SNP	0|1	0|1	45|5	7|20
Chr10	48770139	48770139	A	T	SNP	0|1	0|1	53|6	9|7
Chr10	48770667	48770667	G	T	SNP	0|1	0|1	69|6	8|10
Chr10	48770957	48770957	A	G	SNP	0|1	0|1	12|1	3|6
Chr10	48772965	48772965	C	G	SNP	0|1	0|1	49|10	6|16
Chr10	48773810	48773810	G	C	SNP	0|1	0|1	65|9	10|15
Chr10	48773848	48773848	A	G	SNP	0|1	0|1	64|9	11|13
Chr10	48773851	48773851	C	T	SNP	0|1	0|1	64|9	11|12
Chr10	48774117	48774117	T	A	SNP	0|1	0|1	53|5	5|9
Chr10	48774424	48774424	C	G	SNP	0|1	0|1	54|7	6|15
Chr10	48774649	48774649	G	A	SNP	0|0	0|1	54|3	4|19
Chr10	48774928	48774928	T	A	SNP	0|1	0|1	35|5	9|10
Chr10	48775249	48775249	G	C	SNP	0|1	0|1	7|4	4|7
Chr10	48775608	48775608	C	T	SNP	0|0	0|1	42|2	8|7
Chr10	48775610	48775610	T	C	SNP	0|0	0|1	44|2	8|7
Chr10	48775836	48775836	T	C	SNP	0|0	0|1	43|2	4|8
Chr10	48775966	48775966	C	T	SNP	0|1	0|1	65|6	7|20
Chr10	48776182	48776182	G	A	SNP	0|0	0|1	91|4	7|16
Chr10	48777960	48777960	A	G	SNP	0|1	0|1	46|9	1|10
Chr10	48778983	48778983	G	T	SNP	0|0	0|1	58|2	7|13
Chr10	48779164	48779164	G	A	SNP	0|0	0|1	74|4	4|10
Chr10	48779404	48779404	A	G	SNP	0|0	0|1	42|2	1|9
Chr10	48780093	48780093	T	A	SNP	0|1	0|1	43|8	3|7
Chr10	48780510	48780510	G	T	SNP	0|0	0|1	53|4	4|7
Chr10	48780965	48780965	C	T	SNP	0|1	0|1	23|4	2|7
Chr10	48781151	48781151	A	G	SNP	0|0	0|1	18|1	6|6
Chr10	48781522	48781522	G	A	SNP	0|1	0|1	33|6	4|9
Chr10	48782153	48782153	A	G	SNP	0|1	0|1	28|7	5|11
Chr10	48782300	48782300	A	T	SNP	0|0	0|1	48|2	3|12
Chr10	48783554	48783554	A	G	SNP	0|1	0|1	47|10	2|12
Chr10	48783919	48783919	A	T	SNP	0|0	0|1	34|2	6|9
Chr10	48785134	48785134	G	T	SNP	0|1	0|1	35|4	4|7
Chr10	48785976	48785976	G	C	SNP	0|0	0|1	47|1	7|15
Chr10	48786364	48786364	T	C	SNP	0|1	0|1	41|7	6|7
Chr10	48786640	48786640	G	A	SNP	0|0	0|1	57|3	3|9
Chr10	48787020	48787020	A	T	SNP	0|1	0|1	47|7	5|17
Chr10	48787115	48787115	A	G	SNP	0|0	0|1	35|1	5|20
Chr10	48787790	48787790	T	A	SNP	0|0	0|1	28|1	3|11
Chr10	48787850	48787850	C	A	SNP	0|0	0|1	33|1	5|4
Chr10	48791039	48791039	A	T	SNP	0|1	0|1	46|4	6|21
Chr10	48791452	48791452	G	T	SNP	0|1	0|1	36|4	2|14
Chr10	48791621	48791621	A	C	SNP	0|1	0|1	26|3	1|8

Chr10	48791629	48791629	A	G	SNP	0|1	0|1	23|3	1|8
Chr10	48791692	48791692	A	G	SNP	0|1	0|1	14|3	2|8
Chr10	48791715	48791715	G	A	SNP	0|1	0|1	16|2	2|8
Chr10	48791950	48791950	A	C	SNP	0|1	0|1	18|2	3|7
Chr10	48792611	48792611	C	T	SNP	0|1	0|1	20|3	1|7
Chr10	48792612	48792612	A	G	SNP	0|1	0|1	20|3	1|7
Chr10	48792629	48792629	G	A	SNP	0|1	0|1	21|3	1|7
Chr10	48792648	48792648	T	C	SNP	0|1	0|1	23|2	3|7
Chr10	48792826	48792826	C	T	SNP	0|0	0|1	25|2	2|6
Chr10	48792847	48792847	T	A	SNP	0|0	0|1	21|0	2|8
Chr10	48793396	48793396	A	T	SNP	0|1	0|1	11|2	2|9
Chr10	48796665	48796665	A	C	SNP	0|1	0|1	20|2	5|5
Chr10	48796735	48796735	G	A	SNP	0|1	0|1	25|1	3|7
Chr10	48796736	48796736	G	A	SNP	0|1	0|1	25|1	3|7
Chr10	48796749	48796749	G	A	SNP	0|1	0|1	24|2	4|6
Chr10	48796750	48796750	T	C	SNP	0|1	0|1	24|2	4|6
Chr10	48797051	48797051	A	G	SNP	0|0	0|1	44|3	5|7
Chr10	48797074	48797074	C	T	SNP	0|1	0|1	51|5	5|7
Chr10	48797154	48797154	C	T	SNP	0|0	0|1	48|3	4|5
Chr10	48797155	48797155	C	T	SNP	0|0	0|1	48|3	4|5
Chr10	48797200	48797200	C	T	SNP	0|1	0|1	46|5	2|7
Chr10	48798373	48798373	G	A	SNP	0|1	1|1	24|6	0|9
Chr10	48798595	48798595	A	G	SNP	0|1	0|1	31|4	2|9
Chr10	48798608	48798608	G	A	SNP	0|1	0|1	31|4	2|9
Chr10	48798615	48798615	A	G	SNP	0|1	0|1	31|3	1|9
Chr10	48798780	48798780	A	C	SNP	0|1	0|1	36|7	2|6
Chr10	48798945	48798945	C	T	SNP	0|0	0|1	41|1	4|11
Chr10	48799491	48799491	G	T	SNP	0|0	0|1	31|2	5|14
Chr10	48800817	48800817	C	T	SNP	0|1	0|1	25|4	5|14
Chr10	48801452	48801452	G	C	SNP	0|0	0|1	40|2	3|11
Chr10	48801728	48801728	G	A	SNP	0|1	0|1	42|7	1|10
Chr10	48803043	48803043	C	T	SNP	0|0	0|1	15|1	5|3
Chr10	48804531	48804531	T	A	SNP	0|0	0|1	15|1	2|11
Chr10	48804539	48804539	G	A	SNP	0|0	0|1	16|1	2|14
Chr10	48804561	48804561	G	A	SNP	0|1	1|1	16|3	0|14
Chr10	48806258	48806258	G	A	SNP	0|1	0|1	33|10	3|8
Chr10	48807379	48807379	C	T	SNP	0|0	0|1	63|5	9|18
Chr10	48807977	48807977	A	G	SNP	0|1	0|1	63|8	10|12
Chr10	48808277	48808277	A	G	SNP	0|1	0|1	41|6	5|12
Chr10	48808663	48808663	T	C	SNP	0|0	0|1	37|1	4|7
Chr10	48809105	48809105	T	A	SNP	0|0	0|1	18|1	8|4
Chr10	48810763	48810763	G	T	SNP	0|0	0|1	28|1	2|7

Chr10	48810815	48810815	T	C	SNP	0|0	0|1	33|0	2|6
Chr10	48811724	48811724	C	T	SNP	0|0	0|1	26|2	3|5
Chr10	48813449	48813449	T	A	SNP	0|0	0|1	20|1	2|9
Chr10	48813570	48813570	A	T	SNP	0|1	0|1	29|7	4|7
Chr10	48813696	48813696	C	A	SNP	0|1	0|1	28|5	7|9
Chr10	48813721	48813721	G	A	SNP	0|1	0|1	32|4	11|13
Chr10	48813736	48813736	C	A	SNP	0|1	0|1	33|4	13|10
Chr10	48813765	48813765	C	T	SNP	0|1	0|1	38|3	11|11
Chr10	48813792	48813792	A	G	SNP	0|0	0|1	44|2	11|16
Chr10	48813835	48813835	T	C	SNP	0|0	0|1	55|2	13|17
Chr10	48813955	48813955	T	C	SNP	0|0	0|1	59|3	7|21
Chr10	48813961	48813961	C	A	SNP	0|0	0|1	58|3	7|22
Chr10	48814112	48814112	G	A	SNP	0|1	0|1	51|7	15|17
Chr10	48814166	48814166	G	A	SNP	0|0	0|1	52|5	14|25
Chr10	48814205	48814205	G	A	SNP	0|1	0|1	59|5	14|22
Chr10	48814206	48814206	T	C	SNP	0|1	0|1	59|5	14|23
Chr10	48814217	48814217	T	G	SNP	0|1	0|1	57|5	15|24
Chr10	48814226	48814226	C	T	SNP	0|1	0|1	54|5	15|24
Chr10	48814322	48814322	G	T	SNP	0|0	0|1	44|0	6|12
Chr10	48814381	48814381	G	T	SNP	0|0	0|1	32|0	7|9
Chr10	48815818	48815818	T	A	SNP	0|1	0|1	24|3	2|12
Chr10	48816916	48816916	C	A	SNP	0|0	0|1	11|0	2|8
Chr10	48817603	48817603	A	C	SNP	0|0	0|1	48|4	6|14
Chr10	48818217	48818217	A	G	SNP	0|1	0|1	48|7	8|25
Chr10	48818911	48818911	A	G	SNP	0|1	0|1	57|5	5|22
Chr10	48820456	48820456	C	T	SNP	0|1	0|1	49|6	6|18
Chr10	48820981	48820981	A	T	SNP	0|1	0|1	41|6	3|13
Chr10	48821422	48821422	C	A	SNP	0|1	0|1	44|7	5|30
Chr10	48824175	48824175	A	T	SNP	0|1	0|1	16|4	6|21
Chr10	48824288	48824288	C	T	SNP	0|1	0|1	26|4	7|10
Chr10	48824355	48824355	T	C	SNP	0|1	0|1	22|4	5|10
Chr10	48826295	48826295	A	T	SNP	0|1	0|1	32|5	4|11
Chr10	48826415	48826415	G	A	SNP	0|1	0|1	41|4	5|20
Chr10	48827896	48827896	T	C	SNP	0|1	0|1	54|6	3|13
Chr10	48828257	48828257	G	A	SNP	0|1	0|1	27|4	3|11
Chr10	48828322	48828322	A	G	SNP	0|1	0|1	28|5	5|13
Chr10	48830324	48830324	T	G	SNP	0|1	0|1	21|3	4|5
Chr10	48830383	48830383	G	A	SNP	0|0	0|1	18|1	6|4
Chr10	48830749	48830749	G	A	SNP	0|0	0|1	42|2	9|14
Chr10	48830929	48830929	A	C	SNP	0|0	0|1	41|2	13|10
Chr10	48831106	48831106	T	G	SNP	0|1	0|1	32|3	3|8
Chr10	48831612	48831612	T	G	SNP	0|0	0|1	62|1	4|12

Chr10	48831689	48831689	C	T	SNP	0|0	0|1	52|2	5|10
Chr10	48832095	48832095	A	C	SNP	0|1	0|1	21|2	2|9
Chr10	48832141	48832141	C	T	SNP	0|1	0|1	21|2	4|17
Chr10	48832317	48832317	T	A	SNP	0|1	1|1	29|4	0|15
Chr10	48832461	48832461	C	G	SNP	0|1	0|1	33|3	1|9
Chr10	48833365	48833365	A	G	SNP	0|0	0|1	19|0	5|6
Chr10	48835208	48835208	G	A	SNP	0|0	0|1	22|1	2|7
Chr10	48837629	48837629	A	C	SNP	0|1	0|1	31|3	5|8
Chr10	48838167	48838167	A	T	SNP	0|1	0|1	33|3	3|7
Chr10	48838535	48838535	C	A	SNP	0|1	0|1	43|4	4|19
Chr10	48839412	48839412	A	G	SNP	0|1	0|1	9|1	3|8
Chr10	48839434	48839434	A	G	SNP	0|1	0|1	7|1	4|11
Chr10	48839452	48839452	T	C	SNP	0|1	0|1	8|1	4|12
Chr10	48839456	48839456	G	A	SNP	0|1	0|1	8|1	4|13
Chr10	48839465	48839465	T	C	SNP	0|1	0|1	8|1	2|13
Chr10	48839523	48839523	G	A	SNP	0|0	0|1	8|0	4|6
Chr10	48839536	48839536	T	C	SNP	0|0	0|1	9|0	6|4
Chr10	48839609	48839609	A	G	SNP	0|0	0|1	9|0	4|4
Chr10	48839839	48839839	C	T	SNP	0|1	0|1	8|1	3|5
Chr10	48839840	48839840	A	G	SNP	0|1	0|1	8|1	3|5
Chr10	48840431	48840431	G	A	SNP	0|1	0|1	21|2	3|8
Chr10	48840956	48840956	T	C	SNP	0|0	1|1	12|0	0|8
Chr10	48841042	48841042	T	C	SNP	0|1	0|1	22|3	1|7
Chr10	48841050	48841050	C	T	SNP	0|1	0|1	23|3	1|7
Chr10	48841582	48841582	T	C	SNP	0|1	0|1	25|2	2|6
Chr10	48841858	48841858	G	C	SNP	0|0	0|1	33|1	3|10
Chr10	48841993	48841993	A	T	SNP	0|0	0|1	32|2	3|12
Chr10	48842046	48842046	T	A	SNP	0|1	0|1	29|6	5|13
Chr10	48842077	48842077	G	C	SNP	0|1	0|1	34|6	5|11
Chr10	48842694	48842694	G	A	SNP	0|1	0|1	24|3	6|8
Chr10	48842718	48842718	G	A	SNP	0|1	0|1	32|3	6|8
Chr10	48842771	48842771	T	C	SNP	0|0	0|1	44|3	6|7
Chr10	48842816	48842816	A	G	SNP	0|1	0|1	39|3	3|5
Chr10	48842909	48842909	A	G	SNP	0|1	0|1	34|6	3|8
Chr10	48842935	48842935	A	G	SNP	0|1	0|1	35|6	3|5
Chr10	48842951	48842951	C	G	SNP	0|1	0|1	41|6	4|4
Chr10	48842975	48842975	A	G	SNP	0|1	0|1	43|5	4|5
Chr10	48844921	48844921	C	G	SNP	0|1	1|1	20|2	0|8
Chr10	48846020	48846020	C	T	SNP	0|1	0|1	54|4	10|11
Chr10	48847388	48847388	C	G	SNP	0|0	0|1	32|2	11|11
Chr10	48847881	48847881	C	T	SNP	0|0	0|1	34|1	4|7
Chr10	48848361	48848361	A	T	SNP	0|1	0|1	29|3	2|10

Chr10	48848514	48848514	C	A	SNP	0|0	0|1	30|2	11|10
Chr10	48848832	48848832	T	A	SNP	0|0	0|1	14|0	3|12
Chr10	48848919	48848919	C	G	SNP	0|0	0|1	16|0	2|15
Chr10	48848927	48848927	C	A	SNP	0|0	0|1	15|0	4|14
Chr10	48848999	48848999	C	T	SNP	0|0	0|1	16|1	3|6
Chr10	48850139	48850139	T	G	SNP	0|0	0|1	24|0	3|5
Chr10	48850199	48850199	G	A	SNP	0|0	0|1	24|0	3|5
Chr10	48852907	48852907	T	G	SNP	0|0	0|1	42|2	7|13
Chr10	48853600	48853600	A	G	SNP	0|1	0|1	40|8	4|9
Chr10	48854062	48854062	A	C	SNP	0|1	0|1	28|6	3|8
Chr10	48854596	48854596	G	A	SNP	0|1	0|1	33|3	3|5
Chr10	48854597	48854597	T	C	SNP	0|1	0|1	33|3	3|5
Chr10	48855564	48855564	A	T	SNP	0|1	0|1	49|5	8|17
Chr10	48855599	48855599	T	G	SNP	0|1	0|1	48|4	6|13
Chr10	48855906	48855906	C	A	SNP	0|1	0|1	38|5	4|7
Chr10	48856445	48856445	T	C	SNP	0|1	0|1	34|7	2|10
Chr10	48857431	48857431	C	T	SNP	0|1	0|1	28|6	2|8
Chr10	48857549	48857549	C	A	SNP	0|0	0|1	33|2	3|5
Chr10	48858087	48858087	T	G	SNP	0|1	0|1	30|4	3|12
Chr10	48858331	48858331	G	A	SNP	0|0	0|1	24|1	4|5
Chr10	48858983	48858983	A	G	SNP	0|1	0|1	45|9	3|9
Chr10	48860124	48860124	A	G	SNP	0|1	0|1	40|4	5|15
Chr10	48860256	48860256	A	C	SNP	0|0	0|1	36|1	3|11
Chr10	48860404	48860404	G	A	SNP	0|0	0|1	40|2	4|10
Chr10	48860429	48860429	C	G	SNP	0|0	0|1	41|0	5|10
Chr10	48860704	48860704	A	G	SNP	0|1	0|1	40|5	2|16
Chr10	48862087	48862087	T	C	SNP	0|1	0|1	40|5	3|9
Chr10	48862318	48862318	A	C	SNP	0|1	0|1	24|3	2|10
Chr10	48862486	48862486	A	T	SNP	0|1	0|1	11|1	4|7
Chr10	48864064	48864064	G	A	SNP	0|0	0|1	15|0	2|7
Chr10	48864187	48864187	G	A	SNP	0|0	0|1	16|0	2|11
Chr10	48864193	48864193	A	C	SNP	0|0	0|1	15|0	2|10
Chr10	48865093	48865093	G	T	SNP	0|1	0|1	36|4	5|9
Chr10	48866898	48866898	A	T	SNP	0|0	0|1	37|1	3|10
Chr10	48867730	48867730	G	A	SNP	0|1	0|1	34|5	4|7
Chr10	48867849	48867849	A	T	SNP	0|1	0|1	44|13	16|7
Chr10	48867853	48867853	C	T	SNP	0|1	0|1	48|13	16|8
Chr10	48867856	48867856	C	T	SNP	0|1	0|1	47|13	16|8
Chr10	48867879	48867879	T	C	SNP	0|1	0|1	51|13	16|7
Chr10	48867917	48867917	T	G	SNP	0|1	0|1	51|13	14|11
Chr10	48867926	48867926	G	A	SNP	0|1	0|1	51|12	16|11
Chr10	48867965	48867965	G	C	SNP	0|1	0|1	53|15	22|12

Chr10	48867973	48867973	G	A	SNP	0|1	0|1	49|13	21|9
Chr10	48867989	48867989	A	T	SNP	0|1	0|1	43|11	17|8
Chr10	48868737	48868737	C	T	SNP	0|0	0|1	22|0	5|5
Chr10	48869140	48869140	C	A	SNP	0|1	0|1	43|4	3|24
Chr10	48870279	48870279	A	C	SNP	0|1	0|1	52|10	26|12
Chr10	48870283	48870283	A	T	SNP	0|1	0|1	52|11	25|12
Chr10	48870289	48870289	G	A	SNP	0|1	0|1	51|13	23|17
Chr10	48870309	48870309	C	T	SNP	0|1	0|1	52|20	18|17
Chr10	48870363	48870363	T	C	SNP	0|1	0|1	54|18	21|13
Chr10	48870364	48870364	G	A	SNP	0|1	0|1	53|18	21|13
Chr10	48870369	48870369	C	T	SNP	0|1	0|1	54|18	20|13
Chr10	48871689	48871689	A	T	SNP	0|1	0|1	74|10	3|7
Chr10	48873043	48873043	A	T	SNP	0|1	0|1	36|9	5|4
Chr10	48877522	48877522	G	T	SNP	0|0	0|1	55|3	7|9
Chr10	48878389	48878389	G	A	SNP	0|1	0|1	38|5	7|9
Chr10	48878432	48878432	T	A	SNP	0|1	0|1	38|5	6|6
Chr10	48878856	48878856	A	C	SNP	0|1	0|1	38|3	2|13
Chr10	48879090	48879090	A	G	SNP	0|0	0|1	45|2	4|15
Chr10	48879242	48879242	A	T	SNP	0|1	0|1	39|4	6|8
Chr10	48879423	48879423	T	C	SNP	0|1	0|1	17|2	2|9
Chr10	48879722	48879722	A	C	SNP	0|1	0|1	26|2	6|6
Chr10	48880053	48880053	A	C	SNP	0|1	0|1	54|5	14|20
Chr10	48880054	48880054	G	A	SNP	0|1	0|1	55|5	14|20
Chr10	48880642	48880642	A	T	SNP	0|0	0|1	22|1	5|16
Chr10	48880914	48880914	A	G	SNP	0|1	0|1	13|4	2|6
Chr10	48881297	48881297	A	T	SNP	0|1	0|1	19|2	3|5
Chr10	48881726	48881726	C	T	SNP	0|0	0|1	77|6	5|22
Chr10	48882656	48882656	A	T	SNP	0|1	0|1	33|3	3|6
Chr10	48884053	48884053	C	G	SNP	0|0	0|1	73|4	9|10
Chr10	48884601	48884601	A	G	SNP	0|1	0|1	36|4	8|16
Chr10	48884850	48884850	C	G	SNP	0|1	0|1	27|8	6|9
Chr10	48884937	48884937	C	T	SNP	0|1	0|1	21|4	3|5
Chr10	48885296	48885296	A	G	SNP	0|1	0|1	48|5	10|16
Chr10	48886114	48886114	C	A	SNP	0|0	0|1	47|3	4|16
Chr10	48886423	48886423	C	T	SNP	0|0	0|1	59|3	8|15
Chr10	48886565	48886565	G	A	SNP	0|0	0|1	31|2	4|9
Chr10	48886569	48886569	T	C	SNP	0|0	0|1	30|2	3|9
Chr10	48886618	48886618	T	C	SNP	0|0	0|1	26|1	2|8
Chr10	48887673	48887673	A	G	SNP	0|1	0|1	9|4	2|8
Chr10	48887697	48887697	T	C	SNP	0|1	0|1	10|4	2|9
Chr10	48887710	48887710	A	G	SNP	0|1	0|1	9|4	3|11
Chr10	48887720	48887720	T	G	SNP	0|1	0|1	10|4	3|11

Chr10	48887749	48887749	A	G	SNP	0|1	0|1	12|7	3|17
Chr10	48887774	48887774	A	G	SNP	0|1	0|1	14|5	2|17
Chr10	48887795	48887795	G	T	SNP	0|1	1|1	15|5	1|17
Chr10	48887862	48887862	A	T	SNP	0|1	1|1	13|3	0|14
Chr10	48887863	48887863	C	T	SNP	0|1	1|1	13|3	0|14
Chr10	48887864	48887864	A	T	SNP	0|1	1|1	13|3	0|14
Chr10	48887942	48887942	A	C	SNP	0|1	0|1	14|2	2|10
Chr10	48888267	48888267	G	A	SNP	0|1	0|1	37|10	4|7
Chr10	48888390	48888390	C	G	SNP	0|1	0|1	46|7	7|7
Chr10	48888486	48888486	A	G	SNP	0|1	0|1	39|5	6|12
Chr10	48888501	48888501	A	C	SNP	0|1	0|1	46|6	6|17
Chr10	48891654	48891654	G	A	SNP	0|0	0|1	79|2	12|16
Chr10	48891868	48891868	T	C	SNP	0|0	0|1	48|0	2|9
Chr10	48891964	48891964	T	A	SNP	0|0	0|1	35|0	4|7
Chr10	48892661	48892661	C	G	SNP	0|1	0|1	50|9	12|10
Chr10	48892682	48892682	T	C	SNP	0|1	0|1	44|12	10|12
Chr10	48892700	48892700	T	C	SNP	0|1	0|1	40|12	8|13
Chr10	48893062	48893062	A	G	SNP	0|0	0|1	50|2	11|12
Chr10	48894996	48894996	C	A	SNP	0|1	0|1	35|8	8|7
Chr10	48895612	48895612	C	T	SNP	0|1	0|1	43|5	9|11
Chr10	48895697	48895697	T	A	SNP	0|0	0|1	40|1	5|4
